# Further analysis and refinements of the perceived stressors in intensive care units (PS-ICU) scale: a French nation-wide cross-sectional multicentre study

**DOI:** 10.1186/s13613-025-01572-7

**Published:** 2025-11-20

**Authors:** Florent Lheureux, Maxime Jollivet, Juliette Chiron, Sarah Poulet, Alicia Fournier, Gilles Capellier, Laetitia Bodet-Contentin, Antoine Herault, Joffrey Hamam, Pascal Beuret, Pierre-Alexandre Lamizet, Mathieu Schoeffler, Bérengère Vivet, Christophe Guitton, Gaël Piton, Justine Perrot, Khaldoun Kuteifan, Céline Guichon, Hodane Yonis, Olivier Barbot, Carole Schwebel, Pierre-Yves Olivier, Enora Atchade, Alexis Dürr, Frédérique Schortgen, Claire Bourel, Diane Friedman, Caroline Hauw-Berlemont, Laura Federici, Anne-Sophie Muller, Kada Klouche, Charles Damoisel, Sabine Valera, Ghada Sboui, Cathy Lemaitre, Antonin Michaud, Alexandra Beurton, Emeline Buttazzoni, Camille Aïtout, Bérengère Araujo, Laurence Goncalves, Sylvie Canon, Anne Couvillers, Anne-Laure Poujol, Juliette Toulet, Belaïd Bouhemad, François Aptel, Cyril Goulenok, Alexandra Laurent, the PS-ICU Group, Maxime Granier, Clémentine Hoareau, Isabelle Bourgoin, Fabienne Tamion, Emilie Saint Leger, Déborah Hiron, Hubert Grand, Aude Londeix, Céline Marconnet, Florent Montini, Laetitia Volle, Karine Herbert, Corinne Mourioux, Laurence Groshenry, Claire Chagnat, Melissa Crotet, Juliette Meunier, Séverine Guillarme, Hadrien Winiszewski, Sarah Noir, Laure Clouet, Francis Augier, Isabelle Zamofing, Jean-Cyril Van Hamme, Marie-Anne Tisserand, Jean-Christophe Richard, Mathieu Rulliere, Emmanuelle Rouve, Florence Thevenot, Aurélie Maillach, Eve Merzouki, Carole Haubertin, Philippe Montravers, Aurélie Gouel-Cheron, Mathilde Dijoux, Martial Thyrault, Léopoldine Legrain, Cécilia Tabra Osorio, Emilie Aebischer, Raphaël Favory, Evelyne Egret, Djillali Annane, Anne Robin, Ségolène Jourdier, Sarah Ferte, Laurent Serpin, Cécile Capelli, Alice Preault, Delphine Daubin, Corinne Pelle, Claudine Gniadek, Stéphanie Galia, Jean-Marie Forel, Sabine Janowski, Jerôme Vandensteen, Julien Poissy, Vincent Castelain, Sylvie L’Hotellier, Jeanne Sovant, Muriel Fartoukh, Laurence Krzyzaniak, Pierre-Grégoire Guinot, Christelle Jault, Armand Mekontso Dessap, Maryline Couette, Sébastien Preau, Saadalla Nseir, Sandrine Ndinga Mondze, Mercedes Jourdain

**Affiliations:** 1 Laboratoire de Psychologie (UR 3188), Université Marie et Louis Pasteur, 25000 Besançon, France; 2 Laboratoire Bonheurs, CY Cergy Paris Université, Cergy, France; 3 Réanimation Polyvalente, Centre Hospitalier d’Arras, Arras, France; 4 Laboratoire de Psychologie Dynamiques Relationnelles et Processus Identitaires (Psy-DREPI), Université Bourgogne Europe, Dijon, France; 5 Service Anesthésie-Réanimation-Douleur, Centre Hospitalier La Miséricorde, Ajaccio, France; 6 Médecine Intensive Réanimation, Centre Hospitalier Universitaire de Tours, Paris, France; 7 Centre d’Investigation Clinique de Tours (CIC1415), INSERM, Tours, France; 8 CRICS-Triggersep F-CRIN research network, Tours, France; 9 INSERM, SPHERE, UMR1246, Université de Tours et Nantes, Tours et Nantes, France; 10 Médecine Intensive Réanimation, Hôpital Charles Nicolle, Rouen, France; 11 Laboratoire EnVI, INSERM UMR1096, Université de Rouen Normandie, Rouen, France; 12 Réanimation Polyvalente, Hôpital R. Boulin, Libourne, France; 13 Réanimation et Soins continus, Centre Hospitalier de Roanne, Roanne, France; 14 Réanimation Polyvalente, Centre Hospitalier d’Arles, Arles, France; 15 Service de Réanimation Soins Critiques, Groupement Hospitalier Portes de Provence, Montélimar, France; 16 Réanimation Polyvalente, Groupe Hospitalier de la Haute-Saône, Vesoul, France; 17 Réanimation Médico-Chirurgicale, Centre Hospitalier Le Mans, Le Mans, France; 18 Service de Médecine Intensive Réanimation, Centre Hospitalier Universitaire de Besançon, Besançon, France; 19 Department of Anesthesiology and Intensive Care, Dijon University Hospital, 21000 Dijon, France; 20 Service de Réanimation Médicale GHRMSA, Hôpital Emile Muller, Mulhouse, France; 21 Service de Réanimation Chirurgicale, Groupement Hospitalier Nord, Hospices Civils de Lyon, Lyon, France; 22 Médecine Intensive Réanimation, Hôpital de la Croix-Rousse, Hospices Civils de Lyon, Lyon, France; 23 Réanimation Polyvalente, Centre Hospitalier de Perpignan, Perpignan, France; 24 Médecine Intensive Réanimation NPT - RH, Centre Hospitalier Universitaire Grenoble Alpes, Site NORD, La Tronche, France; 25 Département de Médecine Intensive Réanimation et Médecine Hyperbare, Centre Hospitalier Universitaire d’Angers, Angers, France; 26 DMU PARABOL, APHP, Centre Hospitalier Universitaire Bichat-Claude Bernard, Paris, France; 27 Médecine Intensive et Réanimation, Groupe Hospitalier Nord Essonne, Paris Saclay, France; 28 Médecine Intensive et Réanimation, Centre Hospitalier Intercommunal de Créteil, Créteil, France; 29 Service de Médecine Intensive Réanimation, Centre Hospitalier Universitaire de Lille, Lille, France; 30 Médecine Intensive Réanimation, Hôpital Raymond Poincaré, APHP, Paris, France; 31 Médecine Intensive Réanimation, Hôpital européen Georges Pompidou, APHP, Paris, France; 32 Service d’Anesthésie-Réanimation-Douleur, Centre Hospitalier La Miséricorde, Ajaccio, France; 33 Réanimation Polyvalente, Centre Hospitalier de Bastia, Bastia, France; 34 Médecine Intensive et Réanimation, Hôpital Lapeyronie, Centre Hospitalier Universitaire de Montpellier, Montpellier, France; 35 Phymedexp, Faculty of Medicine, Université de Montpellier, Inserm, Centre National de Recherche Scientifique (CNRS), CHRU de Montpellier, Montpellier, France; 36 Réanimation Polyvalente-Unité de Soins Intensifs Polyvalents, Université Paris Saclay, Hôpital Antoine Béclère AP-HP, Paris, France; 37 Hôpital Nord, Médecine Intensive Réanimation, Centre Hospitalier Universitaire, Assistance Publique des Hôpitaux de Marseille, Marseille, France; 38 Médecine Intensive Réanimation, Centre Hospitalier de Béthune, Béthune, France; 39 Hôpital de Hautepierre, Médecine Intensive et Réanimation, Centre Hospitalier Régional Universitaire de Strasbourg, Strasbourg, France; 40 Service de Médecine Intensive Réanimation, Hôpital Tenon, APHP, Paris, France; 41 UMRS 1158 Neurophysiologie Respiratoire Expérimentale et Clinique, Sorbonne Université, Paris, France; 42 Département Anesthésie Réanimation Médecine Périopératoire, Centre Hospitalier Universitaire de Toulouse, Toulouse, France; 43 Médecine Intensive Réanimation, Centre Hospitalier Universitaire Henri Mondor, APHP, Créteil, France; 44 Equipe VCR, Ecole de psychologues Praticiens, Paris, France; 45 Service de Réanimation Chirurgicale Polyvalente, Groupe Hospitalier Pitié Salpêtrière, APHP, Paris, France; 46 Réanimation polyvalente, Centre Hospitalier Jura Sud, Lons-le-Saunier, France; 47 Médecine Intensive Réanimation, Hôpital privé Jacques Cartier, Ramsay Santé, Massy, France

**Keywords:** Job stressors, Intensive care units, Scale, Validity, Reliability, Invariance

## Abstract

**Background:**

Assessing sources of job stress in intensive care units is a critical issue for preventing many occupational health and care-related issues, such as burnout, voluntary turnover and decrease in quality and safety of care. Accordingly, this French nation-wide multicentre study aims to provide supplementary evidence regarding the validity of a recent tool: the Perceived Stressors in Intensive Care Units (PS-ICU) scale. More precisely, this study has three main objectives: to 1) confirm the metrological properties of the PS-ICU scale on a large sample of professionals; 2) test its measurement invariance between nurses, physicians and residents (initial population targeted by the scale); 3) examine whether the scale would also be suited for use with nursing auxiliaries. In addition, depending on the results (which may suggest the removal of several items), this study offers the possibility to shorten the scale to facilitate its use.

**Method and results:**

2241 ICU professionals (1135 nurses, 308 physicians, 179 residents, and 619 nursing auxiliaries; overall participation rate of 58.10%) from 42 ICUs in France, voluntarily completed an online questionnaire collecting socio-demographic data and perceived job stressors (PS-ICU). Exploratory structural equation modelling (ESEM), unidimensional reliability (McDonald’s Omega) and item response theory (IRT) analyses overall confirmed the metrological properties of the scale, while several items were removed and the sixth factor (“lack of support and resources from the organisation”) measured by the scale was revised. Results regarding measurement invariance show that the PS-ICU scale can be used to compare occupational groups, including nursing auxiliaries. Finally, all analyses resulted in a reduction of the scale to a 26-item version.

**Conclusions:**

The PS-ICU scale, which measures generic and ICU-specific job stress factors, is a valid and reliable scale that can be used to collect data from nurses, physicians and residents, as well as from nursing auxiliaries. With 26 items, it can be used by researchers and managers in ICUs to assess the extent and type of stress factors perceived by healthcare professionals.

**Supplementary Information:**

The online version contains supplementary material available at 10.1186/s13613-025-01572-7.

## Background

Professionals in intensive care units (ICUs) have to deal with patients suffering from serious pathologies requiring complex diagnostic and therapeutic procedures [1]. This leads to demands for specific skills and knowledge, an overload of responsibilities and a heavy workload [2]. End-of-life issues, interpersonal conflicts, perception of futility and staff unwillingness to withdraw life sustaining treatment are major sources of stress. These stressors have a real impact on the occupational health of professionals, with the prevalence of burnout among physicians and nurses in these units considered very high [3], and quality of work life low [4, 5].

This context underlines the importance of identifying stress factors in ICUs, and equipping ourselves with valid tools specific to the ICU work context. However, following a review of 59 tools used in 102 studies, Laurent et al. [6], pointed out that none of these tools both meets the criteria of metrological validity and of relevance with regard to the specificity of work in ICUs. As a result, a new tool has been developed to overcome these limitations: the Perceived Stressors in Intensive Care Units (PS-ICU) scale [7]. This 50-item scale measures both ICU-specific stressors and generic workplace stressors in a hospital setting. It assesses the presence and perceived intensity of six major stress factors: 1) lack of fit with families and organisational functioning; 2) patient and family-related emotional load; 3) complex/at risk situations and skill-related issues; 4) workload and human resource management issues; 5) difficulties related to team working; and 6) suboptimal care situations. It was drawn up jointly in three languages (i.e., French, Spanish, and Italian) following a series of 165 semi-structured interviews with ICU physicians, residents and nurses from France, Spain, Italy and Canada (French-speaking), and following a survey conducted on a sample of 497 professionals from 16 ICUs in France, Spain and Italy, with satisfactory factorial validity, internal consistency, test-retest reliability, convergent validity with Karasek’s job content questionnaire [8] and concurrent validity with the Maslach Burnout Inventory [9]. Since then, this tool has been translated and adapted for a Chinese context with satisfactory metrological properties [10, 11]. Additionally, this scale has proved efficient in examining the association of chronic job stress with the development of burnout, post-traumatic stress disorders and the impairment of overall mental health during the Covid-19 crisis [12–14].

Henceforth, in order to confirm previous results and to reinforce the study of the metrological properties of the PS-ICU, there is a need to conduct other studies on large samples of professionals. In particular, it is necessary to examine whether the scale measures perceived stressors across subgroups similarly (e.g., participants having different occupations), metrological invariance being a necessary condition for reliable group-comparisons [15]. Moreover, the validity of the scale has not been tested on nursing auxiliaries, which is another important group of ICU professionals. Accordingly, the study reported below pursued three main objectives:

- to confirm the metrological properties of the scale on a large sample of professionals;
- to test its measurement invariance between nurses, physicians and residents (initial population targeted by the scale);
- to examine whether the scale would also be suited for use with nursing auxiliaries.

In addition, depending on the results (which may suggest the removal of several items), this study offers the possibility to shorten the scale to facilitate its use for both research and preventive purposes.

## Methods

### Participants

Over a period of five months, 2241 ICU professionals (1135 nurses, 308 physicians, 179 residents, and 619 nursing auxiliaries) voluntarily participated in a cross-sectional multicentre study, with an overall participation rate of 58.10%. They were recruited from 42 ICUs in France. To recruit these ICUS, we first create a contact list of professionals that could be approached (e.g., head of the unit), using the professional networks of the main authors, as well as using information available on the institutional websites of ICUs for which we did not identify an already known contact. We did this in view to contact services geographically situated on all mainland France. The invitation to participate in the study was also made public among the members of the *Société de Réanimation de Langue Française* (Website, Newsletter) and on the social networks of the One O One fund (1O1).

### Procedure and material

We collected data from February 2024 to June 2024. At a predefined date, the principal investigator within the unit informed professionals about the study via an oral message or via an email sent to their professional inbox (using the unit internal mailing list). Therefore, we did not collect emails of the participants. This message provided information regarding the aim of the study, their rights as participants, and invited them to voluntarily complete the online questionnaire on the LimeSurvey platform [16]. Participants had three weeks to complete it, with follow-up e-mails sent by the principal investigator after the first and second weeks to increase participation. Upon agreeing to participate, professionals were asked to complete socio-demographic data (e.g., gender, occupation, professional seniority, responsibilities; see Table 1 for the complete list) and the PS-ICU scale [7]. The PS-ICU assesses the six major stress factors described in the introduction section. Each item was rated on a 5-point scale ranging from 0 ("Not experienced") to 4 ("I experienced this situation and felt very stressed"). For each stress factor, the mean score of the corresponding items can be calculated. Additionally, an overall stress score can be computed as the average of all 50 items.

**Table 1:** Characteristics of the 2199 participants and of the 42 intensive care units (ICUs) included in the analyses (frequency and percentages)

		Total	% valid	Missing values
ICU type	Medical ICU in a university hospital (CHU^a^) (*N*=17 ICUs)	886	46	253^c^
	General ICU in a general, non-university hospital (CH^b^) (*N* = 14 ICUs)	689	35	
	Other contexts: surgical, or medical ICU in a general hospital (CH) or general ICU in a university hospital (CHU) (*N* = 11 ICUs).	371	19	
Other working context information	Works in a High Dependency Unit (HDU)	1455	66	7
	Works on multiple sites	198	9	0
Age	18–34 years	1141	52	0
	35–49 years	802	36	
	50 years and over	256	12	
Gender	Women	1629	74	6
	Men	564	26	
Family status	Living in a couple	1916	87	0
	Has children	1075	49	0
Occupation	Nurses	1118	51	0
	Nursing auxiliaries	608	28	
	Physicians	302	14	
	Residents	171	8	
Job contract	With permanent contract status	1763	80	0
	Full-time	1946	88	0
Length of service in the unit	Under 3 years	823	38	0
	Between 3 and 5 years	405	18	
	More than 5 years	971	44	
Length of service in intensive care	less than 1 year	277	13	0
Work pattern	Day work	418	19	0
	Night work	262	12	
	Does both	1519	69	
On-call duties	Yes	1821	83	0
	If yes, make 12-h shifts	1418	64	0
	If yes, make 24-h shifts	448	20	
	If yes, 1 to 9 times per month	589	27	0
	If yes, 10 to 12 times per month	781	36	
	If yes, 13 times or more per month	451	20	
Has one or more responsibilities	Yes	881	40	0
	If yes, one	585	27	
	If yes, two or more	296	13	

^a^Centre hospitalier universitaire

^b^Centre hospitalier

^c^This quite high number is due to the fact that participants were free to *not* enter the anonymity code of their unit

In addition to the main survey, the head of the unit provided information regarding their unit, in particular the number of nurses, physicians, residents and nursing auxiliaries working within the unit at the time of the study. This allowed us to estimate the participation rates (overall and for each occupational group).

### Statistical analyses

#### Data preparation and descriptive characterisation of the sample

Participants with more than 5 missing responses on the PS-ICU scale were excluded from the study. When fewer than five responses were missing, we imputed missing data using the multiple imputation by chained equations technique [17]. We also checked for multivariate outliers using the Mahalanobis distance [18] and the local outlier factor techniques [19] (participants identified as “outliers” by both techniques were excluded). These preliminary steps resulted in the exclusion of 42 participants (2199 participants left). Then, we examined descriptive statistics (frequencies, percentages) to describe the general characteristics of the sample.

#### Confirmation of the metrological properties of the scale on the original target population

Then, we examined the metrological properties of the PS-ICU scale on the data collected from its original target population (i.e., nurses, physicians and residents, total *N* = 1591). To confirm the factor structure of the PS-ICU scale, we conducted full exploratory structural equation modelling (ESEM) [20]. We preferred this method to confirmatory factor analysis, because it is more suited to estimating the measurement model of factors that share a common conceptual meaning [21] (here “exposure to stress”), and thus allow items to have loadings different from zero for several factors. We applied the weighted least squares estimation technique to the polychoric correlation matrix, because of the ordinal nature of the data and the non-normal distributions observed for several items.

Several models were compared, and model fit was assessed using a combination of several indices of equal importance, such as the Chi-square (χ^2^) test (the lower the value, the better is the model fit), the Root Mean Square Error of Approximation (RMSEA) (values ≤ 0.05 for good fit and < 0.08 for acceptable fit), the Comparative Fit Index (CFI) (values ≥ .95 for acceptable fit ≥ .97 for good fit), and the Standardized Root Mean Square Residual (SRMR) (values ≤ 0.05 for good fit and < 0.10 for acceptable fit) [22]. For χ^2^, RMSEA and CFI we used scaled values to account for violations of the normality assumption. Additionally, for each item we examined factors loadings, with values ≥ .30 indicative of substantial item-factors associations. Factor loadings represent the extent to which each item measure each factor (the higher the factor loading is, the more the item measure the given factor). Ideally, each item should have a unique strong association with the expected factor. We first examined the fit indices obtained for the original six-factor model estimated on a 43-item version of the scale, in line with the results of Laurent et al. [7] suggesting that seven items should be removed to estimate these specific factors. Given that all items taken together can be also used to estimate an overall exposure to stress, we also estimated fit indices for a general stress one-factor model using the 50 items. Better indices for the six-factor model against the one-factor model would support Laurent et al. [7] observations. To complement ESEM results, we assessed internal consistency for each factor using McDonald’s omega coefficient (≥ 0.70) [23].

Then, as the results obtained for these two models (composition of the factors and item loadings) were not fully satisfying, seven alternative models with fewer items and/or one factor less were tested iteratively to improve fit (i.e., decreasing χ^2^, RMSEA, SRMR and increasing CFI), until an optimal configuration was obtained for this population, while removing the items with insufficient loadings (<.30), cross-loadings or loading on another factor. In particular, the results of the six-factor model estimated on the 43-item version of the scale, led to the testing of an alternative five-factor model (removing the sixth factor), followed by several derived models with a progressively reduced number of items. Concurrently, an alternative six-factor model was tested, with a revision of the sixth factor to take into account the fairly significant correlations between four items referring to inadequate resources, both material and human, and support from the hospital (lack of support and resources from the organisation). These items were initially associated with factors 1, 4 and 6 (#6 “Shortage of beds in the unit", #15 "Absence of administrative support", #32 "Lack of staff" and #8 "Unsuitable or under-equipped treatment area or defective equipment") [7].

Following theses analyses, a five-factor, 30-item model and a six-factor, 31-item model were considered satisfying (best fit indices and item loadings) and were thus retained for subsequent Item Response Theory (IRT) analyses [24]. The IRT methodology examines whether a given item is informative and reliable to estimate its target factor. This methodology also permits to compare competing models. We employed the graduated response approach [25], because it is particularly well suited to items with multiple ordinal (polytomous) responses and because it systematically obtained lower AIC (Akaike Information Criterion) values than the other IRT approaches available for this type of item. We examined the discrimination parameters for each item (≥ 0.5, ideally between 1 and 2), as well as their fit to the model via the RMSEA (values ≤ 0.05 for good fit and < 0.08 for acceptable fit). We also estimated the empirical marginal reliability for each factor (values ≥ 0.70) and examined the test information function.

#### Test of measurement invariance

We also used ESEM to examine the measurement invariance of the scale across occupational groups (i.e., nurses, physicians, residents). Estimating invariance has generally four steps [15]: 1st test of configural invariance (i.e., equivalence of model form); 2nd metric invariance (i.e., equivalence of factor loadings); 3rd scalar invariance (i.e., equivalence of item intercepts or thresholds); and 4th strict invariance (i.e., equivalence of items' residuals or unique variances). We used the same fit indices at each step (χ^2^, RMSEA, CFI, SRMR), and they must be satisfying (see the interpretation criteria provided before), and stable across steps (differences in fit indices, symbolised by the Greek letter ‘Delta’ Δ, must be the lowest possible: Δχ^2^, ΔRMSEA, ΔCFI, ΔSRMR). Satisfactory results following the three-first steps are pre-requisite for comparing group means on the latent factor(s) measured by the scale. We estimated invariance for the six-factor, 31-item model, and for the five-factor, 30-item model, as they were the best models in previous ESEM and IRT analyses.

Unfortunately, the samples sizes of physicians (*N* = 302) and residents (*N* = 171) were insufficient to distinguish between them. Thus, as residents are future physicians involved in the daily care of patients, who take calls facing night work etc. they were merged with senior physicians, and compared to nurses (*N* = 1118). Although this is suboptimal, about 46% of included residents had more than one year of experience, with increased medical responsibilities. Moreover, for the same reason, it was not possible to estimate strict invariance correctly (too many parameters to estimate).

#### Estimation of the validity, reliability and invariance of the scale for nursing auxiliaries

Then, we sought to determine whether the scale would be suited for nursing auxiliaries (*N* = 608). We also used ESEM and IRT, with the six-factor, 31-item model as reference, because it has obtained better indices than all other models (including the five-factor, 30-item model), in previous analyses conducted on the initial population of the scale (i.e., nurses, physicians, residents). We assessed the measurement invariance between this initial population and the nursing auxiliaries group using the same procedure as describe before. These analyses led to the removal of five other items, as they were not invariant when including nursing auxiliaries.

#### Analyses to determine the optimal length of the scale to assess general stress level

As stated before (see end of the introduction), this study offers the possibility to reduce the scale through the removal of problematic items. In line with this claim, the previously described analyses suggest the removal of several items in addition to the 7 items previously identified as potentially superfluous by Laurent et al. [7]. Given that PS-ICU allows overall exposure to stress to be assessed by averaging all the items—in addition to assess separate stress factors—this raises two concomitant questions: does a reduced version assess general stress as well as the full 50-item scale? Do any of the potentially removed items assess general stress substantially and should therefore be retained in the scale for this purpose only? To answer these questions, we compared the results obtained for the full scale (50 items) with the results obtained for two alternative shorter versions, the 26-item version obtained after all previous analyses and a slightly longer version including the same items plus the five items (#2, #12, #19, #36 and #44) that obtained the best mean rankings over the whole sample on three criteria: high factor loading (ESEM) and high discrimination parameter (IRT) when a single "general stress" factor was extracted, as well as a high mean perceived stress score (see supplementary material C). We compared the descriptive statistics (mean, standard deviation, skewness, kurtosis etc.) obtained by these three versions when estimating the general stress level of professionals. In addition, we examined Pearson correlation coefficients between the full 50-item version and the two shorter versions. If a reduced scale obtains statistics similar to those of the full 50-item scale and is very strongly correlated with it (*r* >.90), it would be considered suited for estimating general stress as well. Moreover, if using the five items that best represent general stress level increases the similarity of descriptive statistics and the correlation between the shorter and the full version of the scale, it would be an argument for retaining them for that purpose.

Statistical analyses were performed using R Studio [26] with the packages “mice“, ”dbscan”, ”stats”, ”esem”, ”psych”, ”lavaan”, ”mirt” and using the JASP software [27].

## Results

### Characteristics of the participants included

Table 1 provides a statistical summary of the characteristics of the sample. This table illustrates the diversity of the personal and professional profiles of the participants included, with regards to numerous characteristics. Even though for some variables majority trends appear, for each of their modalities we included more than one hundred participants, thus contributing to the generalisability of the results. However, participation rates varied according to occupational groups, χ^2^(3) = 112.05, *p* <.0001, physicians participated more (74.21%) than all other occupational groups, whereas nursing auxiliaries were the less included (45.60%), with nurses (61.16%) and residents (62.50%) included at an intermediate level.

### Metrological properties of PS-ICU for its initial population (nurses, physicians, residents)

#### Results of exploratory structural equation modelling (ESEM) and internal consistency (McDonald Omega)

As shown in Table 2, the initial six-factor, 43-item model of Laurent et al. [7] obtained satisfactory fit indices, whereas for the one-factor model these were well below the expected criteria. However, an examination of the composition of the factors and factor loadings of the six-factor, 43-item model (see supplementary material A) revealed that the factor 6 obtained did not correspond to that identified by Laurent et al. [7]. The loadings were rather low, the whole was not very homogenous and other items were loading on it. Because of this observation, alternative five-factor and six-factor models with fewer items were tested (see supplementary material A for all results). Looking at the fit indices (Table 2) and factor loadings, the six-factor, 31-item and five-factor, 30-item models appeared to be the most satisfactory, although the fit indices were slightly better for the six-factor model. It should be noted that the Omega internal consistency indices were systematically satisfactory (ω ≥ .70).

**Table 2:** Fit indices obtained using exploratory structural equation modelling (ESEM) and McDonald's Omega internal consistency for the main models tested on data on nurses, physicians and residents

Model tested	Chi-square scaled			CFI scaled	RMSEA scaled	SRMR	Omega coefficient					
	χ^2^	*df*	χ^2^/*df*		Value [90%CI]		Factor 1	Factor 2	Factor 3	Factor 4	Factor 5	Factor 6
One general stress factor with all 50 items	12040.47	1176	10.24	0.800	0.076 [0.075; 0.077]	0.071	0.92	–	–	–	–	–
Original 6-factor with 43 items (general stress items removed)	2738.85	666	4.112	0.955	0.044 [0.043; 0.046]	0.030	0.82	0.83	0.81	0.81	0.73	0.74
Revised 5-factor with 30 items (removed items: general stress items, original 6th factor's items, and #48, #49, #15 #1, #44, #24, #46)^a^	1744.91	325	5.369	0.954	0.052 [0.050; 0.055]	0.031	0.81	0.78	0.81	0.76	0.73	–
Revised 6-factor with 31 items (removed items: general stress items and #48, #49, #2, #11, #12, #19, #24, #33, #36, #44, #46, #1)^a^	1379.14	294	4.691	0.964	0.048 [0.046; 0.051]	0.026	0.79	0.78	0.81	0.71	0.73	0.70

See the supplementary material A for the results obtained for all models. *df* is for ‘degrees of freedom’. Criterion for model assessment: lowest Chi-square value (χ^2^); Comparative Fit Index (CFI) ≥ 0.95, Root Mean Square Error of Approximation (RMSEA) ≤ 0.08, Standardised Root Mean squared Residual (SRMR) ≤ 0.10 for acceptable fit; CFI ≥ 0.97, RMSEA ≤ 0.05, SRMR ≤ 0.05 for good fit; Omega ≥ 0.70

^a^Models chosen for IRT and measurement invariance analyses

Table 3 shows the factor loadings and explained variances of items for these two most satisfactory models (i.e., with the best fit indices and factor loadings). At this level, we can compare our results with that of Laurent et al.[7] (do we replicated them?). The first five observed factors correspond to those obtained by these authors (i.e., same items clustered into the same factors, factor loadings ≥. 30), but sometimes with fewer items (for factors 1, 2 and 4). For instance, items #18 (“Having to execute care tasks quickly in emergency cases”), #40 (“Treating complex or serious pathologies”), #16 (“Risk of error, fear of doing a poor job”), #37 (“Having to perform tasks for which I lack knowledge or skills”) and #25 (“Patient who deteriorates in an unexpected or unexplained manner”) all had a unique loading ≥ .30 (see bolded values) on the expected third factor (“complex/at risk situations and skill-related issues”), either with the five-factor, 30-item model, or the six-factor, 31-item model. This is consistent with the results of Laurent et al. [7].

**Table 3:** Factor loadings and explained variances for the six-factor, 31-item model and the five-factor, 30-item model for nurses, physicians and residents

6-factor, 31-item model									5-factor, 30-item model							
Item number	Original factor^a^	Fact.1	Fact.2	Fact.3	Fact.4	Fact.5	Fact.6	*R2*	Item number	Original factor	Fact.1	Fact.2	Fact.3	Fact.4	Fact.5	*R2*
10	F1	**0.70**	0.08	−0.13	−0.09	0.07	0.01	0.50	10	F1	**0.70**	0.10	−0.11	−0.13	0.08	0.49
27	F1	**0.69**	0.16	0.08	−0.04	0	−0.19	0.56	27	F1	**0.63**	0.17	0.18	−0.19	−0.04	0.53
42	F1	**0.68**	−0.13	0.2	0.04	−0.1	0.02	0.48	42	F1	**0.73**	−0.14	0.25	−0.04	−0.11	0.49
4	F1	**0.57**	−0.02	−0.11	−0.01	0.16	0.07	0.42	4	F1	**0.59**	0.00	−0.11	−0.04	0.19	0.42
9	F1	**0.53**	−0.2	−0.01	−0.02	0.05	**0.34**	0.44	9	F1	**0.62**	−0.15	−0.10	0.09	0.13	0.40
35	F1	**0.53**	0.21	−0.04	−0.07	0.14	−0.06	0.47	35	F1	**0.51**	0.21	0.00	−0.11	0.12	0.46
26	F1	**0.52**	0.17	−0.17	−0.02	0.08	−0.02	0.36	26	F1	**0.51**	0.17	−0.14	−0.06	0.08	0.36
41	F1	**0.52**	−0.24	0.02	0.12	−0.07	0.18	0.28	41	F1	**0.59**	−0.24	0.03	0.12	−0.03	0.28
**–**	**–**	**–**	**–**	**–**	**–**	**–**	**–**	**–**	6	F1	**0.48**	0.00	−0.04	0.14	−0.03	0.29
34	F2	−0.05	**0.74**	−0.05	−0.03	0.03	0.06	0.51	34	F2	−0.04	**0.74**	−0.10	0.05	0.03	0.51
29	F2	−0.04	**0.63**	−0.02	0.08	0.12	−0.1	0.43	29	F2	−0.08	**0.63**	−0.02	0.06	0.09	0.42
28	F2	0.09	**0.62**	0.02	−0.01	−0.06	0.05	0.46	28	F2	0.10	**0.63**	−0.01	0.04	−0.06	0.46
7	F2	0.11	**0.48**	0.25	0.06	−0.07	−0.02	0.48	7	F2	0.10	**0.49**	0.25	0.06	−0.09	0.48
45	F2	0.14	**0.46**	0.17	0.18	−0.11	−0.12	0.43	45	F2	0.10	**0.45**	0.22	0.10	−0.14	0.41
5	F2	0.01	**0.45**	0.19	0.03	−0.08	0.12	0.36	5	F2	0.06	**0.45**	0.13	0.10	−0.06	0.35
18	F3	−0.14	0.09	**0.81**	−0.06	0.00	0.09	0.63	18	F3	−0.10	0.07	**0.77**	0.04	0.00	0.60
40	F3	0.04	0.09	**0.69**	0.08	−0.05	0.04	0.59	40	F3	0.06	0.06	**0.70**	0.13	−0.07	0.60
16	F3	−0.07	0.03	**0.64**	0.01	0.21	0.04	0.54	16	F3	−0.06	0.02	**0.62**	0.07	0.20	0.53
37	F3	−0.05	−0.05	**0.64**	0.06	0.29	−0.02	0.57	37	F3	−0.07	−0.07	**0.64**	0.09	0.27	0.58
25	F3	0.12	0.26	**0.54**	−0.08	−0.11	0.09	0.50	25	F3	0.16	0.24	**0.50**	0.00	−0.11	0.48
20	F4	−0.05	0.06	−0.03	**0.75**	0.06	0.03	0.63	20	F4	−0.07	0.04	0.00	**0.77**	0.05	0.61
50	F4	0.00	0.05	0.05	**0.71**	−0.06	−0.11	0.47	50	F4	−0.04	0.04	0.12	**0.60**	−0.07	0.37
23	F4	−0.01	0.1	−0.01	**0.38**	0.05	**0.31**	0.45	23	F4	0.08	0.11	−0.08	**0.51**	0.12	0.44
**–**	**–**	**–**	**–**	**–**	**–**	**–**	**–**	**–**	36	F4	−0.05	0.13	0.29	**0.44**	0.18	0.57
**–**	**–**	**–**	**–**	**–**	**–**	**–**	**–**	**–**	32	F4	0.07	0.15	0.00	**0.41**	0.14	0.40
17	F5	−0.09	0.03	−0.03	−0.02	**0.74**	0.09	0.55	17	F5	−0.08	0.01	−0.09	0.09	**0.76**	0.54
13	F5	0.15	−0.16	0.25	0.01	**0.62**	−0.18	0.49	13	F5	0.08	−0.17	**0.31**	−0.07	**0.59**	0.48
38	F5	0.10	−0.14	**0.37**	0.04	**0.61**	−0.23	0.58	38	F5	0.02	−0.16	**0.43**	−0.04	**0.55**	0.55
21	F5	0.07	0.04	−0.2	−0.06	**0.59**	0.17	0.46	21	F5	0.10	0.04	−0.29	0.08	**0.63**	0.47
3	F5	0.19	−0.01	−0.06	0.19	**0.35**	0.11	0.42	3	F5	0.21	0.00	−0.06	0.21	**0.38**	0.41
32	F4	−0.18	0.14	0.23	0.04	−0.02	**0.73**	0.61	**–**	**–**	**–**	**–**	**–**	**–**	**–**	**–**
8	F6	0.04	−0.1	0.21	−0.06	0.03	**0.65**	0.45	**–**	**–**	**–**	**–**	**–**	**–**	**–**	**–**
6	F1	**0.33**	−0.04	0.11	−0.08	−0.14	**0.47**	0.37	**–**	**–**	**–**	**–**	**–**	**–**	**–**	**–**
15	F1	0.10	0.02	−0.2	0.26	0.11	**0.43**	0.51	**–**	**–**	**–**	**–**	**–**	**–**	**–**	**–**

^a^Original factor according to Laurent et al. [7]. Loadings ≥ |0.30| bolded. See supplementary material for the results of all models

However, we did not replicate the original sixth factor of Laurent et al. [7] (see loadings for the six-factor, 43-item model in the supplementary material A). Instead, the six-factor, 31-item model presented here, includes a factor named "lack of support and resources from the organisation", which is measured by four items (loadings ≥ .30; #6 “Shortage of beds in the unit", #15 "Absence of administrative support", #32 "Lack of staff" and #8 "Unsuitable or under-equipped treatment area or defective equipment"). Compared to the five-factor model, the inclusion of this sixth factor, modifies factor 4 somewhat (with an exclusive focus on the issue of schedules, on-call duties and their balance with private life). Item #32 "Lack of staff" now loads on factor 6, and item #36 "Heavy and permanent workload" was removed because it correlated fairly strongly with items #15 and #32, which are more specific to factor 6, while correlating fairly strongly with other factors (4, 3, 5). Items #6 and #15, initially associated with factor 1, have higher loadings on factor 6.

The explained variances are generally equivalent (on average around 0.50), with an improvement for items #6 (Δ*R*^2^ = 0.08) and #32 (Δ*R*^2^ = 0.21) with factor 6 taken into account. A few residual cross-loadings can be noted for items #9, #23, #13, #38, #6, but each time with a higher loading on the expected factor. For both models, the correlations between factors are all significant (*p* <.0001) and positive, with values ranging from .33 to .66, which attests their propensity to reflect the same general construct (i.e., stress) while assessing specific causes of stress in a discriminating manner.

#### Results of item response theory (IRT) methodology

Table 4 summarises the indices obtained from the IRT. Whether for the five-factor, 30-item model or the six-factor, 31-item model, these indices confirm the relevance of the items and the scale. In fact, all the empirical marginal reliability indices are greater than .70, all the RMSEA indices per item were lower than .05 and all the items obtained a discrimination parameter >1 (or very close to it, i.e. 0.997 for item 41). Likewise, for each factor the test information function (TIF) was quite good, even very good, with a range of the latent variable (θ), with an information value >1 and a standard error <1 always greater than [−2; +2]. It should be noted that, logically, when the number of items is higher, the reliability and range indices of the TIF increased and the discrimination parameters had values more homogenously situated between 1 and 2. The reduction in the number of items for factors 1, 4 and 6 of the six-factor model therefore came at a cost, but without unduly reducing the reliability and informativeness of the measure. A few imperfections can be noted in some of the items regarding one or more criteria, but again this does not invalidate their relevance and inclusion in the scale (see supplementary material B). For example, item 41 presents characteristic curve (ICC) and information curve (IIF) that were very different from the other items of the first factor, but since it provided slightly more information about the extreme levels of the variable, it nevertheless contributed to the overall range of the TIF.

**Table 4:** Indices derived from the Item Response Theory (IRT) for the five-factor, 30-item model, and the six-factor, 31-item model for the population of nurses, physicians and residents

Factor model	Indices	Factor 1	Factor 2	Factor 3	Factor 4	Factor 5	Factor 6
Five factors, 30 items	Empirical marginal reliability	.835	.796	.827	.786	.756	**–**
	Discrimination parameter(*a*) [min. value; max. value]	[1.008; 1.797]	[1.326; 1.692]	[1.639; 2.378]	[1.221; 1.898]	[1.194; 1.858]	**–**
	RMSEA per item [min. value; max. value]	[.000; .021]	[.009; .032]	[.001; .028]	[.020; .030]	[.008; .033]	**–**
	TIF: range of the latent variable (θ) with information value >1 and standard error <1	[−4.25 to +4.75]	[−4.55 to +3.55]	[−4.5 to +2.95]	[−3.70 to +3.20]	[−2.80 to +3.20]	**–**
	*N* items	9	6	5	5	5	**–**
Six factors, 31 items	Empirical marginal reliability	.827	Same as five-factor model		.782	Same as five-factor model	.752
	Discrimination parameter (*a*) [min. value; max. value]	[0.997; 1.881]			[1.215; 3.399]		[1.170; 2.390]
	RMSEA per item [min. value; max. value]	[.000; .022]			[.033; .042]		[.010; .030]
	TIF: range of the latent variable (θ) with information value >1 and standard error <1	[−4.10 to +4.60]			[−2.90 to +2.45]		[−2.50 to +2.40]
	*N* items	8			3		4

Evaluation criteria: empirical marginal reliability >.70; Discrimination parameters >0.5, ideally between 1 and 2, if >2 risk of redundancy; Root Mean Square Error of Approximation (RMSEA) per item ideally < .05, otherwise < .08; Test information function (TIF) with the widest possible range, at least [−2; +2]

### Measurement invariance between nurses and physicians/residents (ESEM)

Next, we examined the measurement invariance between nurses on the one hand (*n* = 1118), and physicians and residents together on the other hand (*n* = 473), for the two most satisfactory models selected before (i.e., the six-factor, 31-item, and five-factor, 30-item models, see ESEM results). As Table 5 illustrates, ESEM fit indices reflect the scale’s good invariance, at configural, metric and scalar level. When comparing the two models, it appears that the six-factor, 31-item model obtained slightly better indices than the five-factor, 30-item model.

**Table 5:** Fit indices and variations of those indices (Δ) obtained for the six-factor model with 31 items, and the five-factor model with 30 items, at different levels of invariance for nurses (group 1) and physicians/residents (group 2)

		Level of invariance		
		Configural	Metric	Scalar
Six factors with 31 items	χ^2^ (*df*)	727.45 (588)	1283.50 (738) Δ556.05 (150)	1574.68 (825) Δ291.18 (87)
	χ^2^/*df*	1.237	1.739 Δ0.502	1.909 Δ0.170
	CFI	.968	.973 Δ.005	.960 Δ−0.013
	RMSEA	.045	.037 Δ−.008	.043 Δ.006
	SRMR	.028	.036 Δ.008	.038 Δ.002
Five factors with 30 items	χ^2^ (*df*)	999.38 (600)	1656.04 (720) Δ656.66 (120)	1984.11 (805) Δ328.07 (85)
	χ^2^/*df*	1.666	2.300 Δ0.633	2.465 Δ0.165
	CFI	.961	.964 Δ.003	.948 Δ−0.016
	RMSEA	.050	.043 Δ−.007	.049 Δ.006
	SRMR	.034	.042 Δ.008	.044 Δ.002

χ^2^ = Chi-square, *df* = degrees of freedom, CFI = Comparative Fit Index, RMSEA = Root Mean Square Error of Approximation, SRMR = Standardised Root Mean squared Residual Except for SRMR, reported values are scaled. The Greek letter ‘Delta’ (Δ) is used for representing the dynamics of a statistical coefficient between two models (here between two steps), and corresponds to the difference (−) between the two compared values

### Metrological properties of PS-ICU for nursing auxiliaries

**Fig. 1:**
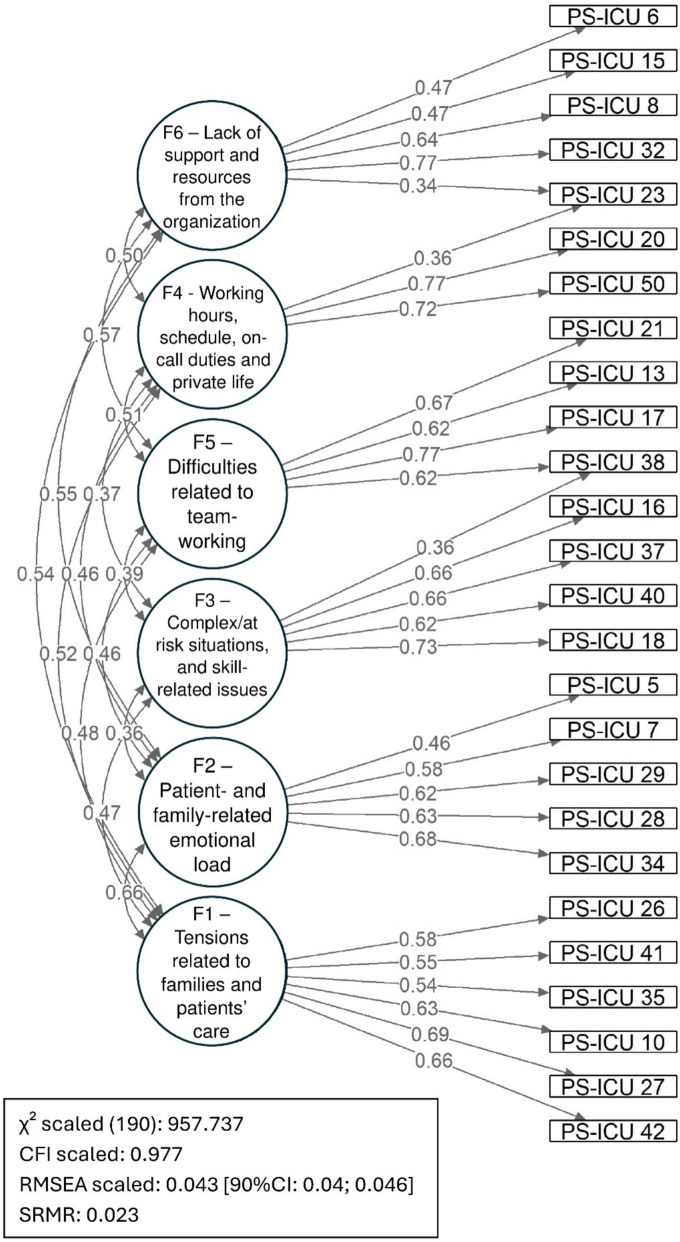
Path diagram of the exploratory structural equation modelling for the shortened 26-item version of the PS-ICU scale for the whole sample (nurses, physicians, residents, and nursing auxiliaries; only loadings ≥ 0.30 are reported)

As the six-factor, 31-item model obtained better indices than all other tested models, we selected it to examine the relevance of PS-ICU for nursing auxiliaries. It produced satisfactory indices for this sample too (*N* = 608), scaled χ^2^(294) = 594.843; χ^2^/*df* = 2.023; CFI scaled = 0.977; RMSEA scaled = 0.041; SRMR = 0.028. Likewise, the internal consistency indices (ω) were satisfactory, although with a slightly lower index for factor 4 (factor 1: .832; factor 2: .762; factor 3: .779; factor 4: .685; factor 5: .796; factor 6: .723). The explained variances of the items were equivalent to those obtained for nurses, physicians and residents (i.e. ranging from .280 to .688, with a *R*^2^ mean of .502). However, when we refer to the factor loadings (see supplementary material A), five items (#4, #5, #9, #45 and #25) appeared with loadings different from the initial PS-ICU population (e.g., cross-loadings, with main loading on another factor). They therefore seem to have to be dismissed so that the six factors can be estimated in an equivalent manner for these different professional groups (including nursing auxiliaries). Overall, the removal of these items has no substantial impact on the ESEM and IRT indices for the two populations (see supplementary materials A and B, model 9’’). It should be noted, however, that for the nursing auxiliary population, item #23 loads less on factor 4 than for the other professionals (ESEM) and has an equivalent loading on factor 6. It should also be noted that the measurement for this factor is less informative, and that RMSEA values are between .05 and .06 for 2 items (IRT). Despite these differences, the configural, metric and scalar invariance indices for these two populations were satisfactory (see supplementary material A). Figure 1 shows the ESEM results obtained for this shortened 26-item version for all the participants surveyed (factor loadings >.30).

### Assessment of general stress and length of the scale

**Fig. 2:**
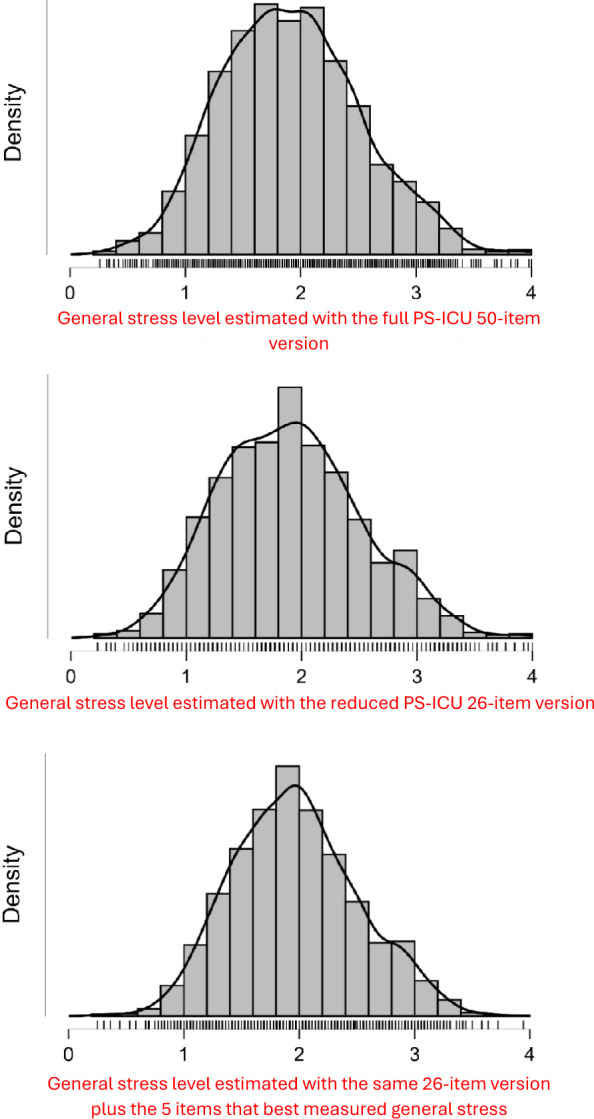
Distributions observed for the full version of PS-ICU (50 items) and the two alternative shortened versions (26 items, and the same 26-item version plus the 5 items that best measured general stress)

The various analyses reported above led to the potential removal of 17 items in addition to the 7 items previously identified as superfluous by Laurent et al. [7]. But does a reduced 26-item version assess general stress as well as the full 50-item scale? Likewise, do any of the potentially removed items assess general stress substantially and should therefore be retained in the scale for this purpose? An analysis of Table 6 and Fig. 2 shows that the 26-item version produced on the entire sample (*N* = 2199) descriptive statistics and distributions very close to those of the 50-item version, whereas the longer version retaining the five items that reflect more general stress (see Method and supplementary material C) does not really offer any added value compared to the shorter 26-item version, which therefore offers the best efficiency/length ratio. This observation is reinforced by the correlations observed, with the 26-item version being very strongly correlated with the 50-item version (*r* = .975), while adding the five items that best measured general stress did not increase the correlation (*r* = .963).

**Table 6:** Descriptive statistics for the evaluation of the general stress level with the full version (50 items) of the PS-ICU and the two alternative shortened versions (26 items, and the same 26-item version plus the 5 items that best measured general stress)

Statistics	General stress level estimated with...		
	Full PS-ICU 50-item version	Shortened PS-ICU 26-item version following previous analyses	Shortened PS-ICU 26-item + the 5 items that best measured general stress
Minimum	0.260	0.231	0.250
Maximum	4.000	4.000	3.944
Mean	1.920	1.905	1.967
Standard Deviation	0.600	0.613	0.544
Skewness	0.259	0.275	0.232
Kurtosis	−0.124	−0.198	−0.150
25th percentile (Q1)	1.480	1.462	1.583
50th percentile (Q2, Median)	1.880	1.885	1.944
75th percentile (Q3)	2.320	2.308	2.306
Interquartile range (Q3–Q1)	0.840	0.846	0.722

## Discussion

### Contributions of the study

**Fig. 3:**
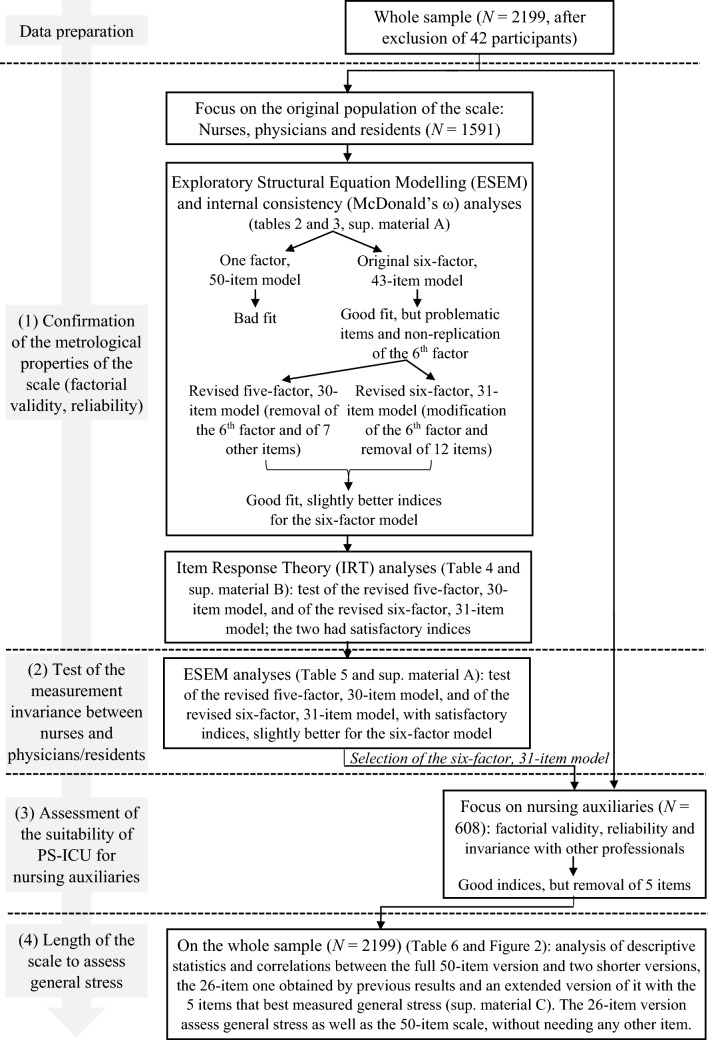
Summary of the data analysis strategy and results associated with the different aims

This study pursued three aims: 1) to confirm the metrological properties of the PS-ICU scale on a large sample of ICU nurses, physicians and residents, 2) to test its measurement invariance across these occupational groups and 3) to examine whether the scale would be also suited for nursing auxiliaries. Moreover, to increase its usability for research and preventive purposes, its reduction was also of interest, via the removal of potentially problematic items. Accordingly, on data collected on more than 2000 professionals from 42 units, we applied an iterative data analysis strategy, with many results, which are summarized in the Fig. 3.

We first overall confirmed the metrological properties of the PS-ICU (factorial validity, reliability) on its original population, while revising its measurement of stress and stressors with a reduced number of items. More specifically, the ESEM, IRT and internal consistency (McDonald's Omega) analyses largely confirmed the relevance of the first five factors observed by the previous studies [7, 10, 11]. The sixth original factor, however, was not replicated, but was observed in another form entitled "lack of support and resources from the organisation". Conserving this sixth factor slightly increase model fit (with a 31-item version) in comparison to a five-factor model (with a 30-item version).

Next, ESEM results confirmed the invariance of the PS-ICU between nurses and physicians/residents, for the two compared shortened revised versions (i.e., the five-factor, 30-item, and six-factor, 31-item ones), and at the three levels (i.e., configural, metric, and scalar) required for making valid group comparisons on the latent measured variables [15]. Again, we observed slightly better fit invariance indices for the six-factor, 31-item version of the scale. As a result, we selected this latter version of the scale for the remaining analyses. In fact, this factor 6 is highly relevant to future studies on stress in intensive care, as recent studies illustrated the importance of adequate human and material resources, as well as organisational support, in order to combat burnout and moral distress, but also to promote quality and safety of care [28–32].

In a third series of analyses using this six-factor, 31-item version of PS-ICU, we obtained results that overall support its use for nursing auxiliaries, conditional to the removal of five other items which were non-invariant with other occupational groups. This means that a further reduced 26-item version is preferable as it can be used, particularly in France, in a more appropriate way by all intensive care team staff. All the studies on stress and burnout focus mainly on nurses and physicians. This 26-item PS-ICU will make it possible to include this population in future studies and improve our knowledge of its specific characteristics.

Finally, given that the various successive analyses ended in a 26-item reduced version being psychometrically sound and suitable for use with physicians, residents, nurses and nursing auxiliaries, the PS-ICU scale has now met the challenge of being simpler and faster to use, both in connection with research and in routine use in units. Many authors agree in saying that s**y**stematically identifying stress and sources of stress within teams would make it possible to intervene more effectively with healthcare staff and prevent the consequences, such as professional burnout [6, 33, 34]. For this purpose, shorter tools are more suited than longer ones. It is worth noting that this 26-item version was as well effective in assessing general stress level than the full 50-item scale, without needing additional items (as adding the five items that best measured general stress neither increased the similarity of descriptive statistics nor the correlation of the shorter scale with the complete scale). Since the PS-ICU is based on an international development [7, 11], readers can get it in several languages (i.e., French, Italian, Spanish, English and Chinese) in the supplementary material D.

### Limitations and perspectives for future studies

This study presents some limitations. Firstly, despite our efforts to include units and participants (with an average voluntary participation rate of 58.10%), the number of physicians and residents was insufficient to distinguish between them in the invariance analyses. Given the existence of studies showing variations in the causes of stress in these two groups [35–37], it therefore remains possible that certain minor differences in the measurement of stress for these two groups could not be identified. Future studies will therefore have to analyse these possible differences in measurement. Another limitation is that the total number of physicians and residents was not sufficient to estimate strict invariance (i.e., also including the equivalence of residual variances). However, this is not a major limitation, as there is no consensus as to whether it is necessary [38–41] and many researchers consider it to be optional. Regarding sampling we were able to compare participants and non-participants about their occupations only, which is another limitation of the study. At this level, it seemed that our study motivated less the nursing auxiliaries to participate than other professionals (especially physicians). Exploring specifically nursing auxiliaries’ attitudes, social behaviours and specific burden within ICUs might be useful to provide explanations to this lower participation rate. The final version of the scale (26 items and 6 factors) also has a minor weakness in factor 4 "working hours, schedule, on-call duties and private life". In fact, this factor has only three items and item #23 has a moderate loading (between .30 and .40) while also being associated with factor 6. In addition, this factor 4 seems to be slightly less accurately estimated for nursing auxiliaries. This therefore represents a possible future improvement to the scale, for example by adding an item and/or slightly rewording item #23. This limitation of the scale remains minor given the very favourable indices obtained from the various analyses (ESEM, IRT, McDonald's Omega). Finally, because this study was only conducted in France using the French-language version of the scale, the collected answers may be not comparable with those that could be collected in other contexts. Consequently, it is necessary to replicate the results obtained in other organisational, cultural, economic, societal and linguistic contexts.

## Conclusion

The PS-ICU scale, which measures generic, intensive care-specific stress factors, is a valid and reliable scale that can be used with nurses, physicians and residents, as well as nursing auxiliaries. In 26 items, it allows researchers and supervisors of intensive care units to propose it, in order to evaluate the extent and type of stress factors perceived by healthcare staff within the unit.

## Supplementary Information

Additional file 1.

Additional file 2.

Additional file 3.

Additional file 4.

## Data Availability

The datasets used and/or analysed during the current study are available from the corresponding author on reasonable request.

## Abbreviations

ICUs: Intensive Care Units
PS-ICU: Perceived Stressors in Intensive Care Units
ESEM: Exploratory Structural Equation Modelling
IRT: Item Response Theory
RMSEA: Root Mean Square Error of Approximation
CFI: Comparative Fit Index
SRMR: Standardized Root Mean Square Residual
AIC: Akaike Information Criterion
TIF: test information function
ICC: item characteristic curve
IIF: item information curve
