# Supplementary material for: Further analysis and refinements of the perceived stressors in intensive care units (PS-ICU) scale: a French nation-wide cross-sectional multicentre study
Source: Ann Intensive Care. 2025 Nov 20;15:186. doi: 10.1186/s13613-025-01572-7 (PMC12630482; doi:10.1186/s13613-025-01572-7)
Supplement: Supplementary file 2 — Additional file 2. [file 13613_2025_1572_MOESM2_ESM.docx]

Summary

[1 Five-factor model with 30 items (model #7) estimated on the original population of the scale (nurses, physicians and medical residents) 1](#_Toc197780876)

[1.1 IRT for the entire scale (30 items) 1](#_Toc197780877)

[1.1.1 IRT model fitting and comparison 1](#_Toc197780878)

[1.1.2 Evaluation of the selected model 1](#_Toc197780879)

[1.1.3 Charts of item characteristics 4](#_Toc197780880)

[1.1.4 Model and item reliability 12](#_Toc197780881)

[1.2 IRT for factor 1 of the 5-factor model 12](#_Toc197780882)

[1.2.1 IRT model fitting and comparison 12](#_Toc197780883)

[1.2.2 Evaluation of the selected model 12](#_Toc197780884)

[1.2.3 Charts of item characteristics 13](#_Toc197780885)

[1.2.4 Model and item reliability 16](#_Toc197780886)

[1.3 IRT for factor 2 of the 5-factor model 16](#_Toc197780887)

[1.3.1 IRT model fitting and comparison 16](#_Toc197780888)

[1.3.2 Evaluation of the selected model 17](#_Toc197780889)

[1.3.3 Charts of item characteristics 18](#_Toc197780890)

[1.3.4 Model and item reliability 20](#_Toc197780891)

[1.4 IRT for factor 3 of the 5-factor model 20](#_Toc197780892)

[1.4.1 IRT model fitting and comparison 20](#_Toc197780893)

[1.4.2 Evaluation of the selected model 20](#_Toc197780894)

[1.4.3 Charts of item characteristics 21](#_Toc197780895)

[1.4.4 Model and item reliability 23](#_Toc197780896)

[1.5 IRT for factor 4 of the 5-factor model 23](#_Toc197780897)

[1.5.1 IRT model fitting and comparison 23](#_Toc197780898)

[1.5.2 Evaluation of the selected model 23](#_Toc197780899)

[1.5.3 Charts of item characteristics 24](#_Toc197780900)

[1.5.4 Model and item reliability 26](#_Toc197780901)

[1.6 IRT for factor 5 of the 5-factor model 26](#_Toc197780902)

[1.6.1 IRT model fitting and comparison 26](#_Toc197780903)

[1.6.2 Evaluation of the selected model 26](#_Toc197780904)

[1.6.3 Charts of item characteristics 27](#_Toc197780905)

[1.6.4 Model and item reliability 29](#_Toc197780906)

[2 Six-factor model with 31 items (model #9) estimated on the original population of the scale (nurses, physicians and medical residents) 29](#_Toc197780907)

[2.1 IRT for the entire scale (31 items) 29](#_Toc197780908)

[2.1.1 IRT model fitting and comparison 29](#_Toc197780909)

[2.1.2 Evaluation of the selected model 29](#_Toc197780910)

[2.1.3 Charts of item characteristics 32](#_Toc197780911)

[2.1.4 Model and item reliability 40](#_Toc197780912)

[2.2 IRT for factor 1 of the 6-factor model 40](#_Toc197780913)

[2.2.1 IRT model fitting and comparison 40](#_Toc197780914)

[2.2.2 Evaluation of the selected model 41](#_Toc197780915)

[2.2.3 Charts of item characteristics 42](#_Toc197780916)

[2.2.4 Model and item reliability 44](#_Toc197780917)

[2.3 IRT for factor 2 of the 6-factor model 44](#_Toc197780918)

[2.3.1 IRT model fitting and comparison 44](#_Toc197780919)

[2.3.2 Evaluation of the selected model 45](#_Toc197780920)

[2.3.3 Charts of item characteristics 46](#_Toc197780921)

[2.3.4 Model and item reliability 48](#_Toc197780922)

[2.4 IRT for factor 3 of the 6-factor model 48](#_Toc197780923)

[2.4.1 IRT model fitting and comparison 48](#_Toc197780924)

[2.4.2 Evaluation of the selected model 48](#_Toc197780925)

[2.4.3 Charts of item characteristics 49](#_Toc197780926)

[2.4.4 Model and item reliability 51](#_Toc197780927)

[2.5 IRT for factor 4 of the 6-factor model 51](#_Toc197780928)

[2.5.1 IRT model fitting and comparison 51](#_Toc197780929)

[2.5.2 Evaluation of the selected model 51](#_Toc197780930)

[2.5.3 Charts of item characteristics 52](#_Toc197780931)

[2.5.4 Model and item reliability 53](#_Toc197780932)

[2.6 IRT for factor 5 of the 6-factor model 53](#_Toc197780933)

[2.6.1 IRT model fitting and comparison 53](#_Toc197780934)

[2.6.2 Evaluation of the selected model 54](#_Toc197780935)

[2.6.3 Charts of item characteristics 55](#_Toc197780936)

[2.6.4 Model and item reliability 57](#_Toc197780937)

[2.7 IRT for factor 6 of the 6-factor model 57](#_Toc197780938)

[2.7.1 IRT model fitting and comparison 57](#_Toc197780939)

[2.7.2 Evaluation of the selected model 57](#_Toc197780940)

[2.7.3 Charts of item characteristics 58](#_Toc197780941)

[2.7.4 Model and item reliability 59](#_Toc197780942)

[3 Six-factor model with 31 items (model #9) estimated on the sample of nursing auxiliaries 59](#_Toc197780943)

[3.1 IRT for the entire scale (31 items) 59](#_Toc197780944)

[3.1.1 IRT model fitting and comparison 59](#_Toc197780945)

[3.1.2 Evaluation of the selected model 60](#_Toc197780946)

[3.1.3 Charts of item characteristics 62](#_Toc197780947)

[3.1.4 Model and item reliability 70](#_Toc197780948)

[3.2 IRT for factor 1 of the 6-factor model 70](#_Toc197780949)

[3.2.1 IRT model fitting and comparison 70](#_Toc197780950)

[3.2.2 Evaluation of the selected model 71](#_Toc197780951)

[3.2.3 Charts of item characteristics 72](#_Toc197780952)

[3.2.4 Model and item reliability 74](#_Toc197780953)

[3.3 IRT for factor 2 of the 6-factor model 74](#_Toc197780954)

[3.3.1 IRT model fitting and comparison 74](#_Toc197780955)

[3.3.2 Evaluation of the selected model 75](#_Toc197780956)

[3.3.3 Charts of item characteristics 76](#_Toc197780957)

[3.3.4 Model and item reliability 78](#_Toc197780958)

[3.4 IRT for factor 3 of the 6-factor model 78](#_Toc197780959)

[3.4.1 IRT model fitting and comparison 78](#_Toc197780960)

[3.4.2 Evaluation of the selected model 78](#_Toc197780961)

[3.4.3 Charts of item characteristics 79](#_Toc197780962)

[3.4.4 Model and item reliability 81](#_Toc197780963)

[3.5 IRT for factor 4 of the 6-factor model 81](#_Toc197780964)

[3.5.1 IRT model fitting and comparison 81](#_Toc197780965)

[3.5.2 Evaluation of the selected model 81](#_Toc197780966)

[3.5.3 Charts of item characteristics 82](#_Toc197780967)

[3.5.4 Model and item reliability 83](#_Toc197780968)

[3.6 IRT for factor 5 of the 6-factor model 83](#_Toc197780969)

[3.6.1 IRT model fitting and comparison 83](#_Toc197780970)

[3.6.2 Evaluation of the selected model 84](#_Toc197780971)

[3.6.3 Charts of item characteristics 85](#_Toc197780972)

[3.6.4 Model and item reliability 87](#_Toc197780973)

[3.7 IRT for factor 6 of the 6-factor model 87](#_Toc197780974)

[3.7.1 IRT model fitting and comparison 87](#_Toc197780975)

[3.7.2 Evaluation of the selected model 87](#_Toc197780976)

[3.7.3 Charts of item characteristics 88](#_Toc197780977)

[3.7.4 Model and item reliability 89](#_Toc197780978)

[4 Six-factor model with 26 items (model #11) estimated on the original population of the scale (nurses, physicians and medical residents) 89](#_Toc197780979)

[4.1 IRT for the entire scale (26 items) 89](#_Toc197780980)

[4.1.1 IRT model fitting and comparison 89](#_Toc197780981)

[4.1.2 Evaluation of the selected model 90](#_Toc197780982)

[4.1.3 Charts of item characteristics 92](#_Toc197780983)

[4.1.4 Model and item reliability 99](#_Toc197780984)

[4.2 IRT for factor 1 of the 6-factor model 99](#_Toc197780985)

[4.2.1 IRT model fitting and comparison 99](#_Toc197780986)

[4.2.2 Evaluation of the selected model 99](#_Toc197780987)

[4.2.3 Charts of item characteristics 100](#_Toc197780988)

[4.2.4 Model and item reliability 103](#_Toc197780989)

[4.3 IRT for factor 2 of the 6-factor model 103](#_Toc197780990)

[4.3.1 IRT model fitting and comparison 103](#_Toc197780991)

[4.3.2 Evaluation of the selected model 103](#_Toc197780992)

[4.3.3 Charts of item characteristics 104](#_Toc197780993)

[4.3.4 Model and item reliability 106](#_Toc197780994)

[4.4 IRT for factor 3 of the 6-factor model 106](#_Toc197780995)

[4.4.1 IRT model fitting and comparison 106](#_Toc197780996)

[4.4.2 Evaluation of the selected model 107](#_Toc197780997)

[4.4.3 Charts of item characteristics 108](#_Toc197780998)

[4.4.4 Model and item reliability 110](#_Toc197780999)

[4.5 IRT for factor 4 of the 6-factor model 110](#_Toc197781000)

[4.5.1 IRT model fitting and comparison 110](#_Toc197781001)

[4.5.2 Evaluation of the selected model 110](#_Toc197781002)

[4.5.3 Charts of item characteristics 111](#_Toc197781003)

[4.5.4 Model and item reliability 112](#_Toc197781004)

[4.6 IRT for factor 5 of the 6-factor model 112](#_Toc197781005)

[4.6.1 IRT model fitting and comparison 112](#_Toc197781006)

[4.6.2 Evaluation of the selected model 113](#_Toc197781007)

[4.6.3 Charts of item characteristics 114](#_Toc197781008)

[4.6.4 Model and item reliability 116](#_Toc197781009)

[4.7 IRT for factor 6 of the 6-factor model 116](#_Toc197781010)

[4.7.1 IRT model fitting and comparison 116](#_Toc197781011)

[4.7.2 Evaluation of the selected model 116](#_Toc197781012)

[4.7.3 Charts of item characteristics 117](#_Toc197781013)

[4.7.4 Model and item reliability 118](#_Toc197781014)

[5 Six-factor model with 26 items (model #11) estimated on the sample of nursing auxiliaries 118](#_Toc197781015)

[5.1 IRT for the entire scale (26 items) 118](#_Toc197781016)

[5.1.1 IRT model fitting and comparison 118](#_Toc197781017)

[5.1.2 Evaluation of the selected model 119](#_Toc197781018)

[5.1.3 Charts of item characteristics 121](#_Toc197781019)

[5.1.4 Model and item reliability 128](#_Toc197781020)

[5.2 IRT for factor 1 of the 6-factor model 128](#_Toc197781021)

[5.2.1 IRT model fitting and comparison 128](#_Toc197781022)

[5.2.2 Evaluation of the selected model 128](#_Toc197781023)

[5.2.3 Charts of item characteristics 129](#_Toc197781024)

[5.2.4 Model and item reliability 132](#_Toc197781025)

[5.3 IRT for factor 2 of the 6-factor model 132](#_Toc197781026)

[5.3.1 IRT model fitting and comparison 132](#_Toc197781027)

[5.3.2 Evaluation of the selected model 132](#_Toc197781028)

[5.3.3 Charts of item characteristics 133](#_Toc197781029)

[5.3.4 Model and item reliability 135](#_Toc197781030)

[5.4 IRT for factor 3 of the 6-factor model 135](#_Toc197781031)

[5.4.1 IRT model fitting and comparison 135](#_Toc197781032)

[5.4.2 Evaluation of the selected model 136](#_Toc197781033)

[5.4.3 Charts of item characteristics 137](#_Toc197781034)

[5.4.4 Model and item reliability 139](#_Toc197781035)

[5.5 IRT for factor 4 of the 6-factor model 139](#_Toc197781036)

[5.5.1 IRT model fitting and comparison 139](#_Toc197781037)

[5.5.2 Evaluation of the selected model 139](#_Toc197781038)

[5.5.3 Charts of item characteristics 140](#_Toc197781039)

[5.5.4 Model and item reliability 141](#_Toc197781040)

[5.6 IRT for factor 5 of the 6-factor model 141](#_Toc197781041)

[5.6.1 IRT model fitting and comparison 141](#_Toc197781042)

[5.6.2 Evaluation of the selected model 142](#_Toc197781043)

[5.6.3 Charts of item characteristics 143](#_Toc197781044)

[5.6.4 Model and item reliability 145](#_Toc197781045)

[5.7 IRT for factor 6 of the 6-factor model 145](#_Toc197781046)

[5.7.1 IRT model fitting and comparison 145](#_Toc197781047)

[5.7.2 Evaluation of the selected model 145](#_Toc197781048)

[5.7.3 Charts of item characteristics 146](#_Toc197781049)

[5.7.4 Model and item reliability 147](#_Toc197781050)

# 1 Five-factor model with 30 items (model #7) estimated on the original population of the scale (nurses, physicians and medical residents)

## 1.1 IRT for the entire scale (30 items)

### 1.1.1 IRT model fitting and comparison

##
##
## == IRT MODEL COMPARISON ==

|  | Modèle | G2 | AIC | BIC | LogLik |
| --- | --- | --- | --- | --- | --- |
| PCM | PCM | 101267.5 | 124967.6 | 125617.6 | -62362.77 |
| Graded | Graded | 100215.8 | 123973.9 | 124779.7 | -61836.94 |
| RSM | RSM | 107322.5 | 130848.6 | 131031.2 | -65390.30 |
| GPCM | GPCM | 100700.5 | 124458.6 | 125264.4 | -62079.31 |

### 1.1.2 Evaluation of the selected model

##
##
## == ITEM PARAMETERS ( Graded ) ==

|  | a | b1 | b2 | b3 | b4 |
| --- | --- | --- | --- | --- | --- |
| PSICU 4 | 1.123 | -2.104 | -1.429 | 0.295 | 2.208 |
| PSICU 6 | 0.902 | -2.344 | -0.249 | 1.301 | 2.970 |
| PSICU 9 | 1.019 | -2.170 | -0.736 | 1.195 | 3.116 |
| PSICU 10 | 1.222 | -2.152 | -0.882 | 0.895 | 2.752 |
| PSICU 26 | 0.995 | -1.975 | 0.397 | 2.130 | 3.633 |
| PSICU 27 | 1.487 | -2.859 | -0.476 | 1.301 | 2.923 |
| PSICU 35 | 1.382 | -1.481 | -0.649 | 0.779 | 2.272 |
| PSICU 41 | 0.787 | -4.901 | 2.041 | 4.048 | 5.715 |
| PSICU 42 | 1.301 | -3.196 | -0.176 | 1.867 | 3.482 |
| PSICU 5 | 1.149 | -3.136 | -1.744 | -0.056 | 1.533 |
| PSICU 7 | 1.464 | -4.740 | -1.611 | 0.205 | 1.825 |
| PSICU 28 | 1.276 | -2.239 | -0.296 | 1.078 | 2.585 |
| PSICU 29 | 1.118 | -1.379 | -0.668 | 0.742 | 2.092 |
| PSICU 34 | 1.116 | -0.950 | -0.108 | 1.256 | 2.700 |
| PSICU 45 | 1.290 | -2.912 | -0.975 | 0.783 | 2.342 |
| PSICU 16 | 1.242 | -3.106 | -2.198 | -0.477 | 0.905 |
| PSICU 18 | 1.068 | -5.211 | -1.547 | 0.490 | 2.146 |
| PSICU 25 | 1.408 | -3.338 | -1.451 | 0.177 | 1.798 |
| PSICU 37 | 1.213 | -2.223 | -1.683 | -0.121 | 1.431 |
| PSICU 40 | 1.393 | -4.406 | -0.932 | 0.760 | 2.271 |
| PSICU 20 | 1.151 | -2.465 | -0.903 | 0.477 | 1.767 |
| PSICU 23 | 1.160 | -2.530 | -0.120 | 1.434 | 2.694 |
| PSICU 32 | 1.239 | -1.899 | -0.906 | 0.470 | 1.778 |
| PSICU 36 | 1.733 | -2.510 | -1.435 | -0.062 | 1.184 |
| PSICU 50 | 0.948 | -3.644 | -0.598 | 1.014 | 2.261 |
| PSICU 3 | 1.182 | -2.521 | -1.053 | 0.352 | 1.821 |
| PSICU 13 | 1.005 | -1.349 | -0.564 | 0.965 | 2.384 |
| PSICU 17 | 0.913 | -1.715 | -0.518 | 0.811 | 2.220 |
| PSICU 21 | 0.763 | -0.871 | -0.160 | 1.534 | 3.187 |
| PSICU 38 | 1.128 | -1.867 | -0.981 | 0.392 | 1.717 |

##
##
## == ASSESSMENT OF MODEL FIT ==

| item | S_X2 | df.S_X2 | RMSEA.S_X2 | p.S_X2 |
| --- | --- | --- | --- | --- |
| PSICU 4 | 237.326 | 202 | 0.010 | 0.045 |
| PSICU 6 | 126.352 | 141 | 0.000 | 0.806 |
| PSICU 9 | 237.302 | 213 | 0.008 | 0.122 |
| PSICU 10 | 255.110 | 188 | 0.015 | 0.001 |
| PSICU 26 | 235.648 | 187 | 0.013 | 0.009 |
| PSICU 27 | 135.226 | 146 | 0.000 | 0.728 |
| PSICU 35 | 238.303 | 193 | 0.012 | 0.015 |
| PSICU 41 | 137.275 | 119 | 0.010 | 0.121 |
| PSICU 42 | 116.808 | 131 | 0.000 | 0.808 |
| PSICU 5 | 195.586 | 193 | 0.003 | 0.434 |
| PSICU 7 | 152.537 | 147 | 0.005 | 0.360 |
| PSICU 28 | 112.979 | 90 | 0.013 | 0.051 |
| PSICU 29 | 259.915 | 213 | 0.012 | 0.016 |
| PSICU 34 | 271.268 | 206 | 0.014 | 0.002 |
| PSICU 45 | 202.735 | 185 | 0.008 | 0.177 |
| PSICU 16 | 153.128 | 183 | 0.000 | 0.947 |
| PSICU 18 | 197.871 | 170 | 0.010 | 0.071 |
| PSICU 25 | 197.867 | 165 | 0.011 | 0.041 |
| PSICU 37 | 232.349 | 200 | 0.010 | 0.058 |
| PSICU 40 | 150.989 | 153 | 0.000 | 0.531 |
| PSICU 20 | 223.838 | 206 | 0.007 | 0.187 |
| PSICU 23 | 223.662 | 198 | 0.009 | 0.102 |
| PSICU 32 | 221.934 | 199 | 0.009 | 0.127 |
| PSICU 36 | 163.639 | 159 | 0.004 | 0.384 |
| PSICU 50 | 223.554 | 205 | 0.008 | 0.178 |
| PSICU 3 | 225.155 | 203 | 0.008 | 0.137 |
| PSICU 13 | 214.349 | 220 | 0.000 | 0.595 |
| PSICU 17 | 254.081 | 230 | 0.008 | 0.132 |
| PSICU 21 | 294.959 | 225 | 0.014 | 0.001 |
| PSICU 38 | 233.896 | 213 | 0.008 | 0.155 |

### 1.1.3 Charts of item characteristics


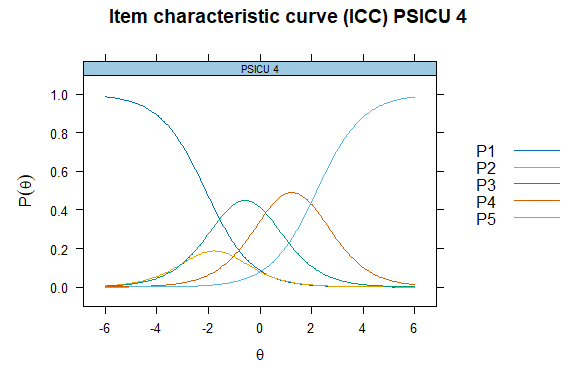

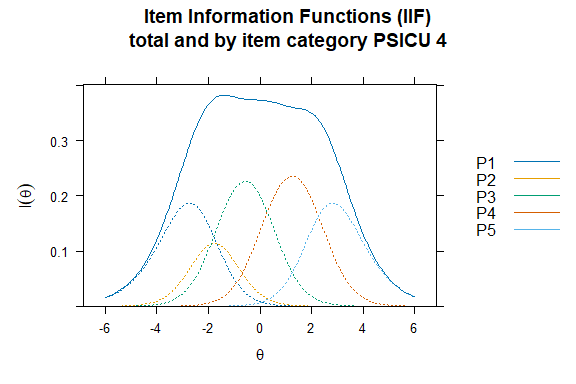

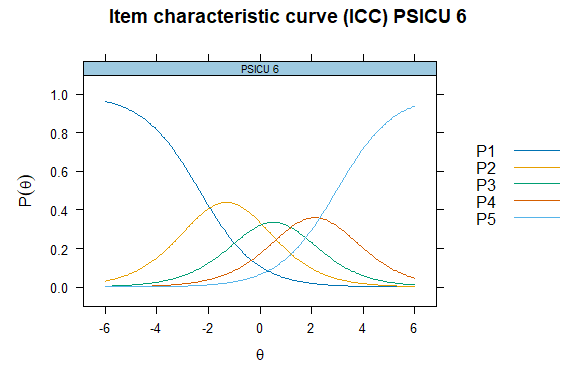

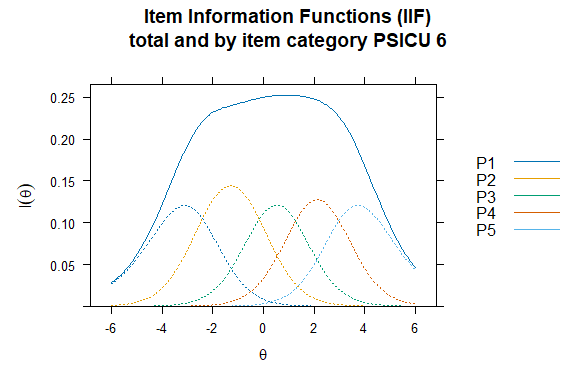

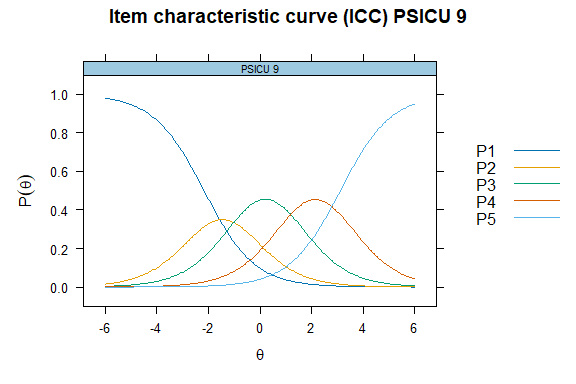

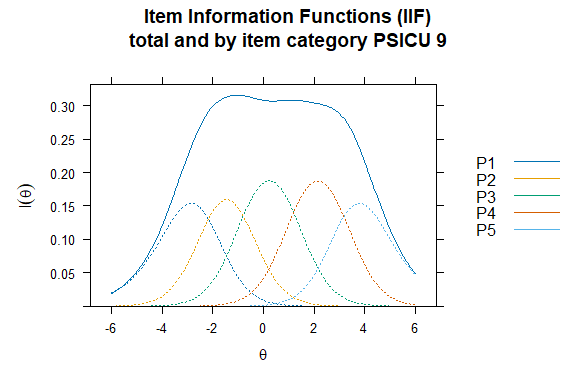

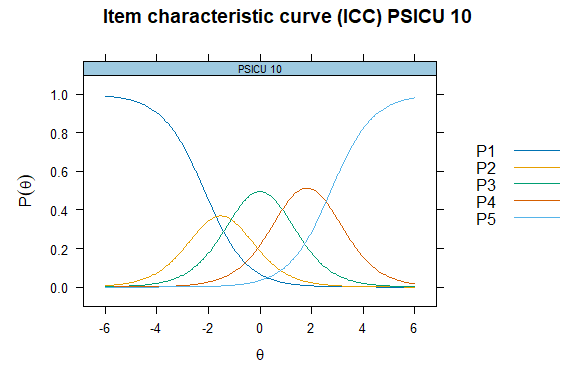

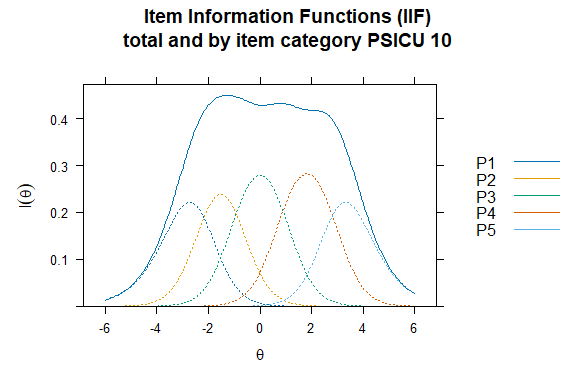

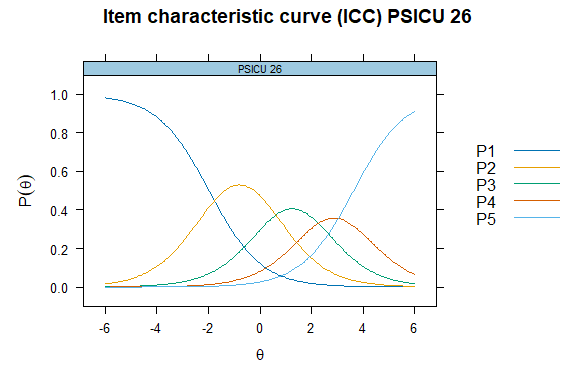

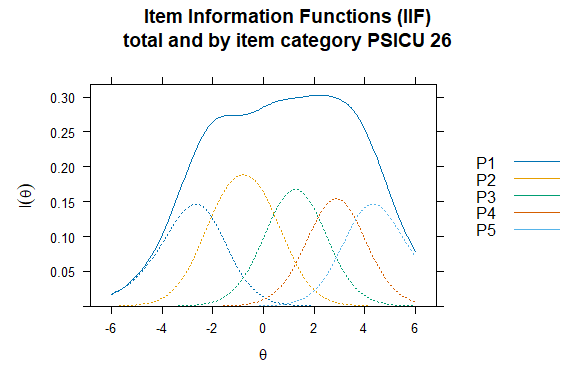

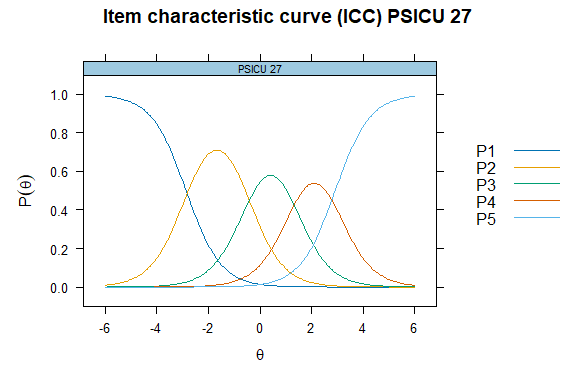

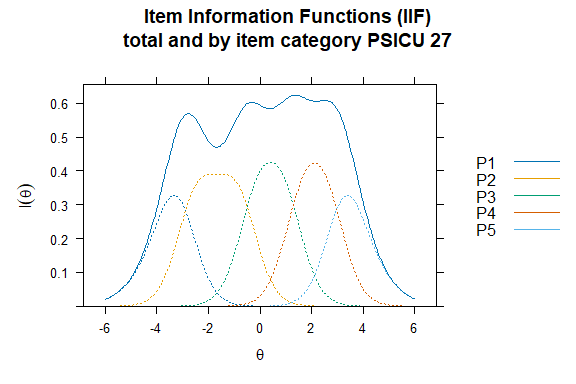

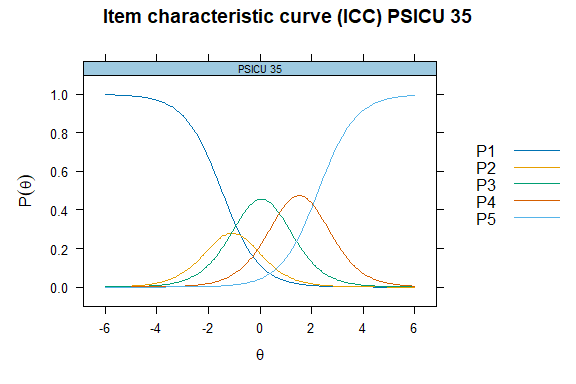

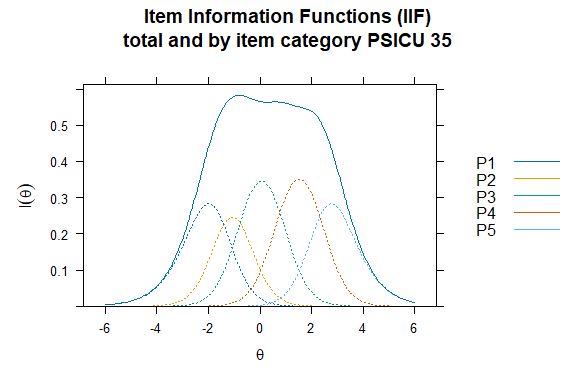

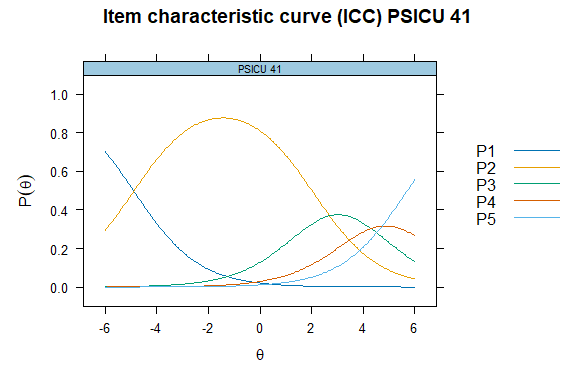

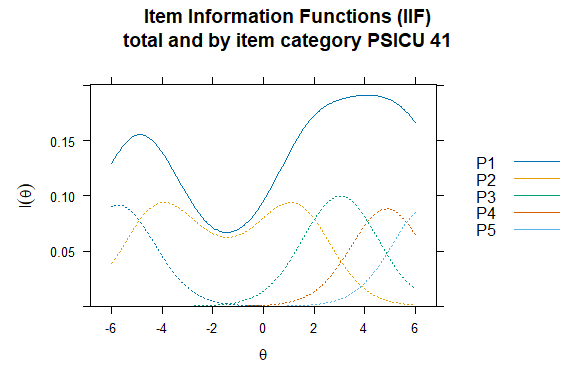

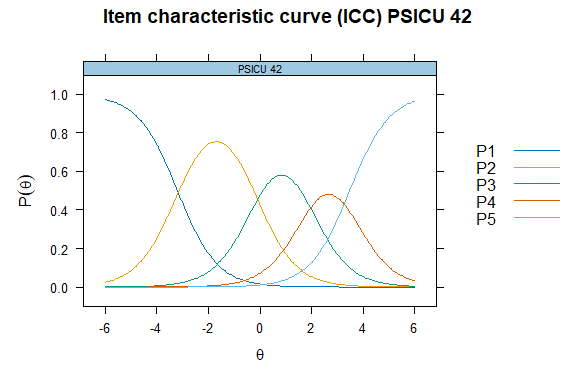

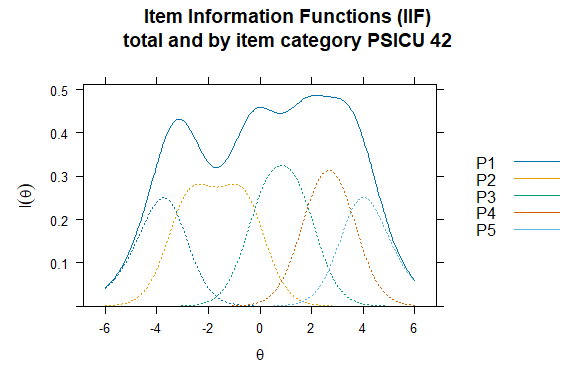

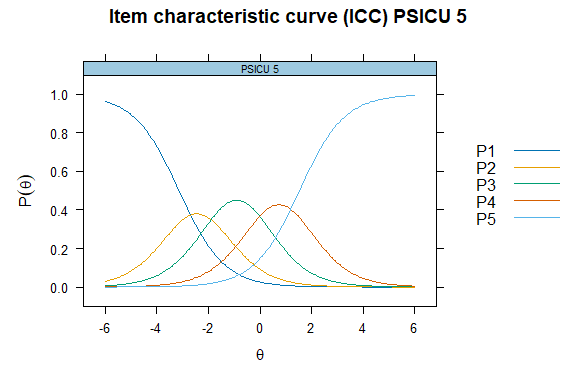

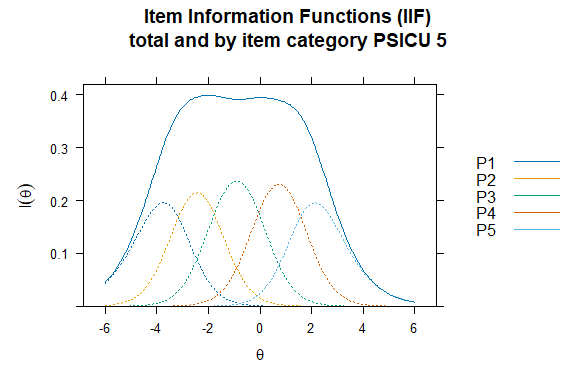

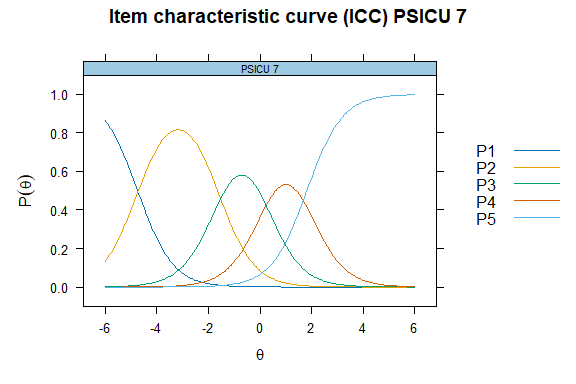

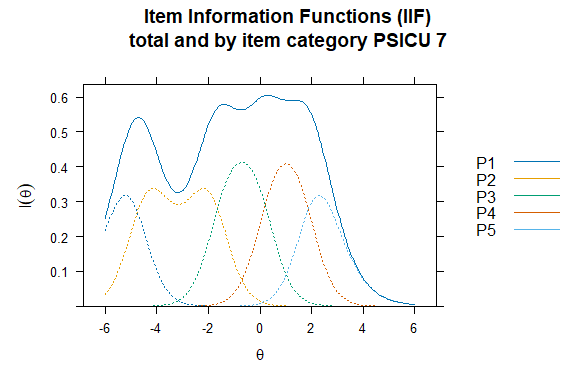

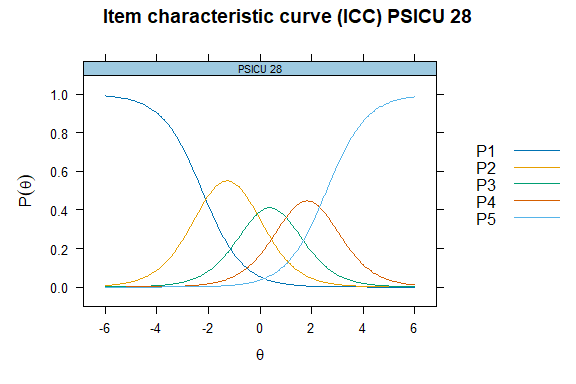

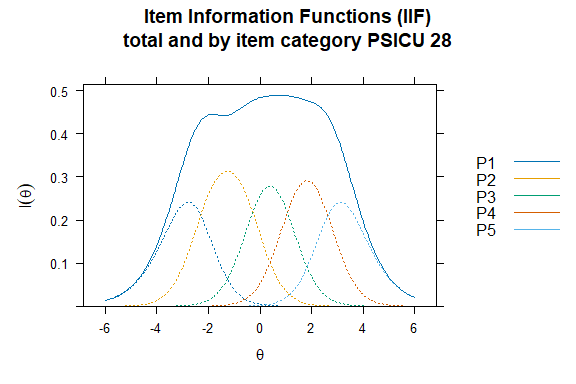

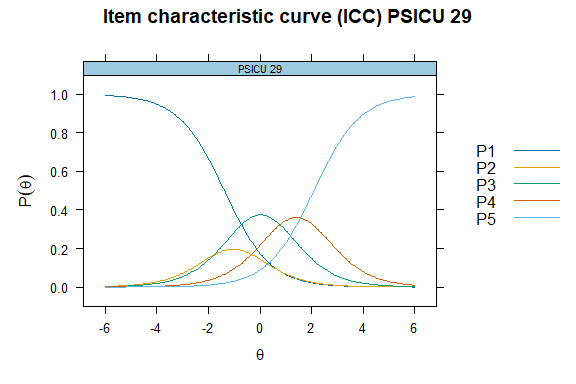

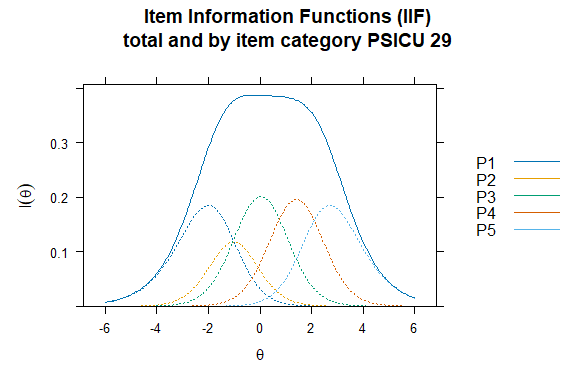

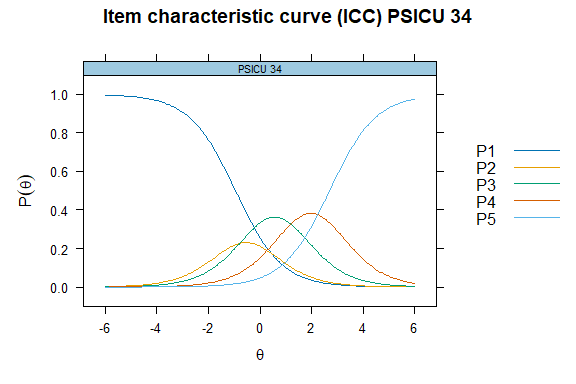

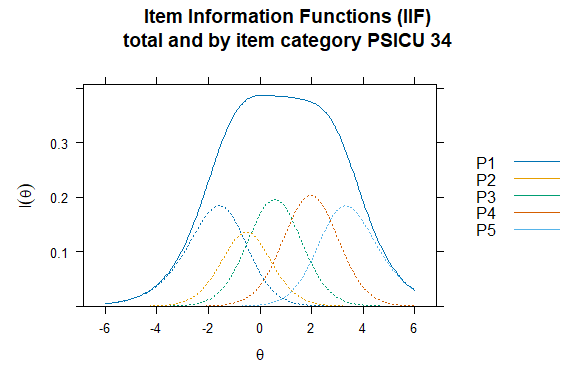

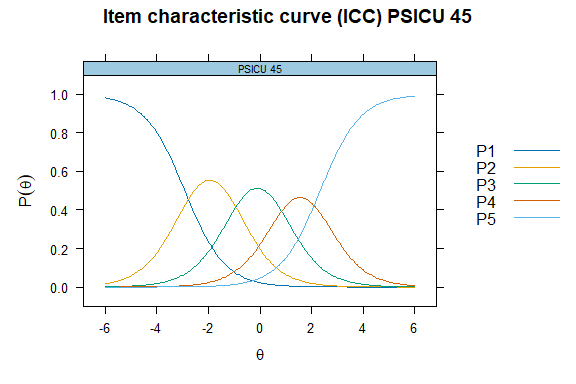

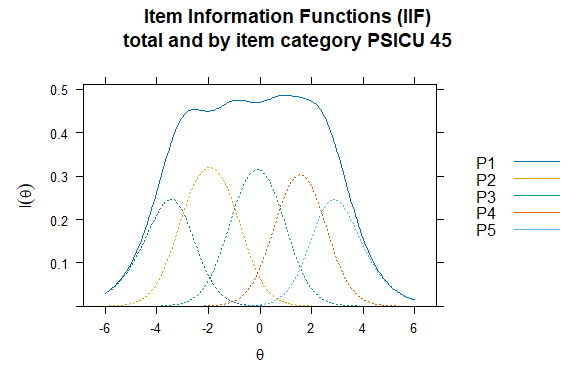

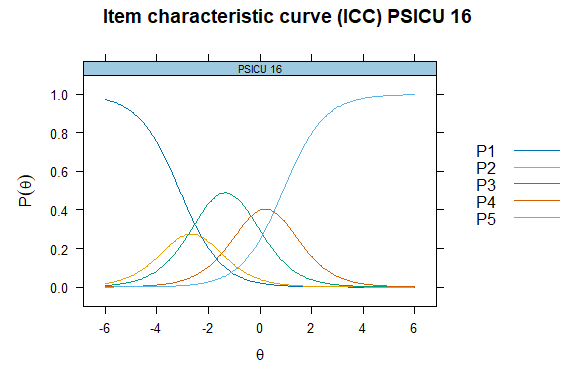

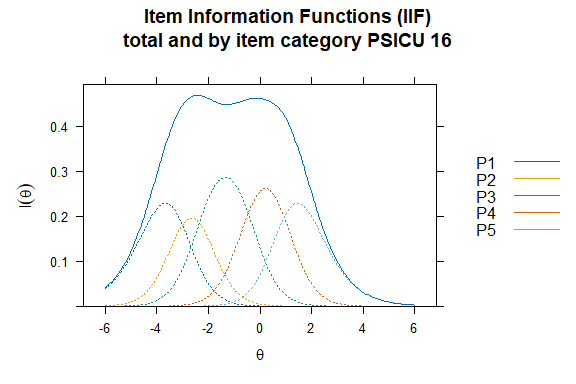

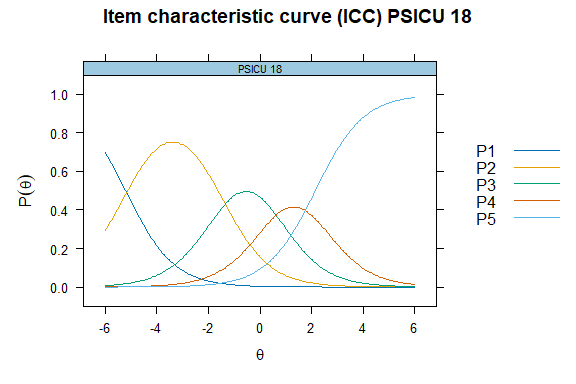

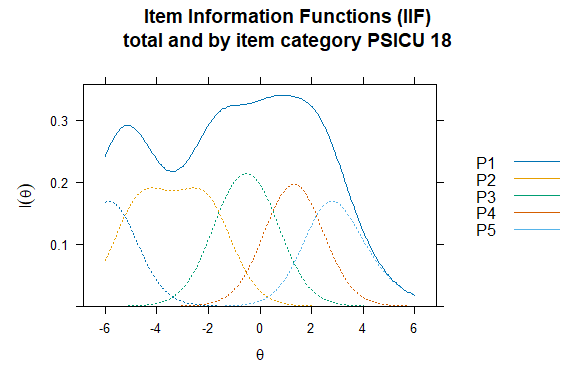

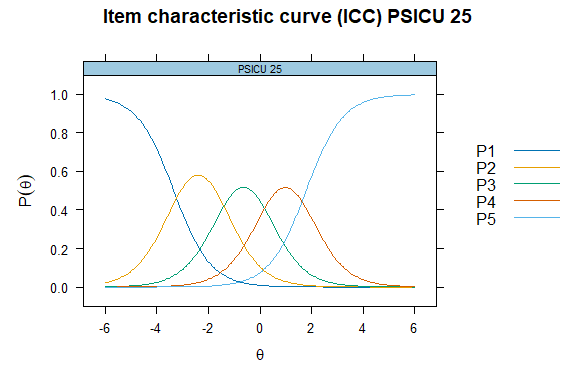

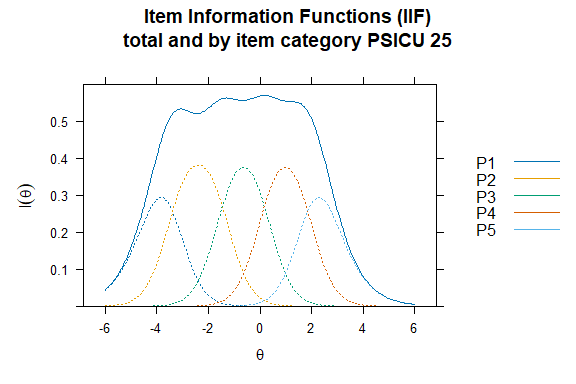

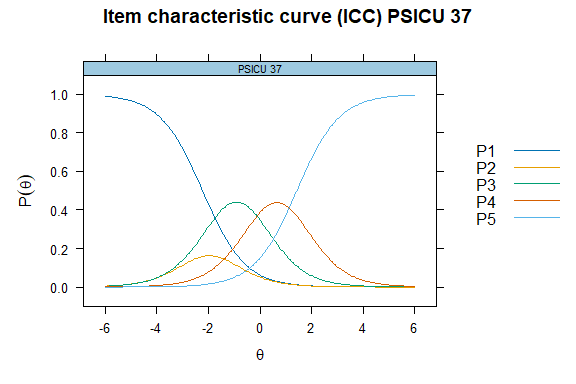

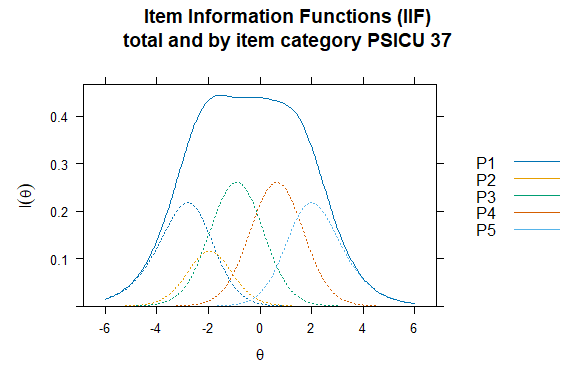

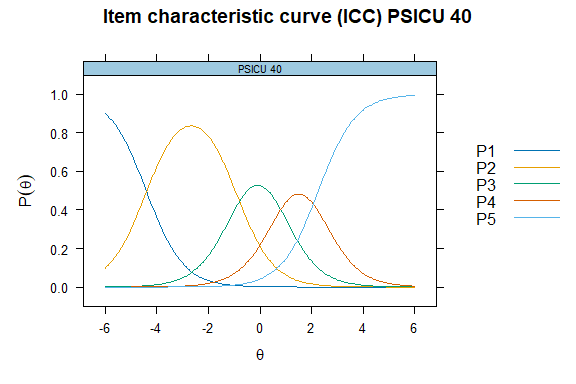

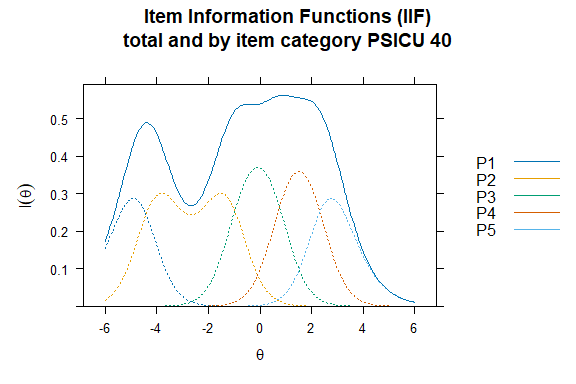

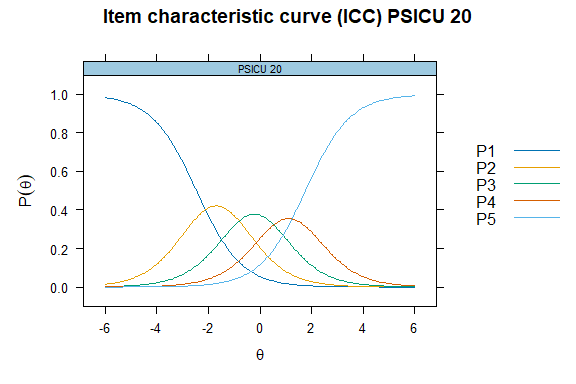

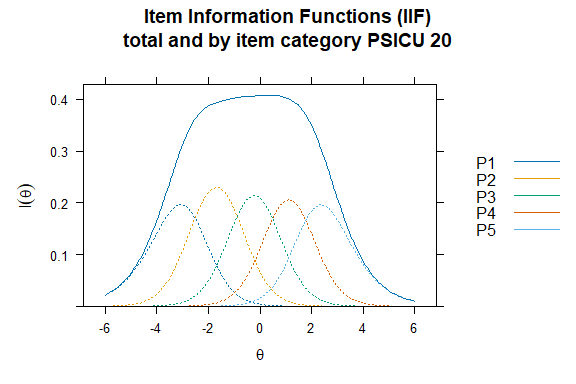

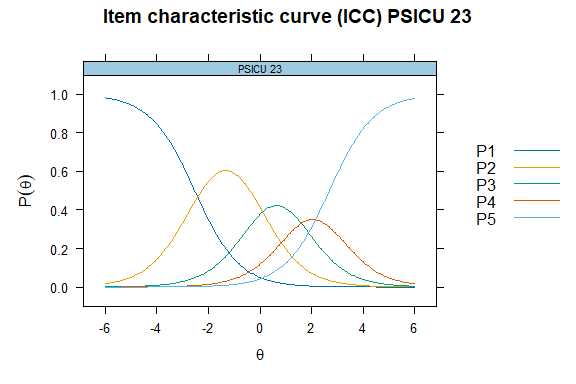

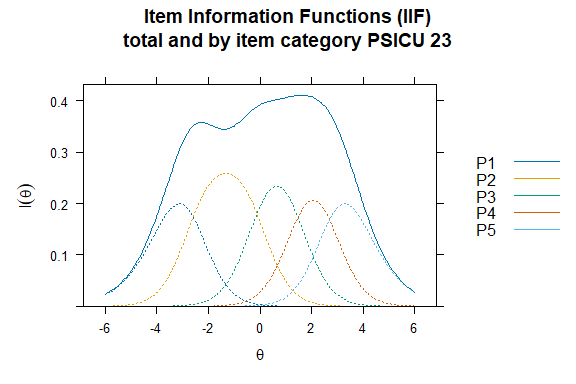

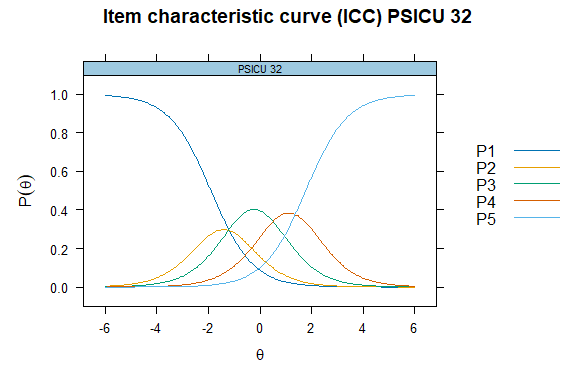

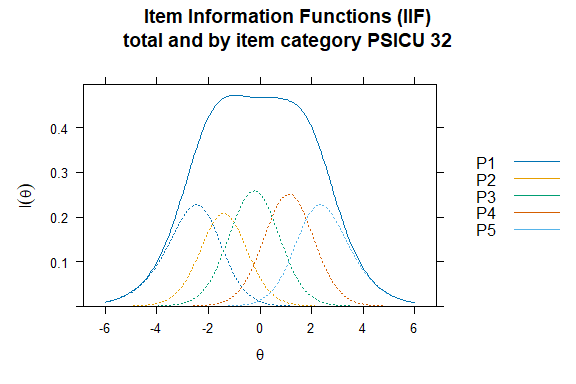

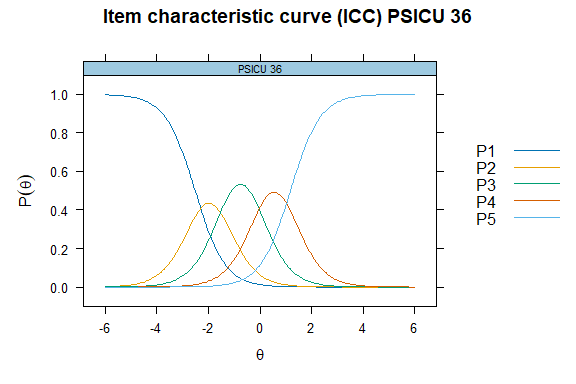

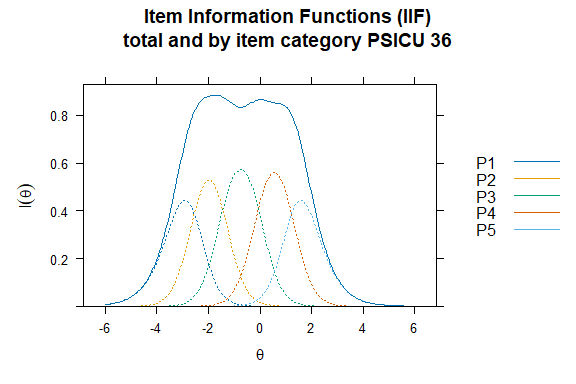

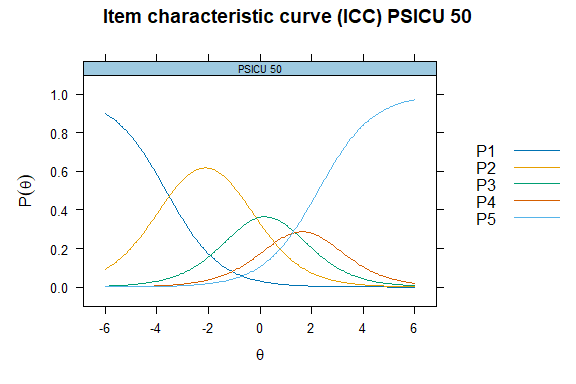

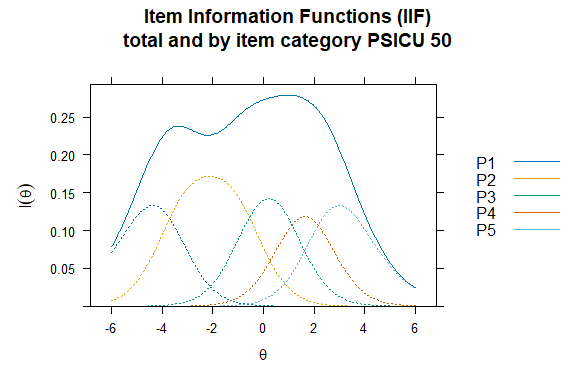

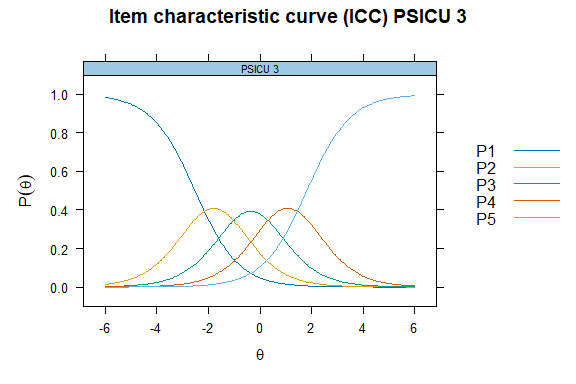

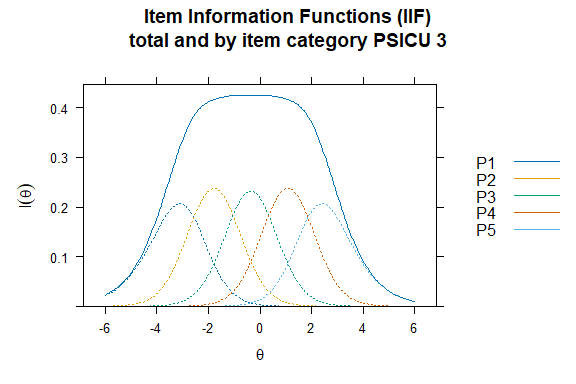

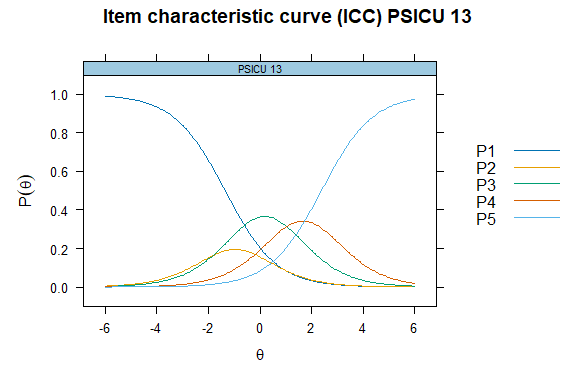

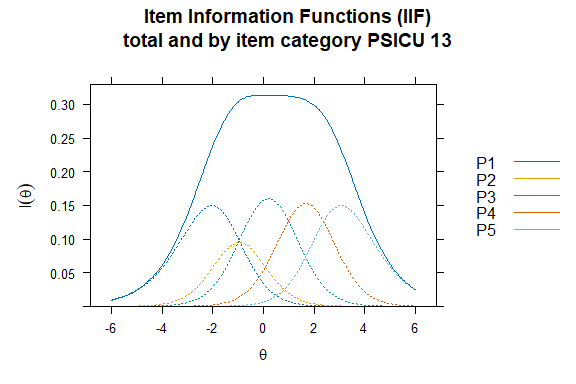

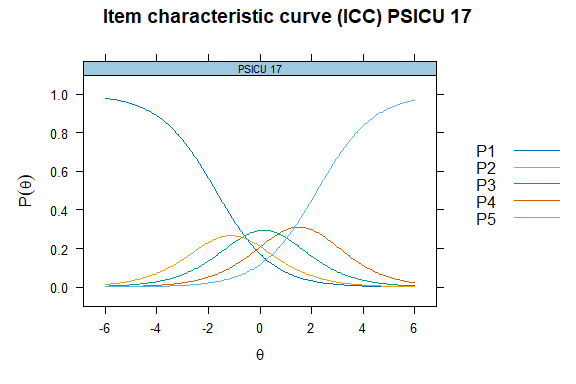

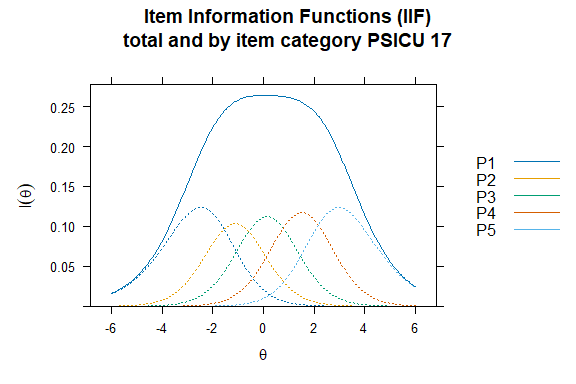

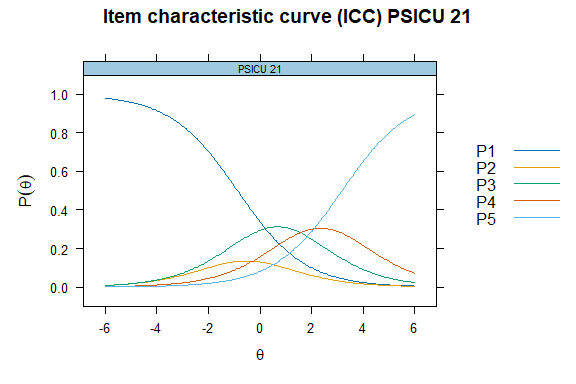

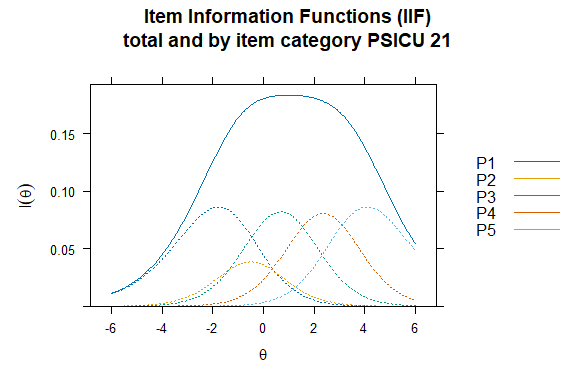

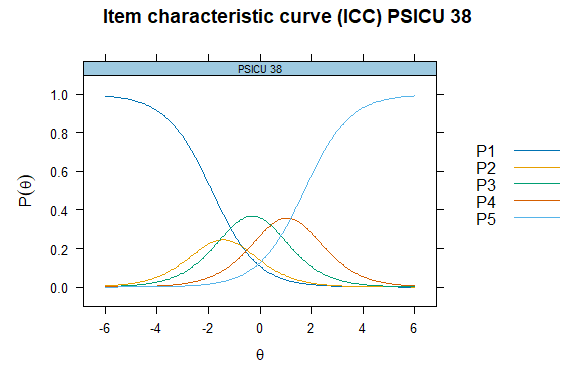

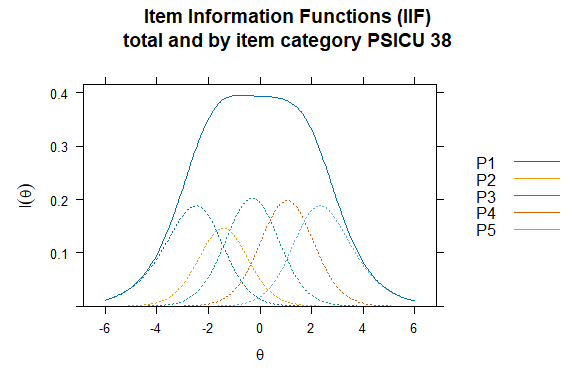


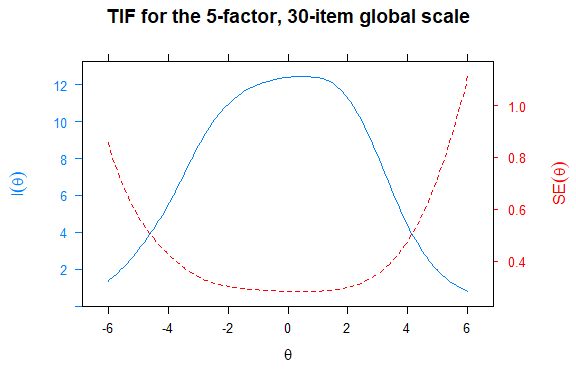


### 1.1.4 Model and item reliability

##
## Marginal empirical reliability: 0.924

## 1.2 IRT for factor 1 of the 5-factor model

### 1.2.1 IRT model fitting and comparison

##
##
## == IRT MODEL COMPARISON ==

|  | Modèle | G2 | AIC | BIC | LogLik |
| --- | --- | --- | --- | --- | --- |
| PCM | PCM | 12248.32 | 35153.92 | 35352.69 | -17539.96 |
| Graded | Graded | 11945.25 | 34866.86 | 35108.60 | -17388.43 |
| RSM | RSM | 14526.13 | 37383.73 | 37453.57 | -18678.87 |
| GPCM | GPCM | 12121.98 | 35043.58 | 35285.32 | -17476.79 |

### 1.2.2 Evaluation of the selected model

##
##
## == ITEM PARAMETERS ( Graded ) ==

|  | a | b1 | b2 | b3 | b4 |
| --- | --- | --- | --- | --- | --- |
| PSICU 4 | 1.464 | -1.751 | -1.173 | 0.277 | 1.866 |
| PSICU 6 | 1.028 | -2.119 | -0.203 | 1.207 | 2.693 |
| PSICU 9 | 1.301 | -1.823 | -0.601 | 1.030 | 2.615 |
| PSICU 10 | 1.797 | -1.714 | -0.686 | 0.750 | 2.191 |
| PSICU 26 | 1.310 | -1.633 | 0.352 | 1.775 | 2.970 |
| PSICU 27 | 1.773 | -2.576 | -0.408 | 1.195 | 2.637 |
| PSICU 35 | 1.653 | -1.319 | -0.545 | 0.744 | 2.050 |
| PSICU 41 | 1.008 | -4.000 | 1.687 | 3.304 | 4.632 |
| PSICU 42 | 1.500 | -2.922 | -0.146 | 1.722 | 3.156 |

##
##
## == ASSESSMENT OF MODEL FIT ==

| item | S_X2 | df.S_X2 | RMSEA.S_X2 | p.S_X2 |
| --- | --- | --- | --- | --- |
| PSICU 4 | 116.957 | 68 | 0.021 | 0.000 |
| PSICU 6 | 100.435 | 78 | 0.013 | 0.045 |
| PSICU 9 | 71.146 | 73 | 0.000 | 0.540 |
| PSICU 10 | 79.673 | 64 | 0.012 | 0.089 |
| PSICU 26 | 95.698 | 69 | 0.016 | 0.018 |
| PSICU 27 | 58.856 | 59 | 0.000 | 0.481 |
| PSICU 35 | 70.322 | 67 | 0.006 | 0.367 |
| PSICU 41 | 49.573 | 61 | 0.000 | 0.852 |
| PSICU 42 | 71.955 | 62 | 0.010 | 0.182 |

### 1.2.3 Charts of item characteristics


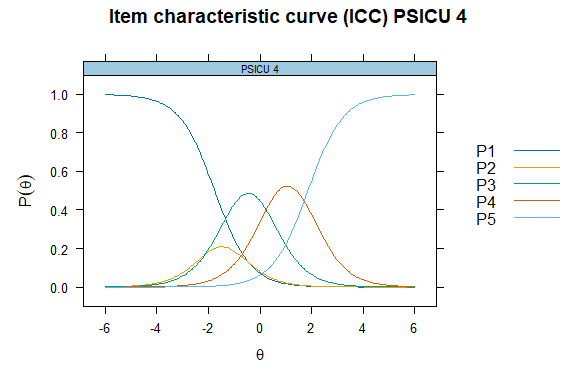

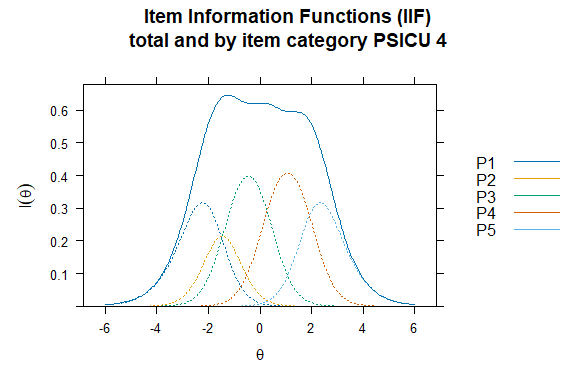

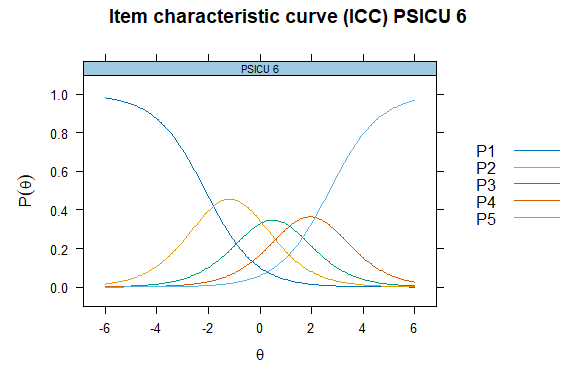

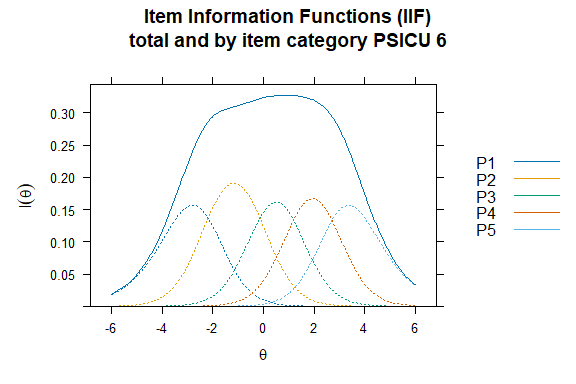

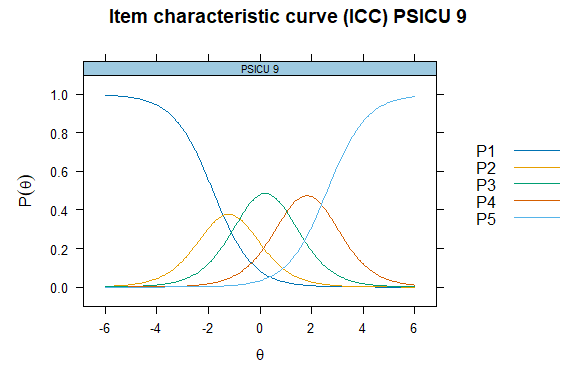

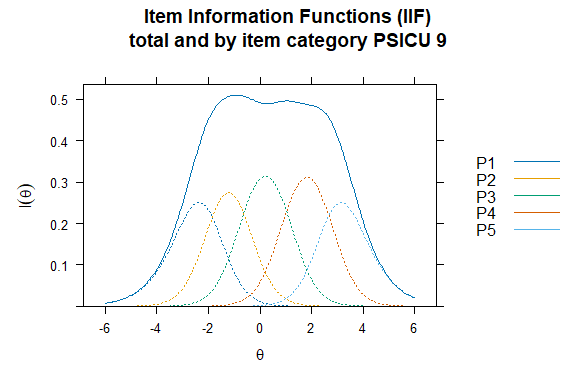

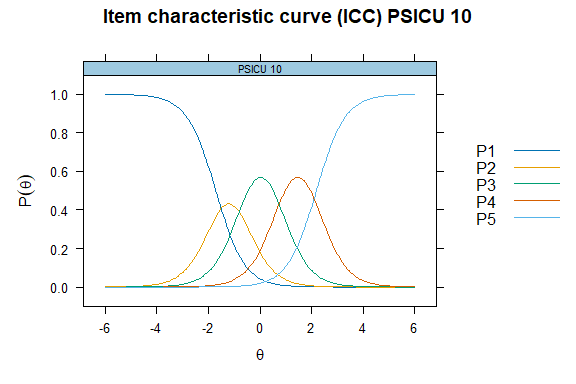

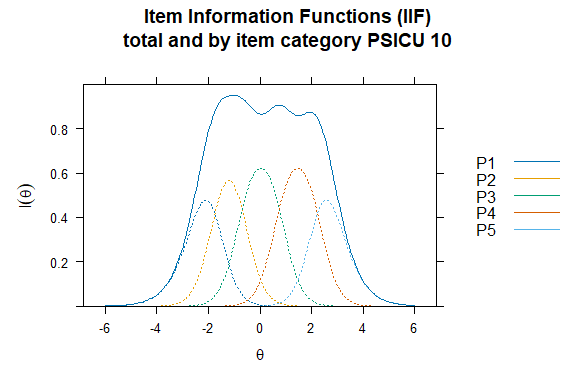

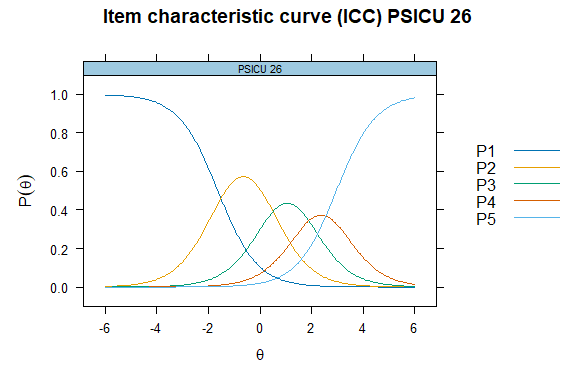

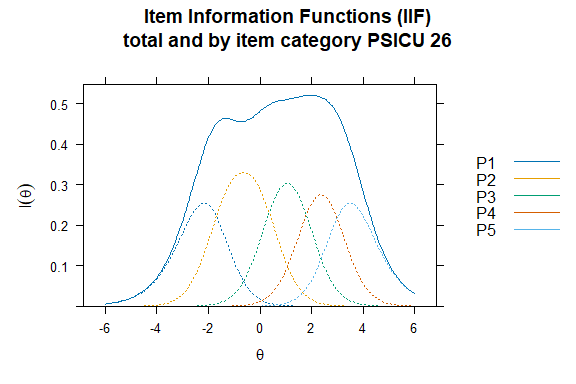

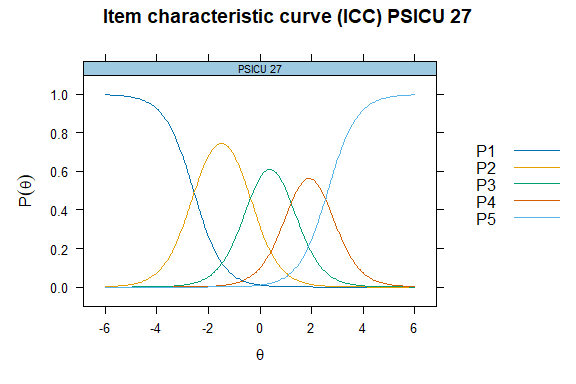

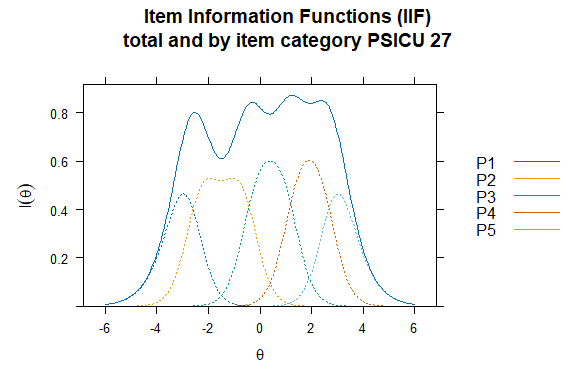

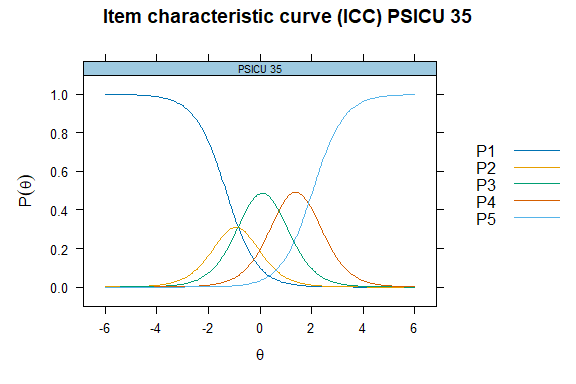

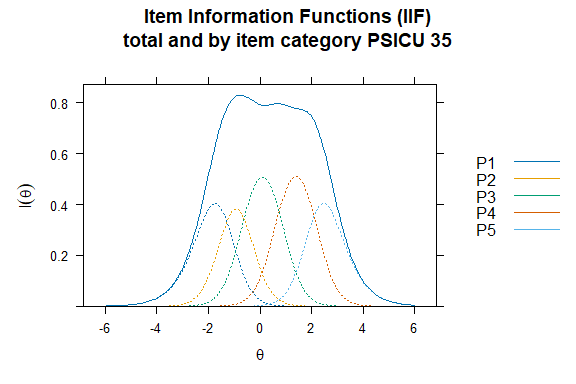

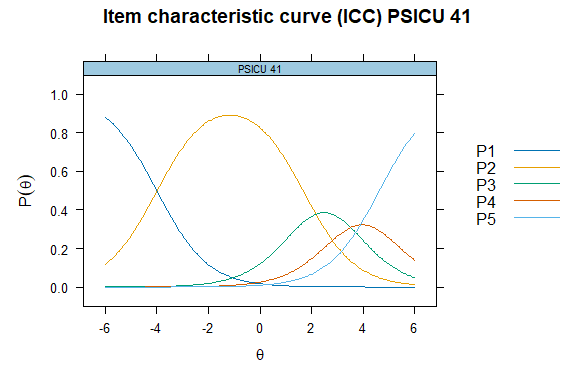

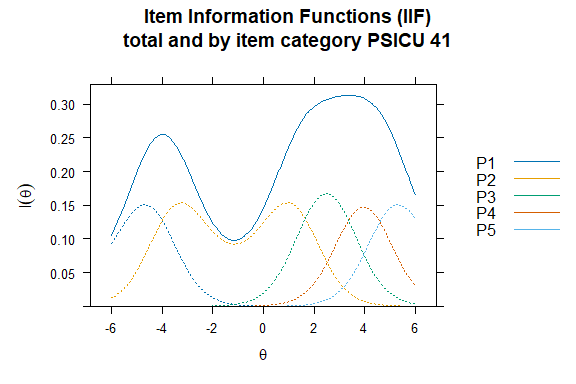

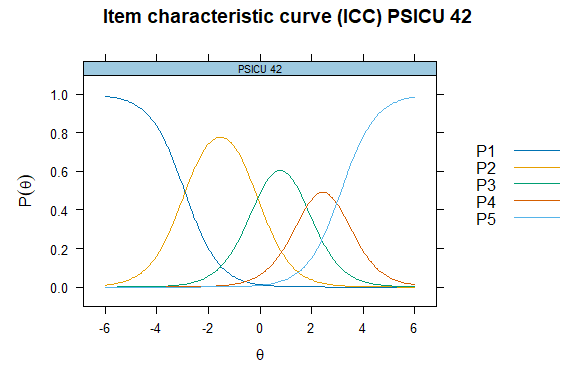

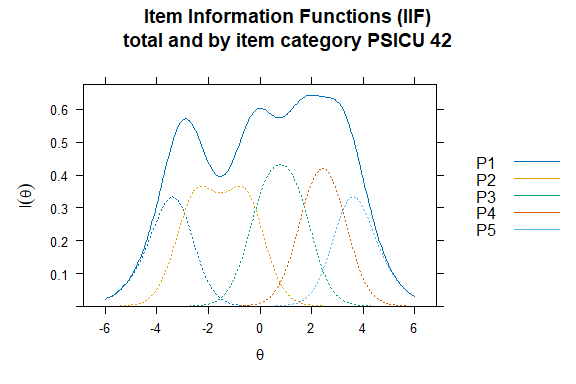


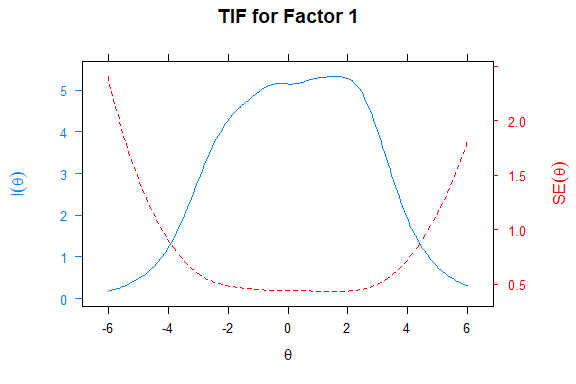


### 1.2.4 Model and item reliability

##
## Marginal empirical reliability: 0.835

## 1.3 IRT for factor 2 of the 5-factor model

### 1.3.1 IRT model fitting and comparison

##
##
## == IRT MODEL COMPARISON ==

|  | Modèle | G2 | AIC | BIC | LogLik |
| --- | --- | --- | --- | --- | --- |
| PCM | PCM | 4370.66 | 25494.00 | 25628.30 | -12722.00 |
| Graded | Graded | 4222.12 | 25355.47 | 25516.63 | -12647.73 |
| RSM | RSM | 5176.79 | 26270.13 | 26323.85 | -13125.07 |
| GPCM | GPCM | 4331.82 | 25465.17 | 25626.33 | -12702.58 |

### 1.3.2 Evaluation of the selected model

##
##
## == ITEM PARAMETERS ( Graded ) ==

|  | a | b1 | b2 | b3 | b4 |
| --- | --- | --- | --- | --- | --- |
| PSICU 5 | 1.326 | -2.825 | -1.585 | -0.044 | 1.417 |
| PSICU 7 | 1.692 | -4.254 | -1.488 | 0.184 | 1.704 |
| PSICU 28 | 1.620 | -1.936 | -0.254 | 0.953 | 2.258 |
| PSICU 29 | 1.466 | -1.174 | -0.582 | 0.623 | 1.784 |
| PSICU 34 | 1.567 | -0.780 | -0.096 | 1.031 | 2.209 |
| PSICU 45 | 1.498 | -2.629 | -0.896 | 0.724 | 2.159 |

##
##
## == ASSESSMENT OF MODEL FIT ==

| item | S_X2 | df.S_X2 | RMSEA.S_X2 | p.S_X2 |
| --- | --- | --- | --- | --- |
| PSICU 5 | 80.008 | 49 | 0.020 | 0.003 |
| PSICU 7 | 45.923 | 37 | 0.012 | 0.149 |
| PSICU 28 | 73.592 | 46 | 0.019 | 0.006 |
| PSICU 29 | 103.162 | 50 | 0.026 | 0.000 |
| PSICU 34 | 124.214 | 48 | 0.032 | 0.000 |
| PSICU 45 | 52.636 | 47 | 0.009 | 0.265 |

### 1.3.3 Charts of item characteristics


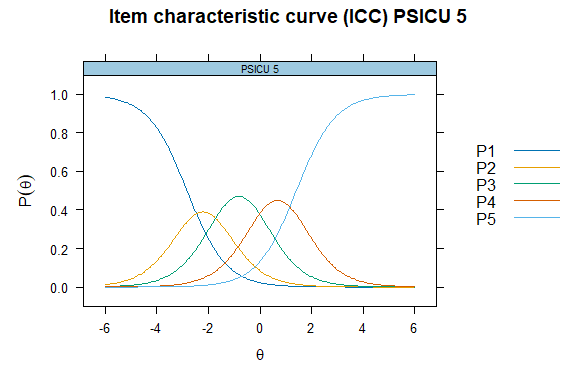

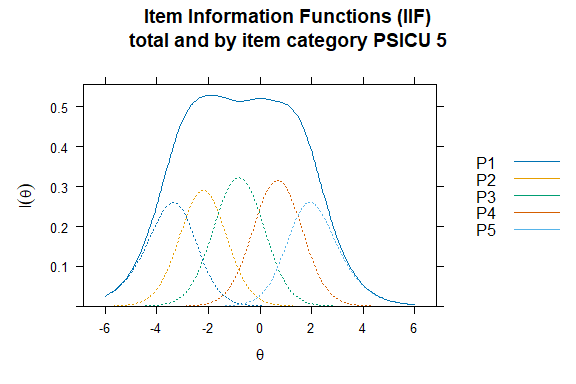

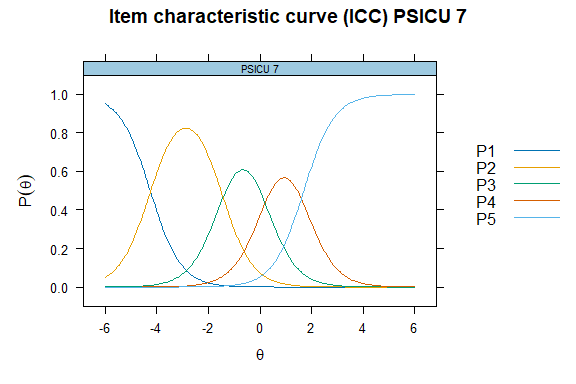

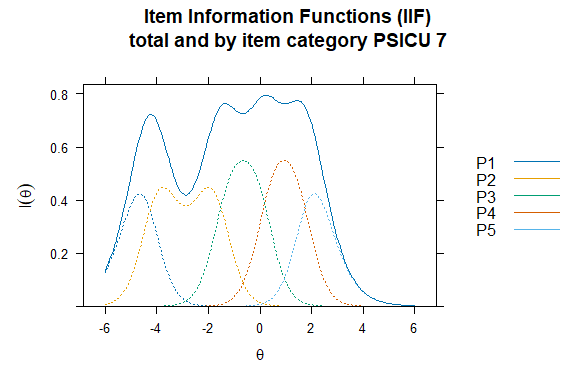

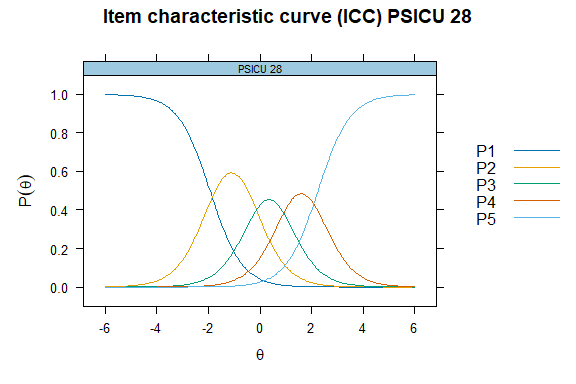

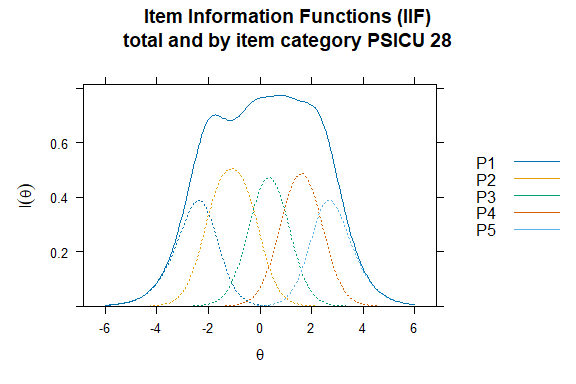

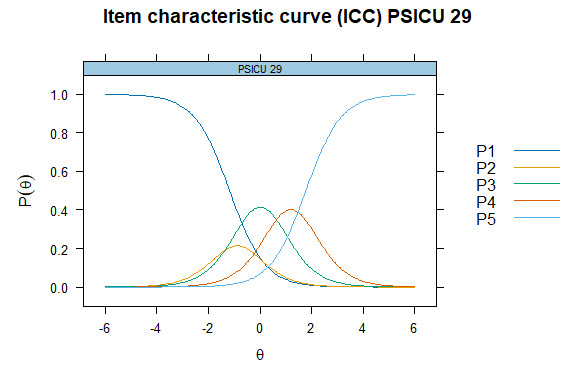

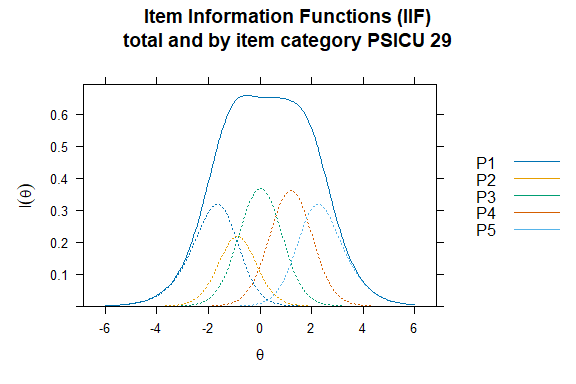

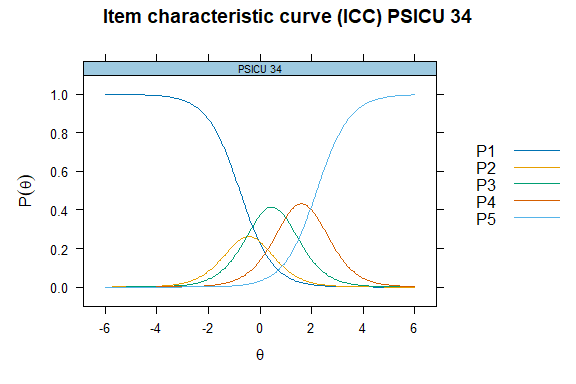

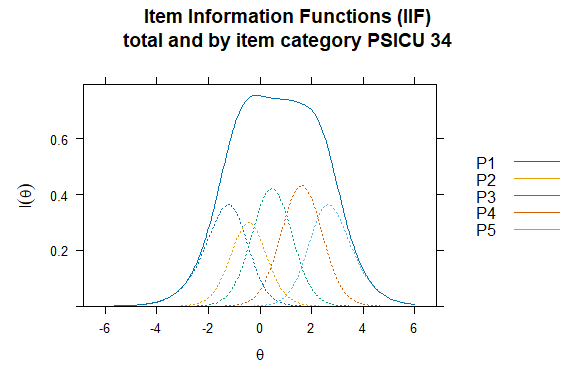

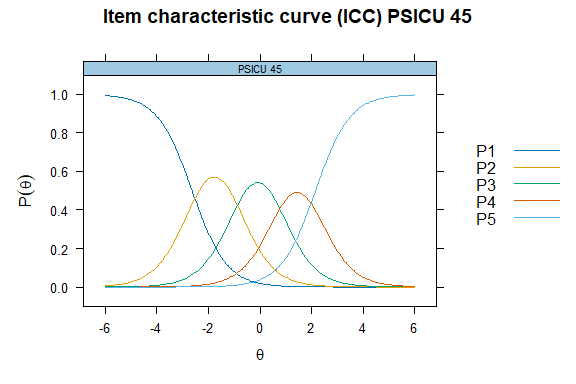

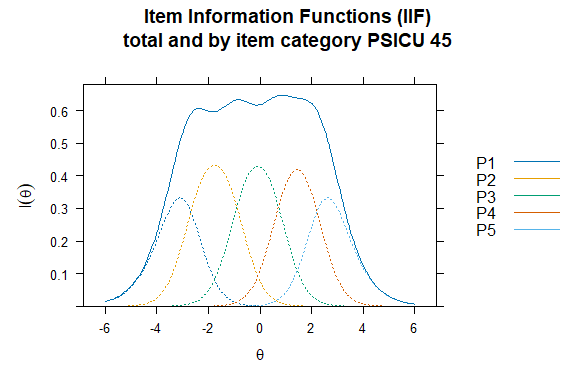


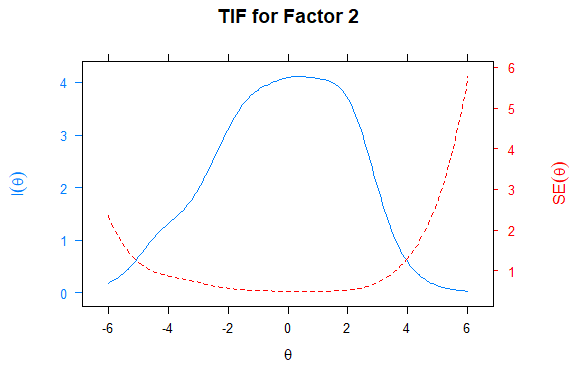


### 1.3.4 Model and item reliability

##
## Marginal empirical reliability: 0.796

## 1.4 IRT for factor 3 of the 5-factor model

### 1.4.1 IRT model fitting and comparison

##
##
## == IRT MODEL COMPARISON ==

|  | Modèle | G2 | AIC | BIC | LogLik |
| --- | --- | --- | --- | --- | --- |
| PCM | PCM | 1654.80 | 19439.41 | 19552.22 | -9698.70 |
| Graded | Graded | 1474.71 | 19267.32 | 19401.62 | -9608.66 |
| RSM | RSM | 2356.44 | 20117.05 | 20165.40 | -10049.53 |
| GPCM | GPCM | 1595.08 | 19387.69 | 19521.99 | -9668.84 |

### 1.4.2 Evaluation of the selected model

##
##
## == ITEM PARAMETERS ( Graded ) ==

|  | a | b1 | b2 | b3 | b4 |
| --- | --- | --- | --- | --- | --- |
| PSICU 16 | 1.809 | -2.499 | -1.799 | -0.390 | 0.754 |
| PSICU 18 | 2.378 | -3.148 | -1.048 | 0.328 | 1.424 |
| PSICU 25 | 1.639 | -3.058 | -1.364 | 0.172 | 1.672 |
| PSICU 37 | 1.737 | -1.825 | -1.397 | -0.108 | 1.173 |
| PSICU 40 | 2.086 | -3.471 | -0.783 | 0.639 | 1.859 |

##
##
## == ASSESSMENT OF MODEL FIT ==

| item | S_X2 | df.S_X2 | RMSEA.S_X2 | p.S_X2 |
| --- | --- | --- | --- | --- |
| PSICU 16 | 44.010 | 33 | 0.014 | 0.095 |
| PSICU 18 | 26.040 | 26 | 0.001 | 0.461 |
| PSICU 25 | 56.174 | 31 | 0.023 | 0.004 |
| PSICU 37 | 77.434 | 34 | 0.028 | 0.000 |
| PSICU 40 | 33.185 | 23 | 0.017 | 0.078 |

### 1.4.3 Charts of item characteristics


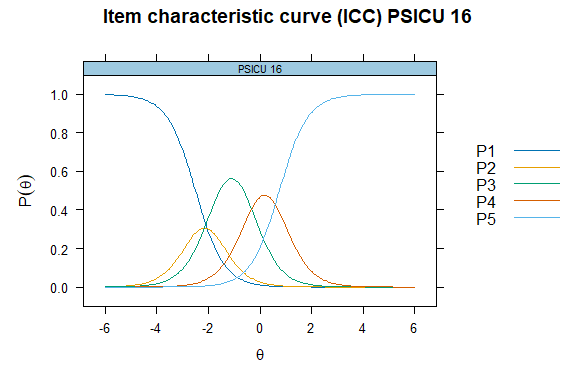

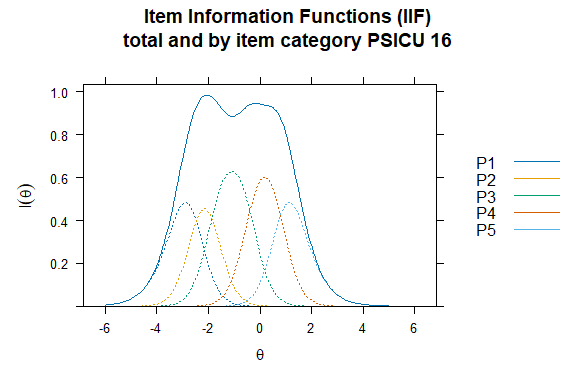

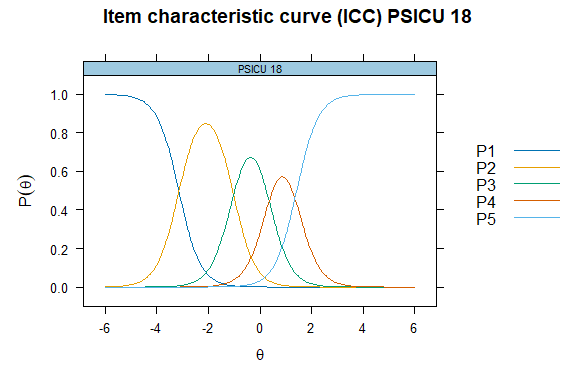

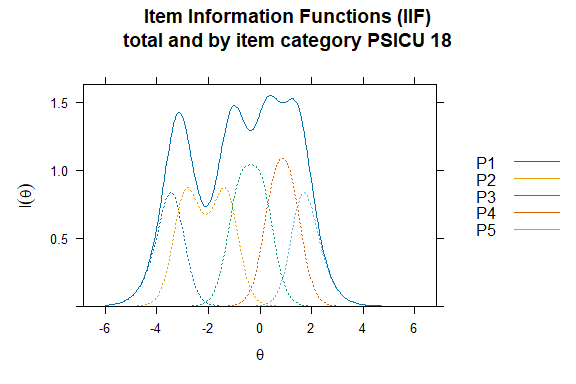

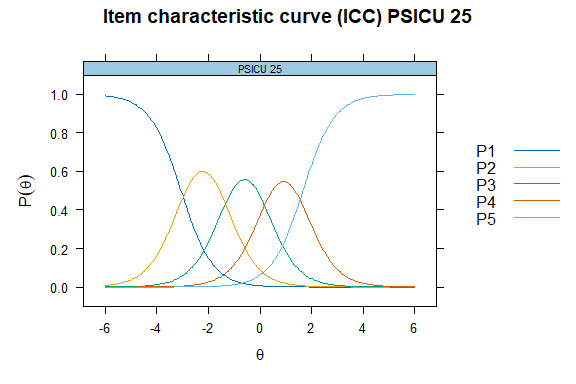

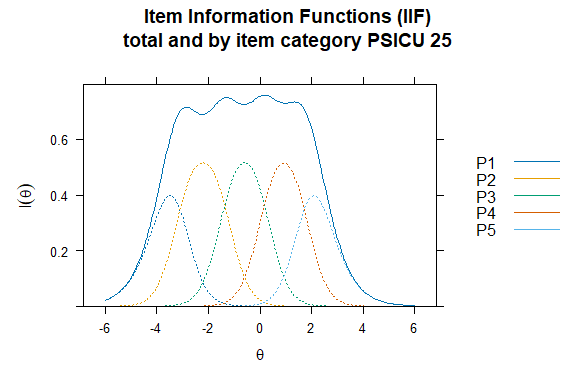

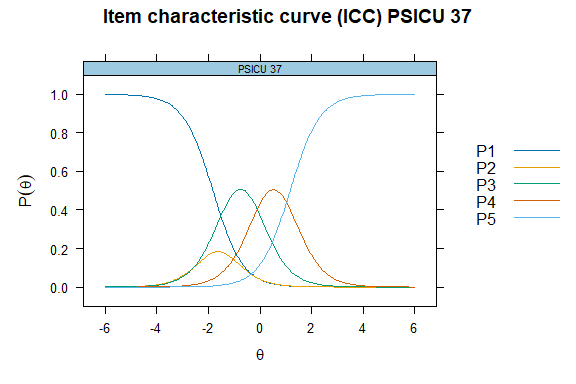


### 1.4.4 Model and item reliability

##
## Marginal empirical reliability: 0.827

## 1.5 IRT for factor 4 of the 5-factor model

### 1.5.1 IRT model fitting and comparison

##
##
## == IRT MODEL COMPARISON ==

|  | Modèle | G2 | AIC | BIC | LogLik |
| --- | --- | --- | --- | --- | --- |
| PCM | PCM | 2428.05 | 21785.61 | 21898.42 | -10871.80 |
| Graded | Graded | 2292.03 | 21657.58 | 21791.89 | -10803.79 |
| RSM | RSM | 2787.98 | 22121.53 | 22169.88 | -11051.77 |
| GPCM | GPCM | 2354.73 | 21720.29 | 21854.59 | -10835.14 |

### 1.5.2 Evaluation of the selected model

##
##
## == ITEM PARAMETERS ( Graded ) ==

|  | a | b1 | b2 | b3 | b4 |
| --- | --- | --- | --- | --- | --- |
| PSICU 20 | 1.898 | -1.834 | -0.709 | 0.345 | 1.352 |
| PSICU 23 | 1.682 | -2.020 | -0.120 | 1.149 | 2.157 |
| PSICU 32 | 1.398 | -1.756 | -0.844 | 0.430 | 1.654 |
| PSICU 36 | 1.817 | -2.445 | -1.394 | -0.056 | 1.166 |
| PSICU 50 | 1.221 | -2.991 | -0.506 | 0.855 | 1.918 |

##
##
## == ASSESSMENT OF MODEL FIT ==

| item | S_X2 | df.S_X2 | RMSEA.S_X2 | p.S_X2 |
| --- | --- | --- | --- | --- |
| PSICU 20 | 56.668 | 35 | 0.020 | 0.012 |
| PSICU 23 | 63.332 | 34 | 0.023 | 0.002 |
| PSICU 32 | 97.109 | 40 | 0.030 | 0.000 |
| PSICU 36 | 54.781 | 34 | 0.020 | 0.013 |
| PSICU 50 | 81.464 | 38 | 0.027 | 0.000 |

### 1.5.3 Charts of item characteristics

### 1.5.4 Model and item reliability

##
## Marginal empirical reliability: 0.786

## 1.6 IRT for factor 5 of the 5-factor model

### 1.6.1 IRT model fitting and comparison

##
##
## == IRT MODEL COMPARISON ==

|  | Modèle | G2 | AIC | BIC | LogLik |
| --- | --- | --- | --- | --- | --- |
| PCM | PCM | 2860.15 | 23285.86 | 23398.67 | -11621.93 |
| Graded | Graded | 2751.02 | 23184.72 | 23319.02 | -11567.36 |
| RSM | RSM | 3083.32 | 23485.03 | 23533.38 | -11733.51 |
| GPCM | GPCM | 2821.45 | 23255.15 | 23389.46 | -11602.58 |

### 1.6.2 Evaluation of the selected model

##
##
## == ITEM PARAMETERS ( Graded ) ==

|  | a | b1 | b2 | b3 | b4 |
| --- | --- | --- | --- | --- | --- |
| PSICU 3 | 1.194 | -2.484 | -1.027 | 0.356 | 1.819 |
| PSICU 13 | 1.599 | -1.006 | -0.429 | 0.716 | 1.790 |
| PSICU 17 | 1.858 | -1.101 | -0.351 | 0.514 | 1.452 |
| PSICU 21 | 1.265 | -0.609 | -0.122 | 1.051 | 2.200 |
| PSICU 38 | 1.386 | -1.602 | -0.836 | 0.346 | 1.515 |

##
##
## == ASSESSMENT OF MODEL FIT ==

| item | S_X2 | df.S_X2 | RMSEA.S_X2 | p.S_X2 |
| --- | --- | --- | --- | --- |
| PSICU 3 | 61.615 | 43 | 0.017 | 0.033 |
| PSICU 13 | 45.869 | 42 | 0.008 | 0.315 |
| PSICU 17 | 64.291 | 40 | 0.020 | 0.009 |
| PSICU 21 | 115.855 | 43 | 0.033 | 0.000 |
| PSICU 38 | 54.389 | 42 | 0.014 | 0.095 |

### 1.6.3 Charts of item characteristics

### 1.6.4 Model and item reliability

##
## Marginal empirical reliability: 0.756

# 2 Six-factor model with 31 items (model #9) estimated on the original population of the scale (nurses, physicians and medical residents)

## 2.1 IRT for the entire scale (31 items)

### 2.1.1 IRT model fitting and comparison

##
##
## == IRT MODEL COMPARISON ==

|  | Modèle | G2 | AIC | BIC | LogLik |
| --- | --- | --- | --- | --- | --- |
| PCM | PCM | 106720.2 | 130428.2 | 131099.8 | -65089.13 |
| Graded | Graded | 105766.8 | 129534.9 | 130367.6 | -64612.44 |
| RSM | RSM | 113642.8 | 137170.9 | 137358.9 | -68550.43 |
| GPCM | GPCM | 106235.0 | 130003.1 | 130835.8 | -64846.54 |

### 2.1.2 Evaluation of the selected model

##
##
## == ITEM PARAMETERS ( Graded ) ==

|  | a | b1 | b2 | b3 | b4 |
| --- | --- | --- | --- | --- | --- |
| PSICU 42 | 1.330 | -3.146 | -0.171 | 1.843 | 3.433 |
| PSICU 27 | 1.492 | -2.853 | -0.472 | 1.297 | 2.916 |
| PSICU 10 | 1.283 | -2.081 | -0.850 | 0.872 | 2.665 |
| PSICU 4 | 1.191 | -2.017 | -1.369 | 0.286 | 2.123 |
| PSICU 9 | 1.106 | -2.042 | -0.692 | 1.129 | 2.937 |
| PSICU 35 | 1.408 | -1.459 | -0.635 | 0.775 | 2.249 |
| PSICU 41 | 0.821 | -4.725 | 1.973 | 3.909 | 5.513 |
| PSICU 26 | 1.033 | -1.918 | 0.392 | 2.074 | 3.529 |
| PSICU 34 | 1.140 | -0.934 | -0.102 | 1.242 | 2.661 |
| PSICU 28 | 1.295 | -2.212 | -0.286 | 1.071 | 2.561 |
| PSICU 29 | 1.132 | -1.362 | -0.657 | 0.739 | 2.074 |
| PSICU 7 | 1.422 | -4.837 | -1.635 | 0.209 | 1.855 |
| PSICU 45 | 1.276 | -2.930 | -0.976 | 0.789 | 2.358 |
| PSICU 5 | 1.162 | -3.110 | -1.729 | -0.056 | 1.520 |
| PSICU 18 | 0.977 | -5.609 | -1.646 | 0.525 | 2.291 |
| PSICU 40 | 1.278 | -4.696 | -0.978 | 0.802 | 2.397 |
| PSICU 37 | 1.122 | -2.337 | -1.763 | -0.122 | 1.508 |
| PSICU 16 | 1.156 | -3.268 | -2.306 | -0.498 | 0.948 |
| PSICU 25 | 1.333 | -3.463 | -1.494 | 0.186 | 1.856 |
| PSICU 50 | 0.936 | -3.677 | -0.601 | 1.026 | 2.285 |
| PSICU 20 | 1.149 | -2.467 | -0.900 | 0.481 | 1.770 |
| PSICU 23 | 1.191 | -2.482 | -0.117 | 1.410 | 2.648 |
| PSICU 17 | 0.931 | -1.689 | -0.509 | 0.801 | 2.190 |
| PSICU 13 | 0.978 | -1.369 | -0.567 | 0.988 | 2.433 |
| PSICU 21 | 0.808 | -0.831 | -0.151 | 1.467 | 3.043 |
| PSICU 38 | 1.069 | -1.927 | -1.006 | 0.411 | 1.781 |
| PSICU 3 | 1.218 | -2.465 | -1.029 | 0.345 | 1.784 |
| PSICU 32 | 1.274 | -1.858 | -0.884 | 0.466 | 1.749 |
| PSICU 8 | 0.961 | -1.477 | -0.762 | 0.814 | 2.425 |
| PSICU 15 | 0.948 | -1.075 | -0.289 | 0.718 | 1.941 |
| PSICU 6 | 0.960 | -2.230 | -0.236 | 1.241 | 2.831 |

##
##
## == ASSESSMENT OF MODEL FIT ==

| item | S_X2 | df.S_X2 | RMSEA.S_X2 | p.S_X2 |
| --- | --- | --- | --- | --- |
| PSICU 42 | 175.431 | 133 | 0.014 | 0.008 |
| PSICU 27 | 155.596 | 143 | 0.007 | 0.223 |
| PSICU 10 | 219.892 | 197 | 0.009 | 0.126 |
| PSICU 4 | 234.034 | 207 | 0.009 | 0.096 |
| PSICU 9 | 216.801 | 202 | 0.007 | 0.226 |
| PSICU 35 | 253.259 | 188 | 0.015 | 0.001 |
| PSICU 41 | 160.837 | 123 | 0.014 | 0.012 |
| PSICU 26 | 226.773 | 194 | 0.010 | 0.054 |
| PSICU 34 | 292.405 | 215 | 0.015 | 0.000 |
| PSICU 28 | 227.492 | 196 | 0.010 | 0.061 |
| PSICU 29 | 260.066 | 223 | 0.010 | 0.045 |
| PSICU 7 | 149.933 | 155 | 0.000 | 0.600 |
| PSICU 45 | 191.589 | 190 | 0.002 | 0.454 |
| PSICU 5 | 242.026 | 195 | 0.012 | 0.012 |
| PSICU 18 | 174.983 | 182 | 0.000 | 0.632 |
| PSICU 40 | 173.486 | 165 | 0.006 | 0.310 |
| PSICU 37 | 214.181 | 210 | 0.004 | 0.407 |
| PSICU 16 | 195.204 | 191 | 0.004 | 0.402 |
| PSICU 25 | 189.593 | 169 | 0.009 | 0.133 |
| PSICU 50 | 236.612 | 210 | 0.009 | 0.100 |
| PSICU 20 | 220.182 | 216 | 0.003 | 0.408 |
| PSICU 23 | 206.739 | 190 | 0.007 | 0.193 |
| PSICU 17 | 263.834 | 241 | 0.008 | 0.149 |
| PSICU 13 | 241.483 | 235 | 0.004 | 0.372 |
| PSICU 21 | 289.103 | 234 | 0.012 | 0.008 |
| PSICU 38 | 229.435 | 221 | 0.005 | 0.334 |
| PSICU 3 | 230.197 | 205 | 0.009 | 0.109 |
| PSICU 32 | 233.888 | 208 | 0.009 | 0.105 |
| PSICU 8 | 262.880 | 231 | 0.009 | 0.073 |
| PSICU 15 | 268.298 | 234 | 0.010 | 0.061 |
| PSICU 6 | 244.941 | 223 | 0.008 | 0.150 |

### 2.1.3 Charts of item characteristics

### 2.1.4 Model and item reliability

##
## Marginal empirical reliability: 0.922

## 2.2 IRT for factor 1 of the 6-factor model

### 2.2.1 IRT model fitting and comparison

##
##
## == IRT MODEL COMPARISON ==

|  | Modèle | G2 | AIC | BIC | LogLik |
| --- | --- | --- | --- | --- | --- |
| PCM | PCM | 8477.45 | 30639.97 | 30817.25 | -15286.98 |
| Graded | Graded | 8209.62 | 30386.15 | 30601.03 | -15153.07 |
| RSM | RSM | 10677.68 | 32798.21 | 32862.67 | -16387.10 |
| GPCM | GPCM | 8382.36 | 30558.89 | 30773.77 | -15239.44 |

### 2.2.2 Evaluation of the selected model

##
##
## == ITEM PARAMETERS ( Graded ) ==

|  | a | b1 | b2 | b3 | b4 |
| --- | --- | --- | --- | --- | --- |
| PSICU 42 | 1.479 | -2.948 | -0.148 | 1.736 | 3.184 |
| PSICU 27 | 1.881 | -2.499 | -0.398 | 1.163 | 2.563 |
| PSICU 10 | 1.800 | -1.713 | -0.688 | 0.748 | 2.192 |
| PSICU 4 | 1.440 | -1.769 | -1.186 | 0.276 | 1.883 |
| PSICU 9 | 1.214 | -1.906 | -0.628 | 1.072 | 2.735 |
| PSICU 35 | 1.693 | -1.304 | -0.545 | 0.729 | 2.026 |
| PSICU 41 | 0.997 | -4.038 | 1.702 | 3.332 | 4.671 |
| PSICU 26 | 1.323 | -1.625 | 0.345 | 1.764 | 2.956 |

##
##
## == ASSESSMENT OF MODEL FIT ==

| item | S_X2 | df.S_X2 | RMSEA.S_X2 | p.S_X2 |
| --- | --- | --- | --- | --- |
| PSICU 42 | 92.616 | 56 | 0.020 | 0.002 |
| PSICU 27 | 50.725 | 53 | 0.000 | 0.563 |
| PSICU 10 | 81.123 | 56 | 0.017 | 0.016 |
| PSICU 4 | 108.014 | 60 | 0.022 | 0.000 |
| PSICU 9 | 73.553 | 65 | 0.009 | 0.218 |
| PSICU 35 | 82.989 | 57 | 0.017 | 0.014 |
| PSICU 41 | 65.486 | 59 | 0.008 | 0.262 |
| PSICU 26 | 98.154 | 64 | 0.018 | 0.004 |

### 2.2.3 Charts of item characteristics

### 2.2.4 Model and item reliability

##
## Marginal empirical reliability: 0.827

## 2.3 IRT for factor 2 of the 6-factor model

### 2.3.1 IRT model fitting and comparison

##
##
## == IRT MODEL COMPARISON ==

|  | Modèle | G2 | AIC | BIC | LogLik |
| --- | --- | --- | --- | --- | --- |
| PCM | PCM | 4370.66 | 25494.00 | 25628.30 | -12722.00 |
| Graded | Graded | 4222.12 | 25355.47 | 25516.63 | -12647.73 |
| RSM | RSM | 5176.79 | 26270.13 | 26323.85 | -13125.07 |
| GPCM | GPCM | 4331.82 | 25465.17 | 25626.33 | -12702.58 |

### 2.3.2 Evaluation of the selected model

##
##
## == ITEM PARAMETERS ( Graded ) ==

|  | a | b1 | b2 | b3 | b4 |
| --- | --- | --- | --- | --- | --- |
| PSICU 34 | 1.567 | -0.780 | -0.096 | 1.031 | 2.209 |
| PSICU 28 | 1.620 | -1.936 | -0.254 | 0.953 | 2.258 |
| PSICU 29 | 1.466 | -1.174 | -0.582 | 0.623 | 1.784 |
| PSICU 7 | 1.692 | -4.254 | -1.488 | 0.184 | 1.704 |
| PSICU 45 | 1.498 | -2.629 | -0.896 | 0.724 | 2.159 |
| PSICU 5 | 1.326 | -2.825 | -1.585 | -0.044 | 1.417 |

##
##
## == ASSESSMENT OF MODEL FIT ==

| item | S_X2 | df.S_X2 | RMSEA.S_X2 | p.S_X2 |
| --- | --- | --- | --- | --- |
| PSICU 34 | 124.214 | 48 | 0.032 | 0.000 |
| PSICU 28 | 73.592 | 46 | 0.019 | 0.006 |
| PSICU 29 | 103.162 | 50 | 0.026 | 0.000 |
| PSICU 7 | 45.923 | 37 | 0.012 | 0.149 |
| PSICU 45 | 52.636 | 47 | 0.009 | 0.265 |
| PSICU 5 | 80.008 | 49 | 0.020 | 0.003 |

### 2.3.3 Charts of item characteristics

### 2.3.4 Model and item reliability

##
## Marginal empirical reliability: 0.796

## 2.4 IRT for factor 3 of the 6-factor model

### 2.4.1 IRT model fitting and comparison

##
##
## == IRT MODEL COMPARISON ==

|  | Modèle | G2 | AIC | BIC | LogLik |
| --- | --- | --- | --- | --- | --- |
| PCM | PCM | 1654.80 | 19439.41 | 19552.22 | -9698.70 |
| Graded | Graded | 1474.71 | 19267.32 | 19401.62 | -9608.66 |
| RSM | RSM | 2356.44 | 20117.05 | 20165.40 | -10049.53 |
| GPCM | GPCM | 1595.08 | 19387.69 | 19521.99 | -9668.84 |

### 2.4.2 Evaluation of the selected model

##
##
## == ITEM PARAMETERS ( Graded ) ==

|  | a | b1 | b2 | b3 | b4 |
| --- | --- | --- | --- | --- | --- |
| PSICU 18 | 2.378 | -3.148 | -1.048 | 0.328 | 1.424 |
| PSICU 40 | 2.086 | -3.471 | -0.783 | 0.639 | 1.859 |
| PSICU 37 | 1.737 | -1.825 | -1.397 | -0.108 | 1.173 |
| PSICU 16 | 1.809 | -2.499 | -1.799 | -0.390 | 0.754 |
| PSICU 25 | 1.639 | -3.058 | -1.364 | 0.172 | 1.672 |

##
##
## == ASSESSMENT OF MODEL FIT ==

| item | S_X2 | df.S_X2 | RMSEA.S_X2 | p.S_X2 |
| --- | --- | --- | --- | --- |
| PSICU 18 | 26.040 | 26 | 0.001 | 0.461 |
| PSICU 40 | 33.185 | 23 | 0.017 | 0.078 |
| PSICU 37 | 77.434 | 34 | 0.028 | 0.000 |
| PSICU 16 | 44.010 | 33 | 0.014 | 0.095 |
| PSICU 25 | 56.174 | 31 | 0.023 | 0.004 |

### 2.4.3 Charts of item characteristics

### 2.4.4 Model and item reliability

##
## Marginal empirical reliability: 0.827

## 2.5 IRT for factor 4 of the 6-factor model

### 2.5.1 IRT model fitting and comparison

##
##
## == IRT MODEL COMPARISON ==

|  | Modèle | G2 | AIC | BIC | LogLik |
| --- | --- | --- | --- | --- | --- |
| PCM | PCM | 400.72 | 13121.54 | 13191.38 | -6547.77 |
| Graded | Graded | 323.49 | 13048.31 | 13128.89 | -6509.15 |
| RSM | RSM | 501.11 | 13209.93 | 13247.53 | -6597.96 |
| GPCM | GPCM | 330.19 | 13055.00 | 13135.58 | -6512.50 |

### 2.5.2 Evaluation of the selected model

##
##
## == ITEM PARAMETERS ( Graded ) ==

|  | a | b1 | b2 | b3 | b4 |
| --- | --- | --- | --- | --- | --- |
| PSICU 50 | 1.459 | -2.657 | -0.451 | 0.778 | 1.730 |
| PSICU 20 | 3.399 | -1.513 | -0.600 | 0.291 | 1.128 |
| PSICU 23 | 1.215 | -2.454 | -0.130 | 1.375 | 2.596 |

##
##
## == ASSESSMENT OF MODEL FIT ==

| item | S_X2 | df.S_X2 | RMSEA.S_X2 | p.S_X2 |
| --- | --- | --- | --- | --- |
| PSICU 50 | 45.010 | 12 | 0.042 | 0.000 |
| PSICU 20 | 26.941 | 10 | 0.033 | 0.003 |
| PSICU 23 | 41.095 | 13 | 0.037 | 0.000 |

### 2.5.3 Charts of item characteristics

### 2.5.4 Model and item reliability

##
## Marginal empirical reliability: 0.782

## 2.6 IRT for factor 5 of the 6-factor model

### 2.6.1 IRT model fitting and comparison

##
##
## == IRT MODEL COMPARISON ==

|  | Modèle | G2 | AIC | BIC | LogLik |
| --- | --- | --- | --- | --- | --- |
| PCM | PCM | 2860.15 | 23285.86 | 23398.67 | -11621.93 |
| Graded | Graded | 2751.02 | 23184.72 | 23319.02 | -11567.36 |
| RSM | RSM | 3083.32 | 23485.03 | 23533.38 | -11733.51 |
| GPCM | GPCM | 2821.45 | 23255.15 | 23389.46 | -11602.58 |

### 2.6.2 Evaluation of the selected model

##
##
## == ITEM PARAMETERS ( Graded ) ==

|  | a | b1 | b2 | b3 | b4 |
| --- | --- | --- | --- | --- | --- |
| PSICU 17 | 1.858 | -1.101 | -0.351 | 0.514 | 1.452 |
| PSICU 13 | 1.599 | -1.006 | -0.429 | 0.716 | 1.790 |
| PSICU 21 | 1.265 | -0.609 | -0.122 | 1.051 | 2.200 |
| PSICU 38 | 1.386 | -1.602 | -0.836 | 0.346 | 1.515 |
| PSICU 3 | 1.194 | -2.484 | -1.027 | 0.356 | 1.819 |

##
##
## == ASSESSMENT OF MODEL FIT ==

| item | S_X2 | df.S_X2 | RMSEA.S_X2 | p.S_X2 |
| --- | --- | --- | --- | --- |
| PSICU 17 | 64.291 | 40 | 0.020 | 0.009 |
| PSICU 13 | 45.869 | 42 | 0.008 | 0.315 |
| PSICU 21 | 115.855 | 43 | 0.033 | 0.000 |
| PSICU 38 | 54.389 | 42 | 0.014 | 0.095 |
| PSICU 3 | 61.615 | 43 | 0.017 | 0.033 |

### 2.6.3 Charts of item characteristics

### 2.6.4 Model and item reliability

##
## Marginal empirical reliability: 0.756

## 2.7 IRT for factor 6 of the 6-factor model

### 2.7.1 IRT model fitting and comparison

##
##
## == IRT MODEL COMPARISON ==

|  | Modèle | G2 | AIC | BIC | LogLik |
| --- | --- | --- | --- | --- | --- |
| PCM | PCM | 1141.31 | 18748.35 | 18839.67 | -9357.17 |
| Graded | Graded | 995.00 | 18608.04 | 18715.48 | -9284.02 |
| RSM | RSM | 1558.21 | 19147.25 | 19190.23 | -9565.63 |
| GPCM | GPCM | 1069.91 | 18682.96 | 18790.40 | -9321.48 |

### 2.7.2 Evaluation of the selected model

##
##
## == ITEM PARAMETERS ( Graded ) ==

|  | a | b1 | b2 | b3 | b4 |
| --- | --- | --- | --- | --- | --- |
| PSICU 32 | 2.390 | -1.340 | -0.625 | 0.364 | 1.303 |
| PSICU 8 | 1.566 | -1.063 | -0.539 | 0.619 | 1.781 |
| PSICU 15 | 1.268 | -0.856 | -0.212 | 0.605 | 1.597 |
| PSICU 6 | 1.170 | -1.921 | -0.188 | 1.082 | 2.460 |

##
##
## == ASSESSMENT OF MODEL FIT ==

| item | S_X2 | df.S_X2 | RMSEA.S_X2 | p.S_X2 |
| --- | --- | --- | --- | --- |
| PSICU 32 | 46.757 | 25 | 0.023 | 0.005 |
| PSICU 8 | 69.275 | 29 | 0.030 | 0.000 |
| PSICU 15 | 34.911 | 30 | 0.010 | 0.246 |
| PSICU 6 | 54.489 | 30 | 0.023 | 0.004 |

### 2.7.3 Charts of item characteristics

### 2.7.4 Model and item reliability

##
## Marginal empirical reliability: 0.752

# 3 Six-factor model with 31 items (model #9) estimated on the sample of nursing auxiliaries

## 3.1 IRT for the entire scale (31 items)

### 3.1.1 IRT model fitting and comparison

##
##
## == IRT MODEL COMPARISON ==

|  | Modèle | G2 | AIC | BIC | LogLik |
| --- | --- | --- | --- | --- | --- |
| PCM | PCM | 42628.31 | 50673.08 | 51224.35 | -25211.54 |
| Graded | Graded | 42260.85 | 50365.63 | 51049.20 | -25027.81 |
| RSM | RSM | 44985.79 | 52850.57 | 53004.92 | -26390.28 |
| GPCM | GPCM | 42352.39 | 50457.16 | 51140.74 | -25073.58 |

### 3.1.2 Evaluation of the selected model

##
##
## == ITEM PARAMETERS ( Graded ) ==

|  | a | b1 | b2 | b3 | b4 |
| --- | --- | --- | --- | --- | --- |
| PSICU 42 | 1.487 | -1.711 | 0.178 | 1.805 | 2.788 |
| PSICU 27 | 1.697 | -2.302 | -0.347 | 1.085 | 2.348 |
| PSICU 10 | 1.661 | -1.531 | -0.390 | 0.907 | 2.137 |
| PSICU 4 | 1.317 | -1.007 | -0.579 | 0.801 | 2.472 |
| PSICU 9 | 1.478 | -1.040 | -0.103 | 1.278 | 2.439 |
| PSICU 35 | 1.443 | -1.332 | -0.245 | 0.975 | 2.116 |
| PSICU 41 | 1.151 | -2.666 | 1.360 | 2.417 | 3.430 |
| PSICU 26 | 0.965 | -1.956 | 0.822 | 2.637 | 3.894 |
| PSICU 34 | 1.083 | -1.609 | -0.594 | 0.793 | 2.306 |
| PSICU 28 | 1.545 | -2.164 | -0.499 | 0.687 | 1.665 |
| PSICU 29 | 1.052 | -1.758 | -0.734 | 0.741 | 2.296 |
| PSICU 7 | 1.533 | -4.098 | -1.741 | -0.161 | 1.377 |
| PSICU 45 | 0.883 | -1.742 | -0.897 | 1.082 | 2.962 |
| PSICU 5 | 1.267 | -2.892 | -1.256 | 0.203 | 1.523 |
| PSICU 18 | 1.054 | -4.324 | -1.311 | 0.524 | 2.110 |
| PSICU 40 | 1.355 | -3.224 | -0.477 | 0.897 | 2.379 |
| PSICU 37 | 0.950 | -1.565 | -0.790 | 0.812 | 2.290 |
| PSICU 16 | 1.122 | -2.036 | -1.260 | 0.303 | 1.678 |
| PSICU 25 | 1.437 | -3.236 | -1.136 | 0.335 | 1.859 |
| PSICU 50 | 0.833 | -2.363 | 0.181 | 1.462 | 2.362 |
| PSICU 20 | 1.237 | -1.901 | -0.355 | 0.765 | 1.668 |
| PSICU 23 | 1.296 | -2.782 | 0.074 | 1.253 | 2.141 |
| PSICU 17 | 1.288 | -2.018 | -0.627 | 0.496 | 1.345 |
| PSICU 13 | 1.137 | -1.008 | -0.228 | 1.012 | 2.214 |
| PSICU 21 | 0.985 | -0.857 | -0.087 | 1.100 | 2.259 |
| PSICU 38 | 1.169 | -1.230 | -0.375 | 0.698 | 1.803 |
| PSICU 3 | 1.359 | -1.840 | -0.690 | 0.465 | 1.551 |
| PSICU 32 | 1.451 | -2.030 | -1.015 | 0.205 | 1.178 |
| PSICU 8 | 1.291 | -1.248 | -0.508 | 0.774 | 2.057 |
| PSICU 15 | 1.261 | -0.752 | -0.130 | 0.638 | 1.463 |
| PSICU 6 | 1.193 | -1.317 | 0.207 | 1.366 | 2.549 |

##
##
## == ASSESSMENT OF MODEL FIT ==

| item | S_X2 | df.S_X2 | RMSEA.S_X2 | p.S_X2 |
| --- | --- | --- | --- | --- |
| PSICU 42 | 95.680 | 108 | 0.000 | 0.796 |
| PSICU 27 | 108.433 | 100 | 0.012 | 0.265 |
| PSICU 10 | 131.937 | 124 | 0.010 | 0.296 |
| PSICU 4 | 136.861 | 129 | 0.010 | 0.301 |
| PSICU 9 | 167.793 | 135 | 0.020 | 0.029 |
| PSICU 35 | 173.499 | 143 | 0.019 | 0.042 |
| PSICU 41 | 76.175 | 84 | 0.000 | 0.716 |
| PSICU 26 | 127.653 | 113 | 0.015 | 0.164 |
| PSICU 34 | 169.302 | 168 | 0.004 | 0.457 |
| PSICU 28 | 147.522 | 134 | 0.013 | 0.200 |
| PSICU 29 | 170.918 | 163 | 0.009 | 0.320 |
| PSICU 7 | 103.905 | 114 | 0.000 | 0.741 |
| PSICU 45 | 228.127 | 157 | 0.027 | 0.000 |
| PSICU 5 | 140.351 | 141 | 0.000 | 0.500 |
| PSICU 18 | 150.842 | 137 | 0.013 | 0.198 |
| PSICU 40 | 149.333 | 103 | 0.027 | 0.002 |
| PSICU 37 | 153.624 | 159 | 0.000 | 0.605 |
| PSICU 16 | 166.710 | 152 | 0.013 | 0.196 |
| PSICU 25 | 115.787 | 113 | 0.006 | 0.410 |
| PSICU 50 | 154.774 | 149 | 0.008 | 0.356 |
| PSICU 20 | 147.309 | 161 | 0.000 | 0.773 |
| PSICU 23 | 157.559 | 124 | 0.021 | 0.022 |
| PSICU 17 | 141.088 | 155 | 0.000 | 0.781 |
| PSICU 13 | 198.376 | 161 | 0.020 | 0.024 |
| PSICU 21 | 141.875 | 129 | 0.013 | 0.207 |
| PSICU 38 | 176.708 | 155 | 0.015 | 0.112 |
| PSICU 3 | 154.637 | 154 | 0.003 | 0.470 |
| PSICU 32 | 121.360 | 145 | 0.000 | 0.924 |
| PSICU 8 | 144.625 | 159 | 0.000 | 0.786 |
| PSICU 15 | 190.777 | 160 | 0.018 | 0.049 |
| PSICU 6 | 171.105 | 148 | 0.016 | 0.094 |

### 3.1.3 Charts of item characteristics

### 3.1.4 Model and item reliability

##
## Marginal empirical reliability: 0.935

## 3.2 IRT for factor 1 of the 6-factor model

### 3.2.1 IRT model fitting and comparison

##
##
## == IRT MODEL COMPARISON ==

|  | Modèle | G2 | AIC | BIC | LogLik |
| --- | --- | --- | --- | --- | --- |
| PCM | PCM | 4487.40 | 12107.62 | 12253.15 | -6020.81 |
| Graded | Graded | 4404.34 | 12038.56 | 12214.97 | -5979.28 |
| RSM | RSM | 5238.35 | 12816.57 | 12869.49 | -6396.28 |
| GPCM | GPCM | 4439.80 | 12074.02 | 12250.42 | -5997.01 |

### 3.2.2 Evaluation of the selected model

##
##
## == ITEM PARAMETERS ( Graded ) ==

|  | a | b1 | b2 | b3 | b4 |
| --- | --- | --- | --- | --- | --- |
| PSICU 42 | 1.627 | -1.609 | 0.200 | 1.735 | 2.641 |
| PSICU 27 | 2.039 | -2.108 | -0.293 | 1.023 | 2.145 |
| PSICU 10 | 2.009 | -1.376 | -0.318 | 0.853 | 1.947 |
| PSICU 4 | 1.524 | -0.904 | -0.506 | 0.752 | 2.260 |
| PSICU 9 | 1.684 | -0.946 | -0.065 | 1.192 | 2.253 |
| PSICU 35 | 1.528 | -1.261 | -0.194 | 0.964 | 2.042 |
| PSICU 41 | 1.266 | -2.507 | 1.282 | 2.270 | 3.204 |
| PSICU 26 | 1.139 | -1.732 | 0.745 | 2.355 | 3.436 |

##
##
## == ASSESSMENT OF MODEL FIT ==

| item | S_X2 | df.S_X2 | RMSEA.S_X2 | p.S_X2 |
| --- | --- | --- | --- | --- |
| PSICU 42 | 67.968 | 46 | 0.028 | 0.019 |
| PSICU 27 | 48.411 | 44 | 0.013 | 0.299 |
| PSICU 10 | 47.547 | 49 | 0.000 | 0.532 |
| PSICU 4 | 81.772 | 55 | 0.028 | 0.011 |
| PSICU 9 | 63.034 | 54 | 0.017 | 0.187 |
| PSICU 35 | 45.208 | 56 | 0.000 | 0.848 |
| PSICU 41 | 40.333 | 49 | 0.000 | 0.806 |
| PSICU 26 | 65.217 | 53 | 0.019 | 0.121 |

### 3.2.3 Charts of item characteristics

### 3.2.4 Model and item reliability

##
## Marginal empirical reliability: 0.849

## 3.3 IRT for factor 2 of the 6-factor model

### 3.3.1 IRT model fitting and comparison

##
##
## == IRT MODEL COMPARISON ==

|  | Modèle | G2 | AIC | BIC | LogLik |
| --- | --- | --- | --- | --- | --- |
| PCM | PCM | 2798.56 | 10243.68 | 10353.93 | -5096.84 |
| Graded | Graded | 2664.22 | 10119.33 | 10251.64 | -5029.67 |
| RSM | RSM | 2979.46 | 10394.57 | 10438.68 | -5187.29 |
| GPCM | GPCM | 2720.68 | 10175.80 | 10308.11 | -5057.90 |

### 3.3.2 Evaluation of the selected model

##
##
## == ITEM PARAMETERS ( Graded ) ==

|  | a | b1 | b2 | b3 | b4 |
| --- | --- | --- | --- | --- | --- |
| PSICU 34 | 1.385 | -1.398 | -0.535 | 0.685 | 1.984 |
| PSICU 28 | 2.003 | -1.916 | -0.446 | 0.633 | 1.498 |
| PSICU 29 | 1.264 | -1.566 | -0.658 | 0.667 | 2.042 |
| PSICU 7 | 1.927 | -3.524 | -1.555 | -0.147 | 1.238 |
| PSICU 45 | 1.037 | -1.551 | -0.792 | 0.982 | 2.623 |
| PSICU 5 | 1.464 | -2.631 | -1.150 | 0.200 | 1.414 |

##
##
## == ASSESSMENT OF MODEL FIT ==

| item | S_X2 | df.S_X2 | RMSEA.S_X2 | p.S_X2 |
| --- | --- | --- | --- | --- |
| PSICU 34 | 88.732 | 45 | 0.040 | 0.000 |
| PSICU 28 | 46.553 | 38 | 0.019 | 0.161 |
| PSICU 29 | 53.387 | 45 | 0.018 | 0.183 |
| PSICU 7 | 19.726 | 30 | 0.000 | 0.923 |
| PSICU 45 | 86.279 | 47 | 0.037 | 0.000 |
| PSICU 5 | 47.514 | 43 | 0.013 | 0.294 |

### 3.3.3 Charts of item characteristics

### 3.3.4 Model and item reliability

##
## Marginal empirical reliability: 0.8

## 3.4 IRT for factor 3 of the 6-factor model

### 3.4.1 IRT model fitting and comparison

##
##
## == IRT MODEL COMPARISON ==

|  | Modèle | G2 | AIC | BIC | LogLik |
| --- | --- | --- | --- | --- | --- |
| PCM | PCM | 1276.06 | 8106.14 | 8198.76 | -4032.07 |
| Graded | Graded | 1208.08 | 8046.17 | 8156.42 | -3998.08 |
| RSM | RSM | 1619.36 | 8425.44 | 8465.14 | -4203.72 |
| GPCM | GPCM | 1249.51 | 8087.60 | 8197.85 | -4018.80 |

### 3.4.2 Evaluation of the selected model

##
##
## == ITEM PARAMETERS ( Graded ) ==

|  | a | b1 | b2 | b3 | b4 |
| --- | --- | --- | --- | --- | --- |
| PSICU 18 | 2.214 | -2.721 | -0.908 | 0.351 | 1.421 |
| PSICU 40 | 1.679 | -2.876 | -0.425 | 0.840 | 2.114 |
| PSICU 37 | 1.400 | -1.247 | -0.651 | 0.624 | 1.770 |
| PSICU 16 | 1.751 | -1.588 | -0.991 | 0.244 | 1.312 |
| PSICU 25 | 1.478 | -3.215 | -1.146 | 0.348 | 1.844 |

##
##
## == ASSESSMENT OF MODEL FIT ==

| item | S_X2 | df.S_X2 | RMSEA.S_X2 | p.S_X2 |
| --- | --- | --- | --- | --- |
| PSICU 18 | 14.634 | 25 | 0.000 | 0.950 |
| PSICU 40 | 55.169 | 27 | 0.041 | 0.001 |
| PSICU 37 | 68.641 | 35 | 0.040 | 0.001 |
| PSICU 16 | 33.469 | 32 | 0.009 | 0.396 |
| PSICU 25 | 35.139 | 30 | 0.017 | 0.238 |

### 3.4.3 Charts of item characteristics

### 3.4.4 Model and item reliability

##
## Marginal empirical reliability: 0.8

## 3.5 IRT for factor 4 of the 6-factor model

### 3.5.1 IRT model fitting and comparison

##
##
## == IRT MODEL COMPARISON ==

|  | Modèle | G2 | AIC | BIC | LogLik |
| --- | --- | --- | --- | --- | --- |
| PCM | PCM | 182.02 | 5080.23 | 5137.57 | -2527.12 |
| Graded | Graded | 167.20 | 5069.41 | 5135.57 | -2519.71 |
| RSM | RSM | 263.47 | 5149.69 | 5180.56 | -2567.84 |
| GPCM | GPCM | 163.63 | 5065.85 | 5132.00 | -2517.92 |

### 3.5.2 Evaluation of the selected model

##
##
## == ITEM PARAMETERS ( GPCM ) ==

|  | a | b1 | b2 | b3 | b4 |
| --- | --- | --- | --- | --- | --- |
| PSICU 50 | 0.664 | -1.971 | 0.881 | 1.388 | 0.264 |
| PSICU 20 | 2.121 | -1.281 | -0.183 | 0.582 | 0.955 |
| PSICU 23 | 0.719 | -3.807 | 0.802 | 1.469 | 1.058 |

##
##
## == ASSESSMENT OF MODEL FIT ==

| item | S_X2 | df.S_X2 | RMSEA.S_X2 | p.S_X2 |
| --- | --- | --- | --- | --- |
| PSICU 50 | 16.355 | 11 | 0.028 | 0.128 |
| PSICU 20 | 30.280 | 11 | 0.054 | 0.001 |
| PSICU 23 | 20.911 | 10 | 0.042 | 0.022 |

### 3.5.3 Charts of item characteristics

### 3.5.4 Model and item reliability

##
## Marginal empirical reliability: 0.735

## 3.6 IRT for factor 5 of the 6-factor model

### 3.6.1 IRT model fitting and comparison

##
##
## == IRT MODEL COMPARISON ==

|  | Modèle | G2 | AIC | BIC | LogLik |
| --- | --- | --- | --- | --- | --- |
| PCM | PCM | 1727.08 | 8788.59 | 8881.20 | -4373.30 |
| Graded | Graded | 1648.30 | 8717.81 | 8828.06 | -4333.91 |
| RSM | RSM | 1814.78 | 8852.29 | 8891.98 | -4417.15 |
| GPCM | GPCM | 1683.73 | 8753.24 | 8863.49 | -4351.62 |

### 3.6.2 Evaluation of the selected model

##
##
## == ITEM PARAMETERS ( Graded ) ==

|  | a | b1 | b2 | b3 | b4 |
| --- | --- | --- | --- | --- | --- |
| PSICU 17 | 2.617 | -1.438 | -0.433 | 0.376 | 0.985 |
| PSICU 13 | 1.872 | -0.748 | -0.150 | 0.784 | 1.681 |
| PSICU 21 | 1.930 | -0.587 | -0.074 | 0.740 | 1.528 |
| PSICU 38 | 1.580 | -1.007 | -0.291 | 0.610 | 1.534 |
| PSICU 3 | 1.188 | -1.995 | -0.722 | 0.515 | 1.689 |

##
##
## == ASSESSMENT OF MODEL FIT ==

| item | S_X2 | df.S_X2 | RMSEA.S_X2 | p.S_X2 |
| --- | --- | --- | --- | --- |
| PSICU 17 | 25.611 | 31 | 0.000 | 0.740 |
| PSICU 13 | 53.279 | 38 | 0.026 | 0.051 |
| PSICU 21 | 80.901 | 37 | 0.044 | 0.000 |
| PSICU 38 | 63.867 | 40 | 0.031 | 0.010 |
| PSICU 3 | 36.124 | 42 | 0.000 | 0.726 |

### 3.6.3 Charts of item characteristics

### 3.6.4 Model and item reliability

##
## Marginal empirical reliability: 0.822

## 3.7 IRT for factor 6 of the 6-factor model

### 3.7.1 IRT model fitting and comparison

##
##
## == IRT MODEL COMPARISON ==

|  | Modèle | G2 | AIC | BIC | LogLik |
| --- | --- | --- | --- | --- | --- |
| PCM | PCM | 616.53 | 7097.30 | 7172.27 | -3531.65 |
| Graded | Graded | 593.29 | 7080.06 | 7168.26 | -3520.03 |
| RSM | RSM | 756.62 | 7219.38 | 7254.66 | -3601.69 |
| GPCM | GPCM | 606.91 | 7093.67 | 7181.88 | -3526.84 |

### 3.7.2 Evaluation of the selected model

##
##
## == ITEM PARAMETERS ( Graded ) ==

|  | a | b1 | b2 | b3 | b4 |
| --- | --- | --- | --- | --- | --- |
| PSICU 32 | 1.806 | -1.793 | -0.866 | 0.217 | 1.067 |
| PSICU 8 | 1.570 | -1.098 | -0.423 | 0.718 | 1.837 |
| PSICU 15 | 1.447 | -0.667 | -0.089 | 0.617 | 1.368 |
| PSICU 6 | 1.549 | -1.116 | 0.197 | 1.192 | 2.191 |

##
##
## == ASSESSMENT OF MODEL FIT ==

| item | S_X2 | df.S_X2 | RMSEA.S_X2 | p.S_X2 |
| --- | --- | --- | --- | --- |
| PSICU 32 | 22.603 | 23 | 0.000 | 0.484 |
| PSICU 8 | 32.626 | 27 | 0.019 | 0.210 |
| PSICU 15 | 35.900 | 28 | 0.022 | 0.145 |
| PSICU 6 | 30.552 | 25 | 0.019 | 0.204 |

### 3.7.3 Charts of item characteristics

### 3.7.4 Model and item reliability

##
## Marginal empirical reliability: 0.74

# 4 Six-factor model with 26 items (model #11) estimated on the original population of the scale (nurses, physicians and medical residents)

## 4.1 IRT for the entire scale (26 items)

### 4.1.1 IRT model fitting and comparison

##
##
## == IRT MODEL COMPARISON ==

|  | Modèle | G2 | AIC | BIC | LogLik |
| --- | --- | --- | --- | --- | --- |
| PCM | PCM | 86077.71 | 109745.8 | 110309.9 | -54767.90 |
| Graded | Graded | 85350.39 | 109068.5 | 109766.9 | -54404.24 |
| RSM | RSM | 92534.40 | 116052.5 | 116213.6 | -57996.24 |
| GPCM | GPCM | 85670.79 | 109388.9 | 110087.2 | -54564.43 |

### 4.1.2 Evaluation of the selected model

##
##
## == ITEM PARAMETERS ( Graded ) ==

|  | a | b1 | b2 | b3 | b4 |
| --- | --- | --- | --- | --- | --- |
| PSICU 42 | 1.288 | -3.212 | -0.173 | 1.881 | 3.503 |
| PSICU 27 | 1.426 | -2.937 | -0.483 | 1.334 | 2.991 |
| PSICU 10 | 1.224 | -2.141 | -0.869 | 0.903 | 2.751 |
| PSICU 35 | 1.382 | -1.474 | -0.638 | 0.789 | 2.276 |
| PSICU 41 | 0.812 | -4.771 | 1.991 | 3.946 | 5.565 |
| PSICU 26 | 1.035 | -1.915 | 0.394 | 2.074 | 3.523 |
| PSICU 34 | 1.148 | -0.926 | -0.096 | 1.243 | 2.652 |
| PSICU 28 | 1.296 | -2.211 | -0.283 | 1.076 | 2.563 |
| PSICU 29 | 1.160 | -1.340 | -0.645 | 0.729 | 2.041 |
| PSICU 7 | 1.362 | -4.999 | -1.679 | 0.214 | 1.903 |
| PSICU 5 | 1.137 | -3.151 | -1.749 | -0.054 | 1.545 |
| PSICU 18 | 0.987 | -5.561 | -1.636 | 0.521 | 2.274 |
| PSICU 40 | 1.297 | -4.642 | -0.971 | 0.797 | 2.376 |
| PSICU 37 | 1.157 | -2.286 | -1.723 | -0.116 | 1.481 |
| PSICU 16 | 1.177 | -3.227 | -2.280 | -0.493 | 0.938 |
| PSICU 50 | 0.941 | -3.662 | -0.599 | 1.022 | 2.275 |
| PSICU 20 | 1.174 | -2.428 | -0.886 | 0.475 | 1.745 |
| PSICU 23 | 1.202 | -2.468 | -0.116 | 1.405 | 2.634 |
| PSICU 17 | 0.949 | -1.663 | -0.501 | 0.791 | 2.160 |
| PSICU 13 | 0.986 | -1.361 | -0.563 | 0.984 | 2.421 |
| PSICU 21 | 0.809 | -0.827 | -0.146 | 1.472 | 3.043 |
| PSICU 38 | 1.099 | -1.891 | -0.986 | 0.408 | 1.753 |
| PSICU 32 | 1.305 | -1.830 | -0.870 | 0.461 | 1.726 |
| PSICU 8 | 0.959 | -1.477 | -0.759 | 0.820 | 2.431 |
| PSICU 15 | 0.936 | -1.081 | -0.287 | 0.729 | 1.960 |
| PSICU 6 | 0.935 | -2.275 | -0.238 | 1.268 | 2.892 |

##
##
## == ASSESSMENT OF MODEL FIT ==

| item | S_X2 | df.S_X2 | RMSEA.S_X2 | p.S_X2 |
| --- | --- | --- | --- | --- |
| PSICU 42 | 125.738 | 132 | 0.000 | 0.637 |
| PSICU 27 | 139.294 | 140 | 0.000 | 0.501 |
| PSICU 10 | 202.153 | 175 | 0.010 | 0.078 |
| PSICU 35 | 193.469 | 173 | 0.009 | 0.137 |
| PSICU 41 | 137.898 | 114 | 0.011 | 0.063 |
| PSICU 26 | 181.444 | 171 | 0.006 | 0.278 |
| PSICU 34 | 273.155 | 191 | 0.016 | 0.000 |
| PSICU 28 | 186.369 | 169 | 0.008 | 0.171 |
| PSICU 29 | 219.760 | 191 | 0.010 | 0.075 |
| PSICU 7 | 150.662 | 140 | 0.007 | 0.254 |
| PSICU 5 | 185.175 | 174 | 0.006 | 0.267 |
| PSICU 18 | 180.272 | 155 | 0.010 | 0.081 |
| PSICU 40 | 171.748 | 142 | 0.011 | 0.045 |
| PSICU 37 | 222.898 | 182 | 0.012 | 0.021 |
| PSICU 16 | 201.439 | 169 | 0.011 | 0.045 |
| PSICU 50 | 215.677 | 191 | 0.009 | 0.106 |
| PSICU 20 | 189.426 | 183 | 0.005 | 0.357 |
| PSICU 23 | 173.632 | 178 | 0.000 | 0.578 |
| PSICU 17 | 264.976 | 206 | 0.013 | 0.003 |
| PSICU 13 | 189.283 | 203 | 0.000 | 0.746 |
| PSICU 21 | 238.832 | 205 | 0.010 | 0.053 |
| PSICU 38 | 169.382 | 186 | 0.000 | 0.803 |
| PSICU 32 | 209.250 | 181 | 0.010 | 0.074 |
| PSICU 8 | 248.811 | 199 | 0.013 | 0.009 |
| PSICU 15 | 251.793 | 208 | 0.012 | 0.020 |
| PSICU 6 | 193.971 | 195 | 0.000 | 0.507 |

### 4.1.3 Charts of item characteristics

### 4.1.4 Model and item reliability

##
## Marginal empirical reliability: 0.905

## 4.2 IRT for factor 1 of the 6-factor model

### 4.2.1 IRT model fitting and comparison

##
##
## == IRT MODEL COMPARISON ==

|  | Modèle | G2 | AIC | BIC | LogLik |
| --- | --- | --- | --- | --- | --- |
| PCM | PCM | 8477.45 | 30639.97 | 30817.25 | -15286.98 |
| Graded | Graded | 8209.62 | 30386.15 | 30601.03 | -15153.07 |
| RSM | RSM | 10677.68 | 32798.21 | 32862.67 | -16387.10 |
| GPCM | GPCM | 8382.36 | 30558.89 | 30773.77 | -15239.44 |

### 4.2.2 Evaluation of the selected model

##
##
## == ITEM PARAMETERS ( Graded ) ==

|  | a | b1 | b2 | b3 | b4 |
| --- | --- | --- | --- | --- | --- |
| PSICU 42 | 1.479 | -2.948 | -0.148 | 1.736 | 3.184 |
| PSICU 27 | 1.881 | -2.499 | -0.398 | 1.163 | 2.563 |
| PSICU 10 | 1.800 | -1.713 | -0.688 | 0.748 | 2.192 |
| PSICU 4 | 1.440 | -1.769 | -1.186 | 0.276 | 1.883 |
| PSICU 9 | 1.214 | -1.906 | -0.628 | 1.072 | 2.735 |
| PSICU 35 | 1.693 | -1.304 | -0.545 | 0.729 | 2.026 |
| PSICU 41 | 0.997 | -4.038 | 1.702 | 3.332 | 4.671 |
| PSICU 26 | 1.323 | -1.625 | 0.345 | 1.764 | 2.956 |

##
##
## == ASSESSMENT OF MODEL FIT ==

| item | S_X2 | df.S_X2 | RMSEA.S_X2 | p.S_X2 |
| --- | --- | --- | --- | --- |
| PSICU 42 | 92.616 | 56 | 0.020 | 0.002 |
| PSICU 27 | 50.725 | 53 | 0.000 | 0.563 |
| PSICU 10 | 81.123 | 56 | 0.017 | 0.016 |
| PSICU 4 | 108.014 | 60 | 0.022 | 0.000 |
| PSICU 9 | 73.553 | 65 | 0.009 | 0.218 |
| PSICU 35 | 82.989 | 57 | 0.017 | 0.014 |
| PSICU 41 | 65.486 | 59 | 0.008 | 0.262 |
| PSICU 26 | 98.154 | 64 | 0.018 | 0.004 |

### 4.2.3 Charts of item characteristics

### 4.2.4 Model and item reliability

##
## Marginal empirical reliability: 0.827

## 4.3 IRT for factor 2 of the 6-factor model

### 4.3.1 IRT model fitting and comparison

##
##
## == IRT MODEL COMPARISON ==

|  | Modèle | G2 | AIC | BIC | LogLik |
| --- | --- | --- | --- | --- | --- |
| PCM | PCM | 4370.66 | 25494.00 | 25628.30 | -12722.00 |
| Graded | Graded | 4222.12 | 25355.47 | 25516.63 | -12647.73 |
| RSM | RSM | 5176.79 | 26270.13 | 26323.85 | -13125.07 |
| GPCM | GPCM | 4331.82 | 25465.17 | 25626.33 | -12702.58 |

### 4.3.2 Evaluation of the selected model

##
##
## == ITEM PARAMETERS ( Graded ) ==

|  | a | b1 | b2 | b3 | b4 |
| --- | --- | --- | --- | --- | --- |
| PSICU 34 | 1.567 | -0.780 | -0.096 | 1.031 | 2.209 |
| PSICU 28 | 1.620 | -1.936 | -0.254 | 0.953 | 2.258 |
| PSICU 29 | 1.466 | -1.174 | -0.582 | 0.623 | 1.784 |
| PSICU 7 | 1.692 | -4.254 | -1.488 | 0.184 | 1.704 |
| PSICU 45 | 1.498 | -2.629 | -0.896 | 0.724 | 2.159 |
| PSICU 5 | 1.326 | -2.825 | -1.585 | -0.044 | 1.417 |

##
##
## == ASSESSMENT OF MODEL FIT ==

| item | S_X2 | df.S_X2 | RMSEA.S_X2 | p.S_X2 |
| --- | --- | --- | --- | --- |
| PSICU 34 | 124.214 | 48 | 0.032 | 0.000 |
| PSICU 28 | 73.592 | 46 | 0.019 | 0.006 |
| PSICU 29 | 103.162 | 50 | 0.026 | 0.000 |
| PSICU 7 | 45.923 | 37 | 0.012 | 0.149 |
| PSICU 45 | 52.636 | 47 | 0.009 | 0.265 |
| PSICU 5 | 80.008 | 49 | 0.020 | 0.003 |

### 4.3.3 Charts of item characteristics

### 4.3.4 Model and item reliability

##
## Marginal empirical reliability: 0.796

## 4.4 IRT for factor 3 of the 6-factor model

### 4.4.1 IRT model fitting and comparison

##
##
## == IRT MODEL COMPARISON ==

|  | Modèle | G2 | AIC | BIC | LogLik |
| --- | --- | --- | --- | --- | --- |
| PCM | PCM | 1654.80 | 19439.41 | 19552.22 | -9698.70 |
| Graded | Graded | 1474.71 | 19267.32 | 19401.62 | -9608.66 |
| RSM | RSM | 2356.44 | 20117.05 | 20165.40 | -10049.53 |
| GPCM | GPCM | 1595.08 | 19387.69 | 19521.99 | -9668.84 |

### 4.4.2 Evaluation of the selected model

##
##
## == ITEM PARAMETERS ( Graded ) ==

|  | a | b1 | b2 | b3 | b4 |
| --- | --- | --- | --- | --- | --- |
| PSICU 18 | 2.378 | -3.148 | -1.048 | 0.328 | 1.424 |
| PSICU 40 | 2.086 | -3.471 | -0.783 | 0.639 | 1.859 |
| PSICU 37 | 1.737 | -1.825 | -1.397 | -0.108 | 1.173 |
| PSICU 16 | 1.809 | -2.499 | -1.799 | -0.390 | 0.754 |
| PSICU 25 | 1.639 | -3.058 | -1.364 | 0.172 | 1.672 |

##
##
## == ASSESSMENT OF MODEL FIT ==

| item | S_X2 | df.S_X2 | RMSEA.S_X2 | p.S_X2 |
| --- | --- | --- | --- | --- |
| PSICU 18 | 26.040 | 26 | 0.001 | 0.461 |
| PSICU 40 | 33.185 | 23 | 0.017 | 0.078 |
| PSICU 37 | 77.434 | 34 | 0.028 | 0.000 |
| PSICU 16 | 44.010 | 33 | 0.014 | 0.095 |
| PSICU 25 | 56.174 | 31 | 0.023 | 0.004 |

### 4.4.3 Charts of item characteristics

### 4.4.4 Model and item reliability

##
## Marginal empirical reliability: 0.827

## 4.5 IRT for factor 4 of the 6-factor model

### 4.5.1 IRT model fitting and comparison

##
##
## == IRT MODEL COMPARISON ==

|  | Modèle | G2 | AIC | BIC | LogLik |
| --- | --- | --- | --- | --- | --- |
| PCM | PCM | 400.72 | 13121.54 | 13191.38 | -6547.77 |
| Graded | Graded | 323.49 | 13048.31 | 13128.89 | -6509.15 |
| RSM | RSM | 501.11 | 13209.93 | 13247.53 | -6597.96 |
| GPCM | GPCM | 330.19 | 13055.00 | 13135.58 | -6512.50 |

### 4.5.2 Evaluation of the selected model

##
##
## == ITEM PARAMETERS ( Graded ) ==

|  | a | b1 | b2 | b3 | b4 |
| --- | --- | --- | --- | --- | --- |
| PSICU 50 | 1.459 | -2.657 | -0.451 | 0.778 | 1.730 |
| PSICU 20 | 3.399 | -1.513 | -0.600 | 0.291 | 1.128 |
| PSICU 23 | 1.215 | -2.454 | -0.130 | 1.375 | 2.596 |

##
##
## == ASSESSMENT OF MODEL FIT ==

| item | S_X2 | df.S_X2 | RMSEA.S_X2 | p.S_X2 |
| --- | --- | --- | --- | --- |
| PSICU 50 | 45.010 | 12 | 0.042 | 0.000 |
| PSICU 20 | 26.941 | 10 | 0.033 | 0.003 |
| PSICU 23 | 41.095 | 13 | 0.037 | 0.000 |

### 4.5.3 Charts of item characteristics

### 4.5.4 Model and item reliability

##
## Marginal empirical reliability: 0.782

## 4.6 IRT for factor 5 of the 6-factor model

### 4.6.1 IRT model fitting and comparison

##
##
## == IRT MODEL COMPARISON ==

|  | Modèle | G2 | AIC | BIC | LogLik |
| --- | --- | --- | --- | --- | --- |
| PCM | PCM | 2860.15 | 23285.86 | 23398.67 | -11621.93 |
| Graded | Graded | 2751.02 | 23184.72 | 23319.02 | -11567.36 |
| RSM | RSM | 3083.32 | 23485.03 | 23533.38 | -11733.51 |
| GPCM | GPCM | 2821.45 | 23255.15 | 23389.46 | -11602.58 |

### 4.6.2 Evaluation of the selected model

##
##
## == ITEM PARAMETERS ( Graded ) ==

|  | a | b1 | b2 | b3 | b4 |
| --- | --- | --- | --- | --- | --- |
| PSICU 17 | 1.858 | -1.101 | -0.351 | 0.514 | 1.452 |
| PSICU 13 | 1.599 | -1.006 | -0.429 | 0.716 | 1.790 |
| PSICU 21 | 1.265 | -0.609 | -0.122 | 1.051 | 2.200 |
| PSICU 38 | 1.386 | -1.602 | -0.836 | 0.346 | 1.515 |
| PSICU 3 | 1.194 | -2.484 | -1.027 | 0.356 | 1.819 |

##
##
## == ASSESSMENT OF MODEL FIT ==

| item | S_X2 | df.S_X2 | RMSEA.S_X2 | p.S_X2 |
| --- | --- | --- | --- | --- |
| PSICU 17 | 64.291 | 40 | 0.020 | 0.009 |
| PSICU 13 | 45.869 | 42 | 0.008 | 0.315 |
| PSICU 21 | 115.855 | 43 | 0.033 | 0.000 |
| PSICU 38 | 54.389 | 42 | 0.014 | 0.095 |
| PSICU 3 | 61.615 | 43 | 0.017 | 0.033 |

### 4.6.3 Charts of item characteristics

### 4.6.4 Model and item reliability

##
## Marginal empirical reliability: 0.756

## 4.7 IRT for factor 6 of the 6-factor model

### 4.7.1 IRT model fitting and comparison

##
##
## == IRT MODEL COMPARISON ==

|  | Modèle | G2 | AIC | BIC | LogLik |
| --- | --- | --- | --- | --- | --- |
| PCM | PCM | 1141.31 | 18748.35 | 18839.67 | -9357.17 |
| Graded | Graded | 995.00 | 18608.04 | 18715.48 | -9284.02 |
| RSM | RSM | 1558.21 | 19147.25 | 19190.23 | -9565.63 |
| GPCM | GPCM | 1069.91 | 18682.96 | 18790.40 | -9321.48 |

### 4.7.2 Evaluation of the selected model

##
##
## == ITEM PARAMETERS ( Graded ) ==

|  | a | b1 | b2 | b3 | b4 |
| --- | --- | --- | --- | --- | --- |
| PSICU 32 | 2.390 | -1.340 | -0.625 | 0.364 | 1.303 |
| PSICU 8 | 1.566 | -1.063 | -0.539 | 0.619 | 1.781 |
| PSICU 15 | 1.268 | -0.856 | -0.212 | 0.605 | 1.597 |
| PSICU 6 | 1.170 | -1.921 | -0.188 | 1.082 | 2.460 |

##
##
## == ASSESSMENT OF MODEL FIT ==

| item | S_X2 | df.S_X2 | RMSEA.S_X2 | p.S_X2 |
| --- | --- | --- | --- | --- |
| PSICU 32 | 46.757 | 25 | 0.023 | 0.005 |
| PSICU 8 | 69.275 | 29 | 0.030 | 0.000 |
| PSICU 15 | 34.911 | 30 | 0.010 | 0.246 |
| PSICU 6 | 54.489 | 30 | 0.023 | 0.004 |

### 4.7.3 Charts of item characteristics

### 4.7.4 Model and item reliability

##
## Marginal empirical reliability: 0.752

# 5 Six-factor model with 26 items (model #11) estimated on the sample of nursing auxiliaries

## 5.1 IRT for the entire scale (26 items)

### 5.1.1 IRT model fitting and comparison

##
##
## == IRT MODEL COMPARISON ==

|  | Modèle | G2 | AIC | BIC | LogLik |
| --- | --- | --- | --- | --- | --- |
| PCM | PCM | 34516.80 | 42521.57 | 42984.64 | -21155.79 |
| Graded | Graded | 34268.42 | 42323.19 | 42896.51 | -21031.60 |
| RSM | RSM | 36533.04 | 44387.81 | 44520.11 | -22163.90 |
| GPCM | GPCM | 34317.32 | 42372.09 | 42945.42 | -21056.05 |

### 5.1.2 Evaluation of the selected model

##
##
## == ITEM PARAMETERS ( Graded ) ==

|  | a | b1 | b2 | b3 | b4 |
| --- | --- | --- | --- | --- | --- |
| PSICU 42 | 1.458 | -1.732 | 0.181 | 1.830 | 2.818 |
| PSICU 27 | 1.670 | -2.330 | -0.352 | 1.100 | 2.368 |
| PSICU 10 | 1.574 | -1.580 | -0.397 | 0.938 | 2.200 |
| PSICU 35 | 1.400 | -1.359 | -0.249 | 0.996 | 2.153 |
| PSICU 41 | 1.134 | -2.693 | 1.376 | 2.444 | 3.464 |
| PSICU 26 | 0.959 | -1.968 | 0.823 | 2.649 | 3.915 |
| PSICU 34 | 1.033 | -1.665 | -0.613 | 0.824 | 2.387 |
| PSICU 28 | 1.537 | -2.175 | -0.505 | 0.689 | 1.672 |
| PSICU 29 | 1.063 | -1.750 | -0.734 | 0.736 | 2.282 |
| PSICU 7 | 1.530 | -4.102 | -1.747 | -0.159 | 1.381 |
| PSICU 5 | 1.246 | -2.924 | -1.269 | 0.207 | 1.543 |
| PSICU 18 | 1.099 | -4.185 | -1.278 | 0.510 | 2.052 |
| PSICU 40 | 1.388 | -3.171 | -0.469 | 0.886 | 2.347 |
| PSICU 37 | 0.997 | -1.519 | -0.771 | 0.782 | 2.210 |
| PSICU 16 | 1.195 | -1.951 | -1.209 | 0.290 | 1.609 |
| PSICU 50 | 0.857 | -2.312 | 0.174 | 1.428 | 2.310 |
| PSICU 20 | 1.245 | -1.897 | -0.357 | 0.759 | 1.661 |
| PSICU 23 | 1.308 | -2.763 | 0.073 | 1.248 | 2.132 |
| PSICU 17 | 1.319 | -1.992 | -0.624 | 0.489 | 1.330 |
| PSICU 13 | 1.182 | -0.982 | -0.220 | 0.990 | 2.161 |
| PSICU 21 | 0.999 | -0.852 | -0.088 | 1.091 | 2.239 |
| PSICU 38 | 1.233 | -1.193 | -0.368 | 0.673 | 1.743 |
| PSICU 32 | 1.441 | -2.040 | -1.018 | 0.208 | 1.186 |
| PSICU 8 | 1.220 | -1.291 | -0.520 | 0.808 | 2.135 |
| PSICU 15 | 1.216 | -0.771 | -0.134 | 0.652 | 1.497 |
| PSICU 6 | 1.113 | -1.381 | 0.219 | 1.435 | 2.675 |

##
##
## == ASSESSMENT OF MODEL FIT ==

| item | S_X2 | df.S_X2 | RMSEA.S_X2 | p.S_X2 |
| --- | --- | --- | --- | --- |
| PSICU 42 | 81.605 | 96 | 0.000 | 0.852 |
| PSICU 27 | 105.493 | 89 | 0.017 | 0.112 |
| PSICU 10 | 142.860 | 117 | 0.019 | 0.052 |
| PSICU 35 | 164.596 | 132 | 0.020 | 0.029 |
| PSICU 41 | 101.256 | 82 | 0.020 | 0.073 |
| PSICU 26 | 112.544 | 109 | 0.007 | 0.389 |
| PSICU 34 | 143.113 | 139 | 0.007 | 0.388 |
| PSICU 28 | 144.204 | 127 | 0.015 | 0.141 |
| PSICU 29 | 187.222 | 141 | 0.023 | 0.006 |
| PSICU 7 | 90.534 | 94 | 0.000 | 0.582 |
| PSICU 5 | 109.161 | 116 | 0.000 | 0.661 |
| PSICU 18 | 139.199 | 117 | 0.018 | 0.079 |
| PSICU 40 | 101.095 | 95 | 0.010 | 0.315 |
| PSICU 37 | 104.788 | 99 | 0.010 | 0.326 |
| PSICU 16 | 125.440 | 130 | 0.000 | 0.597 |
| PSICU 50 | 94.614 | 78 | 0.019 | 0.097 |
| PSICU 20 | 118.424 | 121 | 0.000 | 0.549 |
| PSICU 23 | 111.543 | 121 | 0.000 | 0.720 |
| PSICU 17 | 150.819 | 134 | 0.014 | 0.152 |
| PSICU 13 | 132.029 | 130 | 0.005 | 0.434 |
| PSICU 21 | 138.526 | 125 | 0.013 | 0.193 |
| PSICU 38 | 105.871 | 105 | 0.004 | 0.458 |
| PSICU 32 | 148.047 | 127 | 0.017 | 0.098 |
| PSICU 8 | 165.502 | 134 | 0.020 | 0.034 |
| PSICU 15 | 165.559 | 138 | 0.018 | 0.055 |
| PSICU 6 | 135.462 | 129 | 0.009 | 0.331 |

### 5.1.3 Charts of item characteristics

### 5.1.4 Model and item reliability

##
## Marginal empirical reliability: 0.922

## 5.2 IRT for factor 1 of the 6-factor model

### 5.2.1 IRT model fitting and comparison

##
##
## == IRT MODEL COMPARISON ==

|  | Modèle | G2 | AIC | BIC | LogLik |
| --- | --- | --- | --- | --- | --- |
| PCM | PCM | 4487.40 | 12107.62 | 12253.15 | -6020.81 |
| Graded | Graded | 4404.34 | 12038.56 | 12214.97 | -5979.28 |
| RSM | RSM | 5238.35 | 12816.57 | 12869.49 | -6396.28 |
| GPCM | GPCM | 4439.80 | 12074.02 | 12250.42 | -5997.01 |

### 5.2.2 Evaluation of the selected model

##
##
## == ITEM PARAMETERS ( Graded ) ==

|  | a | b1 | b2 | b3 | b4 |
| --- | --- | --- | --- | --- | --- |
| PSICU 42 | 1.627 | -1.609 | 0.200 | 1.735 | 2.641 |
| PSICU 27 | 2.039 | -2.108 | -0.293 | 1.023 | 2.145 |
| PSICU 10 | 2.009 | -1.376 | -0.318 | 0.853 | 1.947 |
| PSICU 4 | 1.524 | -0.904 | -0.506 | 0.752 | 2.260 |
| PSICU 9 | 1.684 | -0.946 | -0.065 | 1.192 | 2.253 |
| PSICU 35 | 1.528 | -1.261 | -0.194 | 0.964 | 2.042 |
| PSICU 41 | 1.266 | -2.507 | 1.282 | 2.270 | 3.204 |
| PSICU 26 | 1.139 | -1.732 | 0.745 | 2.355 | 3.436 |

##
##
## == ASSESSMENT OF MODEL FIT ==

| item | S_X2 | df.S_X2 | RMSEA.S_X2 | p.S_X2 |
| --- | --- | --- | --- | --- |
| PSICU 42 | 67.968 | 46 | 0.028 | 0.019 |
| PSICU 27 | 48.411 | 44 | 0.013 | 0.299 |
| PSICU 10 | 47.547 | 49 | 0.000 | 0.532 |
| PSICU 4 | 81.772 | 55 | 0.028 | 0.011 |
| PSICU 9 | 63.034 | 54 | 0.017 | 0.187 |
| PSICU 35 | 45.208 | 56 | 0.000 | 0.848 |
| PSICU 41 | 40.333 | 49 | 0.000 | 0.806 |
| PSICU 26 | 65.217 | 53 | 0.019 | 0.121 |

### 5.2.3 Charts of item characteristics

### 5.2.4 Model and item reliability

##
## Marginal empirical reliability: 0.849

## 5.3 IRT for factor 2 of the 6-factor model

### 5.3.1 IRT model fitting and comparison

##
##
## == IRT MODEL COMPARISON ==

|  | Modèle | G2 | AIC | BIC | LogLik |
| --- | --- | --- | --- | --- | --- |
| PCM | PCM | 2798.56 | 10243.68 | 10353.93 | -5096.84 |
| Graded | Graded | 2664.22 | 10119.33 | 10251.64 | -5029.67 |
| RSM | RSM | 2979.46 | 10394.57 | 10438.68 | -5187.29 |
| GPCM | GPCM | 2720.68 | 10175.80 | 10308.11 | -5057.90 |

### 5.3.2 Evaluation of the selected model

##
##
## == ITEM PARAMETERS ( Graded ) ==

|  | a | b1 | b2 | b3 | b4 |
| --- | --- | --- | --- | --- | --- |
| PSICU 34 | 1.385 | -1.398 | -0.535 | 0.685 | 1.984 |
| PSICU 28 | 2.003 | -1.916 | -0.446 | 0.633 | 1.498 |
| PSICU 29 | 1.264 | -1.566 | -0.658 | 0.667 | 2.042 |
| PSICU 7 | 1.927 | -3.524 | -1.555 | -0.147 | 1.238 |
| PSICU 45 | 1.037 | -1.551 | -0.792 | 0.982 | 2.623 |
| PSICU 5 | 1.464 | -2.631 | -1.150 | 0.200 | 1.414 |

##
##
## == ASSESSMENT OF MODEL FIT ==

| item | S_X2 | df.S_X2 | RMSEA.S_X2 | p.S_X2 |
| --- | --- | --- | --- | --- |
| PSICU 34 | 88.732 | 45 | 0.040 | 0.000 |
| PSICU 28 | 46.553 | 38 | 0.019 | 0.161 |
| PSICU 29 | 53.387 | 45 | 0.018 | 0.183 |
| PSICU 7 | 19.726 | 30 | 0.000 | 0.923 |
| PSICU 45 | 86.279 | 47 | 0.037 | 0.000 |
| PSICU 5 | 47.514 | 43 | 0.013 | 0.294 |

### 5.3.3 Charts of item characteristics

### 5.3.4 Model and item reliability

##
## Marginal empirical reliability: 0.8

## 5.4 IRT for factor 3 of the 6-factor model

### 5.4.1 IRT model fitting and comparison

##
##
## == IRT MODEL COMPARISON ==

|  | Modèle | G2 | AIC | BIC | LogLik |
| --- | --- | --- | --- | --- | --- |
| PCM | PCM | 1276.06 | 8106.14 | 8198.76 | -4032.07 |
| Graded | Graded | 1208.08 | 8046.17 | 8156.42 | -3998.08 |
| RSM | RSM | 1619.36 | 8425.44 | 8465.14 | -4203.72 |
| GPCM | GPCM | 1249.51 | 8087.60 | 8197.85 | -4018.80 |

### 5.4.2 Evaluation of the selected model

##
##
## == ITEM PARAMETERS ( Graded ) ==

|  | a | b1 | b2 | b3 | b4 |
| --- | --- | --- | --- | --- | --- |
| PSICU 18 | 2.214 | -2.721 | -0.908 | 0.351 | 1.421 |
| PSICU 40 | 1.679 | -2.876 | -0.425 | 0.840 | 2.114 |
| PSICU 37 | 1.400 | -1.247 | -0.651 | 0.624 | 1.770 |
| PSICU 16 | 1.751 | -1.588 | -0.991 | 0.244 | 1.312 |
| PSICU 25 | 1.478 | -3.215 | -1.146 | 0.348 | 1.844 |

##
##
## == ASSESSMENT OF MODEL FIT ==

| item | S_X2 | df.S_X2 | RMSEA.S_X2 | p.S_X2 |
| --- | --- | --- | --- | --- |
| PSICU 18 | 14.634 | 25 | 0.000 | 0.950 |
| PSICU 40 | 55.169 | 27 | 0.041 | 0.001 |
| PSICU 37 | 68.641 | 35 | 0.040 | 0.001 |
| PSICU 16 | 33.469 | 32 | 0.009 | 0.396 |
| PSICU 25 | 35.139 | 30 | 0.017 | 0.238 |

### 5.4.3 Charts of item characteristics

### 5.4.4 Model and item reliability

##
## Marginal empirical reliability: 0.8

## 5.5 IRT for factor 4 of the 6-factor model

### 5.5.1 IRT model fitting and comparison

##
##
## == IRT MODEL COMPARISON ==

|  | Modèle | G2 | AIC | BIC | LogLik |
| --- | --- | --- | --- | --- | --- |
| PCM | PCM | 182.02 | 5080.23 | 5137.57 | -2527.12 |
| Graded | Graded | 167.20 | 5069.41 | 5135.57 | -2519.71 |
| RSM | RSM | 263.47 | 5149.69 | 5180.56 | -2567.84 |
| GPCM | GPCM | 163.63 | 5065.85 | 5132.00 | -2517.92 |

### 5.5.2 Evaluation of the selected model

##
##
## == ITEM PARAMETERS ( GPCM ) ==

|  | a | b1 | b2 | b3 | b4 |
| --- | --- | --- | --- | --- | --- |
| PSICU 50 | 0.664 | -1.971 | 0.881 | 1.388 | 0.264 |
| PSICU 20 | 2.121 | -1.281 | -0.183 | 0.582 | 0.955 |
| PSICU 23 | 0.719 | -3.807 | 0.802 | 1.469 | 1.058 |

##
##
## == ASSESSMENT OF MODEL FIT ==

| item | S_X2 | df.S_X2 | RMSEA.S_X2 | p.S_X2 |
| --- | --- | --- | --- | --- |
| PSICU 50 | 16.355 | 11 | 0.028 | 0.128 |
| PSICU 20 | 30.280 | 11 | 0.054 | 0.001 |
| PSICU 23 | 20.911 | 10 | 0.042 | 0.022 |

### 5.5.3 Charts of item characteristics

### 5.5.4 Model and item reliability

##
## Marginal empirical reliability: 0.735

## 5.6 IRT for factor 5 of the 6-factor model

### 5.6.1 IRT model fitting and comparison

##
##
## == IRT MODEL COMPARISON ==

|  | Modèle | G2 | AIC | BIC | LogLik |
| --- | --- | --- | --- | --- | --- |
| PCM | PCM | 1727.08 | 8788.59 | 8881.20 | -4373.30 |
| Graded | Graded | 1648.30 | 8717.81 | 8828.06 | -4333.91 |
| RSM | RSM | 1814.78 | 8852.29 | 8891.98 | -4417.15 |
| GPCM | GPCM | 1683.73 | 8753.24 | 8863.49 | -4351.62 |

### 5.6.2 Evaluation of the selected model

##
##
## == ITEM PARAMETERS ( Graded ) ==

|  | a | b1 | b2 | b3 | b4 |
| --- | --- | --- | --- | --- | --- |
| PSICU 17 | 2.617 | -1.438 | -0.433 | 0.376 | 0.985 |
| PSICU 13 | 1.872 | -0.748 | -0.150 | 0.784 | 1.681 |
| PSICU 21 | 1.930 | -0.587 | -0.074 | 0.740 | 1.528 |
| PSICU 38 | 1.580 | -1.007 | -0.291 | 0.610 | 1.534 |
| PSICU 3 | 1.188 | -1.995 | -0.722 | 0.515 | 1.689 |

##
##
## == ASSESSMENT OF MODEL FIT ==

| item | S_X2 | df.S_X2 | RMSEA.S_X2 | p.S_X2 |
| --- | --- | --- | --- | --- |
| PSICU 17 | 25.611 | 31 | 0.000 | 0.740 |
| PSICU 13 | 53.279 | 38 | 0.026 | 0.051 |
| PSICU 21 | 80.901 | 37 | 0.044 | 0.000 |
| PSICU 38 | 63.867 | 40 | 0.031 | 0.010 |
| PSICU 3 | 36.124 | 42 | 0.000 | 0.726 |

### 5.6.3 Charts of item characteristics

### 5.6.4 Model and item reliability

##
## Marginal empirical reliability: 0.822

## 5.7 IRT for factor 6 of the 6-factor model

### 5.7.1 IRT model fitting and comparison

##
##
## == IRT MODEL COMPARISON ==

|  | Modèle | G2 | AIC | BIC | LogLik |
| --- | --- | --- | --- | --- | --- |
| PCM | PCM | 616.53 | 7097.30 | 7172.27 | -3531.65 |
| Graded | Graded | 593.29 | 7080.06 | 7168.26 | -3520.03 |
| RSM | RSM | 756.62 | 7219.38 | 7254.66 | -3601.69 |
| GPCM | GPCM | 606.91 | 7093.67 | 7181.88 | -3526.84 |

### 5.7.2 Evaluation of the selected model

##
##
## == ITEM PARAMETERS ( Graded ) ==

|  | a | b1 | b2 | b3 | b4 |
| --- | --- | --- | --- | --- | --- |
| PSICU 32 | 1.806 | -1.793 | -0.866 | 0.217 | 1.067 |
| PSICU 8 | 1.570 | -1.098 | -0.423 | 0.718 | 1.837 |
| PSICU 15 | 1.447 | -0.667 | -0.089 | 0.617 | 1.368 |
| PSICU 6 | 1.549 | -1.116 | 0.197 | 1.192 | 2.191 |

##
##
## == ASSESSMENT OF MODEL FIT ==

| item | S_X2 | df.S_X2 | RMSEA.S_X2 | p.S_X2 |
| --- | --- | --- | --- | --- |
| PSICU 32 | 22.603 | 23 | 0.000 | 0.484 |
| PSICU 8 | 32.626 | 27 | 0.019 | 0.210 |
| PSICU 15 | 35.900 | 28 | 0.022 | 0.145 |
| PSICU 6 | 30.552 | 25 | 0.019 | 0.204 |

### 5.7.3 Charts of item characteristics

### 5.7.4 Model and item reliability

##
## Marginal empirical reliability: 0.74
