# Supplementary material for: Further analysis and refinements of the perceived stressors in intensive care units (PS-ICU) scale: a French nation-wide cross-sectional multicentre study
Source: Ann Intensive Care. 2025 Nov 20;15:186. doi: 10.1186/s13613-025-01572-7 (PMC12630482; doi:10.1186/s13613-025-01572-7)
Supplement: Supplementary file 4 — Additional file 4. [file 13613_2025_1572_MOESM4_ESM.docx]

**PS-ICU scale English version**

**Perceived stressors in intensive care units.** Below are several situations that may be experienced in intensive care units. For each of these situations: Indicate whether or not you have experienced a specific situation within your unit and, if so, specify your level of stress when you encountered the situation. Respond by circling one number ranging from 0 (never experienced) to 4 (experienced and extremely stressed).

| **Have you experienced this situation within your unit?** | **Never experienced** | **I experienced this situation and...** | | | |
| --- | --- | --- | --- | --- | --- |
|  |  | **I was not at all stressed** | **I was a little stressed** | **I was rather stressed** | **I was extremely stressed** |
| 1. Socially isolated end-of-life patient or one with no immediate family | 0 | 1 | 2 | 3 | 4 |
| 2. Colleague not doing his/her work properly | 0 | 1 | 2 | 3 | 4 |
| 3. Lack of recognition (from the patient, the family, the team, the hierarchy) | 0 | 1 | 2 | 3 | 4 |
| 4. Contradictory information given by other healthcare professionals to the family | 0 | 1 | 2 | 3 | 4 |
| *5. Caring for young patients or who have young children | 0 | 1 | 2 | 3 | 4 |
| *6. Shortage of beds in the unit | 0 | 1 | 2 | 3 | 4 |
| *7. Families’ distress or emotions | 0 | 1 | 2 | 3 | 4 |
| *8. Inadequate or under-equipped healthcare space or defective materials | 0 | 1 | 2 | 3 | 4 |
| 9. Disagreement and/or lack of coordination with other units concerning a patient’s treatment | 0 | 1 | 2 | 3 | 4 |
| *10. Family conflict or disagreement concerning the patient’s treatment plan | 0 | 1 | 2 | 3 | 4 |
| 11. Too many professionals around the patient in an emergency situation | 0 | 1 | 2 | 3 | 4 |
| 12. Incomprehensible or unnecessary care relative to the patient’s situation | 0 | 1 | 2 | 3 | 4 |
| *13. Difficulty to find my place, have my skills recognized, or voice my opinion within the team | 0 | 1 | 2 | 3 | 4 |
| 14. Noisy environment | 0 | 1 | 2 | 3 | 4 |
| *15. Lack of support from the administration | 0 | 1 | 2 | 3 | 4 |
| *16. Risk of error, fear of doing a poor job | 0 | 1 | 2 | 3 | 4 |
| *17. Negative atmosphere prevailing in the team, gossip, rumours within the team | 0 | 1 | 2 | 3 | 4 |
| *18. Having to execute care tasks quickly in emergency cases | 0 | 1 | 2 | 3 | 4 |
| 19. Plaintive patient who makes many requests | 0 | 1 | 2 | 3 | 4 |
| *20. Working pace or working hours hardly compatible with family or social life | 0 | 1 | 2 | 3 | 4 |
| *21. Conflicts with members of the healthcare team | 0 | 1 | 2 | 3 | 4 |
| 22. Not being able to communicate with the patient | 0 | 1 | 2 | 3 | 4 |
| *23. Schedule changes, overtime | 0 | 1 | 2 | 3 | 4 |
| 24. Working while experiencing difficult personal events | 0 | 1 | 2 | 3 | 4 |
| 25. Patient who deteriorates in an unexpected or unexplained manner | 0 | 1 | 2 | 3 | 4 |
| *26. Family whose beliefs or lifestyle are contradictory with my values or the functioning of the unit | 0 | 1 | 2 | 3 | 4 |
| *27. Family’s misunderstanding of the gravity of the diagnosis or the prognosis of the patient | 0 | 1 | 2 | 3 | 4 |
| *28. Series of patient deaths in the unit over a short period | 0 | 1 | 2 | 3 | 4 |
| *29. Patient who makes me think of someone close to me or of myself | 0 | 1 | 2 | 3 | 4 |
| 30. Powerlessness or incompetence in supporting families | 0 | 1 | 2 | 3 | 4 |
| 31. Changes in the modalities of care or the therapy project depending on the doctor responsible for the patient | 0 | 1 | 2 | 3 | 4 |
| *32. Lack of staff | 0 | 1 | 2 | 3 | 4 |
| 33. Non-supportive, aggressive or delirious patient | 0 | 1 | 2 | 3 | 4 |
| *34. Death of a patient with whom I had developed special ties | 0 | 1 | 2 | 3 | 4 |
| *35. Family which does not trust me or does not trust the team | 0 | 1 | 2 | 3 | 4 |
| 36. Continuous and heavy workload | 0 | 1 | 2 | 3 | 4 |
| *37. Having to perform tasks for which I lack knowledge or skills | 0 | 1 | 2 | 3 | 4 |
| *38. Being assessed or judged by other members of the team | 0 | 1 | 2 | 3 | 4 |
| 39. Time spent on administrative tasks at the expense of care giving time | 0 | 1 | 2 | 3 | 4 |
| *40. Treating complex or serious pathologies | 0 | 1 | 2 | 3 | 4 |
| *41. Caring for a patient who should not be treated by the ICU | 0 | 1 | 2 | 3 | 4 |
| *42. Uncertainty concerning the diagnosis or the therapy project of the patient | 0 | 1 | 2 | 3 | 4 |
| 43. Lack of respect for the patient (against his/her wishes, his/her integrity, his/her situation, etc.) | 0 | 1 | 2 | 3 | 4 |
| 44. Patient suffering physically or psychologically | 0 | 1 | 2 | 3 | 4 |
| 45. Having to announce a bad diagnosis to the patient or his/her family or be present when such a diagnosis is announced | 0 | 1 | 2 | 3 | 4 |
| 46. Lack of equality in the distribution of tasks among healthcare professionals | 0 | 1 | 2 | 3 | 4 |
| 47. Unsuitable or under-equipped space to receive families | 0 | 1 | 2 | 3 | 4 |
| 48. Accumulated workloads resulting from clinical activity, training, research or teaching | 0 | 1 | 2 | 3 | 4 |
| 49. Decision to stop or reduce treatment | 0 | 1 | 2 | 3 | 4 |
| 50. Being on call or working nights | 0 | 1 | 2 | 3 | 4 |


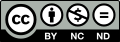
*Note*. *Items of the 26-item version of the scale: Factor 1, Factor 2, Factor 3, Factor 4, Factor 5, Factor 6.

**Echelle PS-ICU version française**

**Stresseurs perçus en réanimation.** Vous trouverez ci-dessous plusieurs situations professionnelles pouvant être vécues en réanimation. Pour chacune de ces situations : indiquez si vous l’avez vécue au sein de votre service et, si oui, précisez à quel point vous avez ressenti du stress face à cette situation. Pour répondre, entourez un chiffre allant de 0 (non vécu(e)) à 4 (vécu(e) et très stressé(e)).

| **Avez-vous vécu cette situation au sein de votre service ?** | **Non vécu(e)** | **J’ai vécu cette situation et…** | | | | | |
| --- | --- | --- | --- | --- | --- | --- | --- |
|  |  | **je n’ai pas du tout été stressé(e)** | **j’ai été  un peu stressé(e)** | **j’ai été assez stressé(e)** | **j’ai été très stressé(e)** | | |
| 1. Patient en fin de vie isolé socialement ou familialement | 0 | 1 | 2 | 3 | 4 | | |
| 2. Collègue qui ne fait pas correctement son travail | 0 | 1 | 2 | 3 | 4 | | |
| 3. Manque de reconnaissance (de la part du patient, de la famille, de l’équipe, la hiérarchie) | 0 | 1 | 2 | 3 | 4 | | |
| 4. Informations contradictoires données par d'autres soignants à la famille | 0 | 1 | 2 | 3 | 4 | | |
| *5. Prise en charge de patients jeunes ou ayant des enfants en bas âge | 0 | 1 | 2 | 3 | 4 | | |
| *6. Manque de lits dans le service | 0 | 1 | 2 | 3 | 4 | | |
| *7. Détresse ou émotions des familles | 0 | 1 | 2 | 3 | 4 | | |
| *8. Espace de soin inadapté, sous-équipé ou matériel défectueux | 0 | 1 | 2 | 3 | 4 | | |
| 9. Désaccord ou manque de coordination avec les autres services sur la prise en charge du patient | 0 | 1 | 2 | 3 | 4 | | |
| *10. Conflit ou désaccord au sein de la famille concernant la prise en charge du patient | 0 | 1 | 2 | 3 | 4 | | |
| 11. Afflux trop important de professionnels autour du malade dans une situation d’urgence | 0 | 1 | 2 | 3 | 4 | | |
| 12. Réaliser des soins incompréhensibles ou inutiles au regard de la situation du patient | 0 | 1 | 2 | 3 | 4 | | |
| *13. Difficulté à faire ma place, à faire reconnaître mes compétences ou faire entendre mon avis au sein de l'équipe | 0 | 1 | 2 | 3 | 4 | | |
| 14. Environnement bruyant | 0 | 1 | 2 | 3 | 4 | | |
| *15. Absence de soutien de l'administration | 0 | 1 | 2 | 3 | 4 | | |
| *16. Risque d'erreur, peur de mal faire son travail | 0 | 1 | 2 | 3 | 4 | | |
| *17. Mauvaise ambiance dans l'équipe, commérages, rumeurs au sein de l'équipe | 0 | 1 | 2 | 3 | 4 | | |
| *18. Devoir exécuter rapidement des soins en situation d'urgence | 0 | 1 | 2 | 3 | 4 | | |
| 19. Patient plaintif, exprimant de nombreuses demandes | 0 | 1 | 2 | 3 | 4 | | |
| *20. Rythme ou horaire de travail difficilement compatible avec la vie de famille ou la vie sociale | 0 | 1 | 2 | 3 | 4 | | |
| *21. Conflits avec des membres de l'équipe soignante | 0 | 1 | 2 | 3 | 4 | | |
| 22. Ne pas pouvoir communiquer avec le patient | 0 | 1 | 2 | 3 | 4 | | |
| *23. Changement de planning, heures supplémentaires | 0 | 1 | 2 | 3 | | 4 |  |
| 24. Travailler alors que l'on vit des événements personnels difficiles | 0 | 1 | 2 | 3 | | 4 |  |
| 25. Patient qui se dégrade de manière inattendue et inexpliquée | 0 | 1 | 2 | 3 | | 4 |  |
| *26. Famille dont les croyances ou le mode de vie sont en contradiction avec mes valeurs ou le mode de fonctionnement du service | 0 | 1 | 2 | 3 | | 4 |  |
| *27. Mauvaise compréhension par la famille de la gravité du diagnostic ou du pronostic du patient | 0 | 1 | 2 | 3 | | 4 |  |
| *28. Enchainement de décès de patients dans le service sur une période courte | 0 | 1 | 2 | 3 | | 4 |  |
| *29. Patient qui me fait penser à un de mes proches ou à moi-même | 0 | 1 | 2 | 3 | | 4 |  |
| 30. Impuissance ou incompétence dans l'accompagnement des familles | 0 | 1 | 2 | 3 | | 4 |  |
| 31. Changement des modalités de soins ou du projet thérapeutique en fonction du médecin responsable du patient | 0 | 1 | 2 | 3 | | 4 |  |
| *32.  Manque de personnel | 0 | 1 | 2 | 3 | | 4 |  |
| 33.  Patient non conciliant, agressif ou délirant | 0 | 1 | 2 | 3 | | 4 |  |
| *34.  Mort d'un patient avec lequel j'avais créé des liens particuliers | 0 | 1 | 2 | 3 | | 4 |  |
| *35.  Famille qui ne me fait pas confiance ou qui ne fait pas confiance à l'équipe | 0 | 1 | 2 | 3 | | 4 |  |
| 36.  Charge de travail importante et permanente | 0 | 1 | 2 | 3 | | 4 |  |
| *37.  Devoir effectuer des tâches pour lesquelles je manque de connaissances ou de compétences | 0 | 1 | 2 | 3 | | 4 |  |
| *38.  Être évalué ou jugé par les autres membres de l'équipe | 0 | 1 | 2 | 3 | | 4 |  |
| 39.  Temps consacré à des tâches administratives au détriment du temps de soins | 0 | 1 | 2 | 3 | | 4 |  |
| *40.  Prise en charge de pathologies graves ou complexes | 0 | 1 | 2 | 3 | | 4 |  |
| *41.  M'occuper d'un patient dont les soins ne relèvent pas de la réanimation | 0 | 1 | 2 | 3 | | 4 |  |
| *42.  Incertitude du diagnostic ou du projet thérapeutique du patient | 0 | 1 | 2 | 3 | | 4 |  |
| 43.  Manque de respect vis-à-vis du patient (vis-à-vis de ses volontés, de son intégrité, sa situation, etc.) | 0 | 1 | 2 | 3 | | 4 |  |
| 44.  Patient qui souffre physiquement ou psychiquement | 0 | 1 | 2 | 3 | | 4 |  |
| 45.  Devoir annoncer au patient ou à la famille un diagnostic de maladie grave ou être présent lors de l’annonce d’un tel diagnostic | 0 | 1 | 2 | 3 | | 4 |  |
| 46.  Manque d’égalité dans la répartition du travail entre professionnels | 0 | 1 | 2 | 3 | | 4 |  |
| 47.  Espace d'accueil des familles inadapté ou sous-équipé | 0 | 1 | 2 | 3 | | 4 |  |
| 48.  Cumul de la charge de travail liée à l’activité clinique, la formation, la recherche ou l’enseignement | 0 | 1 | 2 | 3 | | 4 |  |
| 49.  Prise de décisions d’arrêt ou limitation de traitement | 0 | 1 | 2 | 3 | | 4 |  |
| *50.  Gardes ou travail de nuit | 0 | 1 | 2 | 3 | | 4 |  |

*Note*. *Items de la version en 26 items : Facteur 1, Facteur 2, Facteur 3, Facteur 4, Facteur 5, Facteur 6**.**


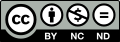


**Escala PS-ICU versión española**

**Estresores percibidos en la unidad de reanimación.** A continuación, se presentan varias situaciones profesionales que pueden vivirse en reanimación. Para cada una de estas situaciones: indique si alguna vez las ha vivido en su servicio y, si es así, especifique hasta qué punto ha sentido estrés frente a dicha situación. Para responder, rodee con un círculo un número de 0 (nunca la he vivido) hasta 4 (he vivido esta situación y he sentido mucho estrés).

| **¿Ha vivido alguna vez esta situación en su servicio?** | **Nunca la he vivido** | **He vivido esta situación y…** | | | |
| --- | --- | --- | --- | --- | --- |
|  |  | **no he sentido nada de estrés** | **he sentido un poco de estrés** | **he sentido bastante estrés** | **he sentido mucho estrés** |
| 1. Paciente en etapa final de su vida aislado social o familiarmente | 0 | 1 | 2 | 3 | 4 |
| 2. Compañero/a que no realiza correctamente su trabajo | 0 | 1 | 2 | 3 | 4 |
| 3. Falta de reconocimiento (de parte del paciente, la familia, el equipo o la jerarquía) | 0 | 1 | 2 | 3 | 4 |
| 4. Informaciones contradictorias dadas a la familia por parte de otros cuidadores | 0 | 1 | 2 | 3 | 4 |
| *5. Tratamiento de pacientes jóvenes o que tienen niños pequeños | 0 | 1 | 2 | 3 | 4 |
| *6. Falta de camas en el servicio | 0 | 1 | 2 | 3 | 4 |
| *7. Desamparo o emociones de las familias | 0 | 1 | 2 | 3 | 4 |
| *8. Espacio de cuidados inadaptado, sin equipamiento suficiente o material defectuoso | 0 | 1 | 2 | 3 | 4 |
| 9. Desacuerdo o falta de coordinación con los demás servicios sobre el tratamiento del paciente | 0 | 1 | 2 | 3 | 4 |
| *10. Conflicto o desacuerdo entre los miembros de la familia sobre el tratamiento del paciente | 0 | 1 | 2 | 3 | 4 |
| 11. Afluencia demasiado importante de profesionales alrededor del paciente en una situación de emergencia | 0 | 1 | 2 | 3 | 4 |
| 12. Realizar cuidados incomprensibles o inútiles con respecto a la situación del paciente | 0 | 1 | 2 | 3 | 4 |
| *13. Dificultad para hacerse un sitio, para que reconozcan mis competencias o para que escuchen mi opinión dentro del equipo | 0 | 1 | 2 | 3 | 4 |
| 14. Entorno ruidoso | 0 | 1 | 2 | 3 | 4 |
| 15. Ausencia de apoyo por parte de la administración | 0 | 1 | 2 | 3 | 4 |
| *16. Riesgo de error, miedo a hacer mal mi trabajo | 0 | 1 | 2 | 3 | 4 |
| *17. Mal ambiente en el equipo, chismes, rumores dentro del equipo | 0 | 1 | 2 | 3 | 4 |
| *18. Tener que ejecutar rápidamente cuidados en situación de emergencia | 0 | 1 | 2 | 3 | 4 |
| 19. Paciente que se queja y pide muchas cosas | 0 | 1 | 2 | 3 | 4 |
| *20. Ritmo u horario de trabajo difícil de compatibilizar con la vida familiar o la vida social | 0 | 1 | 2 | 3 | 4 |
| *21. Conflictos con miembros del equipo médico y sanitario | 0 | 1 | 2 | 3 | 4 |
| 22. No poder comunicar con el paciente | 0 | 1 | 2 | 3 | 4 |
| *23. Cambios en la planificación, horas extras | 0 | 1 | 2 | 3 | 4 |
| 24. Trabajar mientras vivo acontecimientos personales difíciles | 0 | 1 | 2 | 3 | 4 |
| 25. Paciente que se degrada de manera inesperada e inexplicada | 0 | 1 | 2 | 3 | 4 |
| *26. Familia cuyas creencias o estilo de vida son contradictorios con mis valores o el modo de funcionamiento del servicio | 0 | 1 | 2 | 3 | 4 |
| *27. Mala comprensión por parte de la familia sobre la gravedad del diagnóstico o del pronóstico del paciente | 0 | 1 | 2 | 3 | 4 |
| *28. Cadena de fallecimientos de pacientes en el servicio en un periodo corto | 0 | 1 | 2 | 3 | 4 |
| *29. Paciente que me recuerda a uno de mis familiares o a mí mismo/a | 0 | 1 | 2 | 3 | 4 |
| 30. Impotencia o incompetencia en el acompañamiento de las familias | 0 | 1 | 2 | 3 | 4 |
| 31. Cambio de las modalidades de cuidados o del proyecto terapéutico en función del médico responsable del paciente | 0 | 1 | 2 | 3 | 4 |
| *32. Falta de personal | 0 | 1 | 2 | 3 | 4 |
| 33. Paciente que no colabora, agresivo o delirante | 0 | 1 | 2 | 3 | 4 |
| 34. Muerte de un paciente con el cual había creado vínculos especiales | 0 | 1 | 2 | 3 | 4 |
| *35. Familia que no confía en mí o que no confía en el equipo | 0 | 1 | 2 | 3 | 4 |
| 36. Carga de trabajo significativa y permanente | 0 | 1 | 2 | 3 | 4 |
| *37. Tener que efectuar tareas para las cuáles me faltan conocimientos o competencias | 0 | 1 | 2 | 3 | 4 |
| 38. Ser evaluado o juzgado por los otros miembros del equipo | 0 | 1 | 2 | 3 | 4 |
| *39. Tiempo dedicado a tareas administrativas en detrimento del tiempo de cuidados | 0 | 1 | 2 | 3 | 4 |
| *40. Tratamiento de patologías graves o complejas | 0 | 1 | 2 | 3 | 4 |
| *41. Ocuparme de un paciente cuyos cuidados no entran en el ámbito de la reanimación | 0 | 1 | 2 | 3 | 4 |
| *42. Incertidumbre sobre el diagnóstico o el proyecto terapéutico del paciente | 0 | 1 | 2 | 3 | 4 |
| 43. Falta de respeto hacia el paciente (sobre sus voluntades, su integridad, su situación, etc.) | 0 | 1 | 2 | 3 | 4 |
| 44. Paciente que sufre física o psíquicamente | 0 | 1 | 2 | 3 | 4 |
| 45. Tener que comunicar al paciente o a la familia un diagnóstico desfavorable o estar presente cuando se comunica un diagnóstico desfavorable | 0 | 1 | 2 | 3 | 4 |
| 46. Falta de igualdad en la distribución del trabajo entre profesionales | 0 | 1 | 2 | 3 | 4 |
| 47. Espacio de acogida de las familias inadaptado o sin equipamiento suficiente | 0 | 1 | 2 | 3 | 4 |
| 48. Acumulación de la carga de trabajo relacionada con la actividad clínica, la formación, la investigación o la enseñanza | 0 | 1 | 2 | 3 | 4 |
| 49. Toma de decisiones sobre la interrupción o la limitación del tratamiento | 0 | 1 | 2 | 3 | 4 |
| *50. Guardias o trabajo de noche | 0 | 1 | 2 | 3 | 4 |


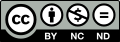
*Nota*. *Ítems de la versión de 26 ítems de la escala: Factor 1, Factor 2, Factor 3, Factor 4, Factor 5, Factor 6.

**Scala PS-ICU versione italiana**

**Fattori percepiti come fonti di stress nelle unità di Terapia intensiva.** Elencate qui di seguito, troverà differenti situazioni professionali che possono essere vissute in un reparto di Terapia intensiva. Per ognuna di esse, indichi se ha già dovuto confrontarsi con questa situazione nel Suo reparto e, in tal caso, precisi in che misura si è sentito stressato a causa di questa situazione. Per rispondere, assegni una cifra da 0 (non ho mai dovuto confrontarmi a questa situazione) a 4 (mi sono già trovato/a in questa situazione e mi ha molto stressato/a).

| **Ha già dovuto confrontarsi con questa situazione nel Suo reparto?** | **No, mai** | **Sì, mi sono già dovuto confrontare con questa situazione e...** | | | |
| --- | --- | --- | --- | --- | --- |
|  |  | **non mi ha affatto stressato/a** | **mi ha un po’ stressato/a** | **mi ha stressato/a abbastanza** | **mi ha  molto stressato/a** |
| 1. Paziente in fin di vita isolato socialmente o dal punto di vista familiare | 0 | 1 | 2 | 3 | 4 |
| 2. Collega che non svolge correttamente il suo lavoro | 0 | 1 | 2 | 3 | 4 |
| 3. Mancanza di riconoscenza (da parte del paziente, della famiglia, dell’equipe, della gerarchia) | 0 | 1 | 2 | 3 | 4 |
| 4. Informazioni contraddittorie date alla famiglia da altre figure professionali sanitarie | 0 | 1 | 2 | 3 | 4 |
| *5. Presa in carico di pazienti giovani o con figli piccoli | 0 | 1 | 2 | 3 | 4 |
| *6. Mancanza di letti in reparto | 0 | 1 | 2 | 3 | 4 |
| *7. Difficoltà psicologiche o emozionali dei familiari | 0 | 1 | 2 | 3 | 4 |
| *. Spazi di cura inadatti, carenza di organico o materiale difettoso | 0 | 1 | 2 | 3 | 4 |
| 9. Disaccordo o mancanza di coordinamento con gli altri reparti per la presa in carico del paziente | 0 | 1 | 2 | 3 | 4 |
| *10. Conflitto o disaccordo nell’ambito della famiglia riguardo la presa in carico del paziente | 0 | 1 | 2 | 3 | 4 |
| 11. Afflusso troppo importante di professionisti attorno al malato in una situazione di urgenza | 0 | 1 | 2 | 3 | 4 |
| 12. Somministrazione di cure incomprensibili o inutili in relazione alla situazione del paziente | 0 | 1 | 2 | 3 | 4 |
| *13. Difficoltà a trovare il mio ruolo, a far riconoscere le mie competenze e/o il mio parere nell’ambito dell’equipe | 0 | 1 | 2 | 3 | 4 |
| 14. Ambiente rumoroso | 0 | 1 | 2 | 3 | 4 |
| *15. Assenza di sostegno da parte dell’amministrazione | 0 | 1 | 2 | 3 | 4 |
| *16. Rischio di errore, paura di fare male il mio lavoro | 0 | 1 | 2 | 3 | 4 |
| *17. Cattiva atmosfera nell’ambito dell’equipe (pettegolezzi, voci, ecc.) | 0 | 1 | 2 | 3 | 4 |
| *18. Dover somministrare rapidamente delle cure in situazione di urgenza | 0 | 1 | 2 | 3 | 4 |
| 19. Paziente che si lamenta, che pone molteplici domande | 0 | 1 | 2 | 3 | 4 |
| *20. Ritmo o orari di lavoro difficilmente compatibili con la vita di famiglia o la vita sociale | 0 | 1 | 2 | 3 | 4 |
| *21. Conflitti con i membri dell’equipe | 0 | 1 | 2 | 3 | 4 |
| 22. Non poter comunicare con il paziente | 0 | 1 | 2 | 3 | 4 |
| *23. Cambiamento dei turni, prestazioni straordinarie | 0 | 1 | 2 | 3 | 4 |
| 24. Lavorare mentre si stanno vivendo momenti personali difficili | 0 | 1 | 2 | 3 | 4 |
| 25. Paziente che peggiora in maniera inattesa o inspiegabile | 0 | 1 | 2 | 3 | 4 |
| *26. Famiglia le cui convinzioni o il cui modo di vita sono in contraddizione con i miei valori o con il funzionamento del reparto | 0 | 1 | 2 | 3 | 4 |
| *27. Cattiva comprensione da parte della famiglia della gravità della diagnosi o della prognosi del paziente | 0 | 1 | 2 | 3 | 4 |
| *28. Serie di decessi di pazienti nel reparto in un arco limitato di tempo | 0 | 1 | 2 | 3 | 4 |
| *29. Paziente che mi fa pensare a uno dei miei parenti o a me stesso | 0 | 1 | 2 | 3 | 4 |
| 30. Impotenza o incompetenza nell’accompagnamento delle famiglie | 0 | 1 | 2 | 3 | 4 |
| 31. Cambiamento nella somministrazione delle cure o del progetto terapeutico in funzione del medico responsabile del paziente | 0 | 1 | 2 | 3 | 4 |
| *32. Mancanza di personale | 0 | 1 | 2 | 3 | 4 |
| 33. Paziente non collaborativo, aggressivo o delirante | 0 | 1 | 2 | 3 | 4 |
| *34. Morte di un paziente con il quale avevo instaurato un legame particolare | 0 | 1 | 2 | 3 | 4 |
| *35. Famiglia che non ha fiducia in me o nell’equipe | 0 | 1 | 2 | 3 | 4 |
| 36. Sovraccarico di lavoro importante e permanente | 0 | 1 | 2 | 3 | 4 |
| *37. Dover effettuare dei compiti che non conosco o per i quali manco di competenze | 0 | 1 | 2 | 3 | 4 |
| *38. Essere valutato o giudicato da altri membri dell’equipe | 0 | 1 | 2 | 3 | 4 |
| 39. Tempo dedicato a incombenze di tipo amministrativo sottratto a quello dedicato alle cure | 0 | 1 | 2 | 3 | 4 |
| *40. Presa in carico di patologie gravi o complesse | 0 | 1 | 2 | 3 | 4 |
| *41. Occuparmi di un paziente le cui cure non sono di competenza del reparto di Rianimazione | 0 | 1 | 2 | 3 | 4 |
| *42. Incertezza della diagnosi o del progetto terapeutico del paziente | 0 | 1 | 2 | 3 | 4 |
| 43. Mancanza di rispetto per il paziente (riguardo alle sue volontà, alla sua integrità, alla sua situazione, ecc.) | 0 | 1 | 2 | 3 | 4 |
| 44. Paziente che soffre fisicamente o psicologicamente | 0 | 1 | 2 | 3 | 4 |
| 45. Dover annunciare al paziente o alla famiglia una diagnosi di malattia grave o essere presente al momento dell’annuncio di una diagnosi di malattia grave | 0 | 1 | 2 | 3 | 4 |
| 46. Mancanza di equità nella distribuzione del lavoro tra colleghi | 0 | 1 | 2 | 3 | 4 |
| 47. Spazio di accoglienza per le famiglie inadatto o insufficiente | 0 | 1 | 2 | 3 | 4 |
| 48. Accumulo del carico di lavoro legato all’attività clinica, alla formazione, alla ricerca o all’insegnamento | 0 | 1 | 2 | 3 | 4 |
| 49. Prendere la decisione di interrompere o ridurre il trattamento | 0 | 1 | 2 | 3 | 4 |
| *50. Guardie o lavoro di notte | 0 | 1 | 2 | 3 | 4 |


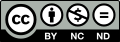
*Nota*. *Item della versione a 26 item della scala : Fattore 1, Fattore 2, Fattore 3, Fattore 4, Fattore 5, Fattore 6.

**中文版ICU医护人员感知压力源量表 (translated and adapted by Gao et al., 2025)**

**引述: Gao Y, Chen X, Zhang S, et al. Confirmatory factor analyses of the Mandarin Chinese version of the perceived stressors in intensive care units among healthcare professionals. *Front Public Health*. 2025;13:1434440. doi:10.3389/fpubh.2025.1434440**

**重症监护室的压力感知。**以下是重症监护病房内可能遇到的一些情况。对于每种情况，请指出你是否在你的病房内经历过这些特定的情况。如果您有经历过的话，请选择遇到该情况时您的压力水平（1分~54分。其中，1分代表从未经历过，5分代表压力极大。

| 题目 | 从未经历过 | 没有任何压力 | 有一点压力 | 压力相当大 | 压力极大 |
| --- | --- | --- | --- | --- | --- |
| 1. 照护与社会隔离的临终患者或没有直系亲属的患者 | 0 | 1 | 2 | 3 | 4 |
| 2. 同事没有妥当地完成当班工作，导致护理/治疗缺乏连贯性 | 0 | 1 | 2 | 3 | 4 |
| 3. 缺乏认同感, 如来自患者、家属、团队及上级的认同感 | 0 | 1 | 2 | 3 | 4 |
| 4.与其他医务人员向家属提供的信息相矛盾 | 0 | 1 | 2 | 3 | 4 |
| *5. 照护病情重且预后差的年轻患者或有年幼子女的患者 | 0 | 1 | 2 | 3 | 4 |
| *6. 病房内床位不足 | 0 | 1 | 2 | 3 | 4 |
| *7. 面对患者家属痛苦或期待的表情 | 0 | 1 | 2 | 3 | 4 |
| *8. 医疗空间不足、设备缺乏，或耗材量大而无法满足患者需求 | 0 | 1 | 2 | 3 | 4 |
| 9. 关于患者治疗，与其他科室医护人员有分歧和/或缺乏配合 | 0 | 1 | 2 | 3 | 4 |
| 10. 抢救时，患者周围聚集太多医护人员而干扰治疗 | 0 | 1 | 2 | 3 | 4 |
| 11. 对患者进行医护人员难以理解或不必要的护理， 如家属强烈要求的护理操作 | 0 | 1 | 2 | 3 | 4 |
| *12. 在团队中难以找到自我位置、表达自我观点（如治疗理念被同事完全否决），或自我技能难以得到认可 | 0 | 1 | 2 | 3 | 4 |
| 13. 环境嘈杂 | 0 | 1 | 2 | 3 | 4 |
| *14. 缺乏来自管理层的支持 | 0 | 1 | 2 | 3 | 4 |
| *15. 存在出错的风险，担心做不好工作 | 0 | 1 | 2 | 3 | 4 |
| *16. 团队内消极氛围弥漫，存在闲言碎语甚至流言蜚语 | 0 | 1 | 2 | 3 | 4 |
| 17. 面对提出过多无理要求的患者及其家属 | 0 | 1 | 2 | 3 | 4 |
| *18. 工作节奏和工作时间很难与家庭及社会生活相协调 | 0 | 1 | 2 | 3 | 4 |
| *19. 与同事起冲突 | 0 | 1 | 2 | 3 | 4 |
| 20. 与患者无法顺利的沟通 | 0 | 1 | 2 | 3 | 4 |
| *21. 计划有改变，如不在计划范围之内加班/非听班时间段内加班/下班后接到科室与工作有关电话等 | 0 | 1 | 2 | 3 | 4 |
| 22. 经历个人困难事件（如生理期、身体不舒服、家人生病需要照顾）时仍需工作 | 0 | 1 | 2 | 3 | 4 |
| 23. 面对患者病情变化难以预料或无法解释 | 0 | 1 | 2 | 3 | 4 |
| *24. 患者家属存在一些与自己的价值观不符或与病房工作模式相矛盾的理念或生活方式 | 0 | 1 | 2 | 3 | 4 |
| *25. 关于疾病诊断，患者家属对其严重性或预后存在误解 | 0 | 1 | 2 | 3 | 4 |
| *26. 病房里接受治疗的患者短期内相继去世 | 0 | 1 | 2 | 3 | 4 |
| 27. 无能为力或无法胜任照顾自己的家庭 | 0 | 1 | 2 | 3 | 4 |
| 28. 关于护理模式或治疗方案，其变化取决于主管医生 | 0 | 1 | 2 | 3 | 4 |
| *29. 缺少人手 | 0 | 1 | 2 | 3 | 4 |
| 30. 面对不合作、有攻击性或谵妄的患者 | 0 | 1 | 2 | 3 | 4 |
| *31. 与我建立了特别情感关系的患者去世 | 0 | 1 | 2 | 3 | 4 |
| *32. 患者家属不相信我或团队的专业性 | 0 | 1 | 2 | 3 | 4 |
| 33. 持续而繁重的工作量 | 0 | 1 | 2 | 3 | 4 |
| *34. 必须完成自己没有掌握技能的任务 | 0 | 1 | 2 | 3 | 4 |
| *35. 被团队其他成员或领导评价 | 0 | 1 | 2 | 3 | 4 |
| *36. 处理复杂或严重疾病 | 0 | 1 | 2 | 3 | 4 |
| *37. 治疗或照护非ICU准入指征的患者 | 0 | 1 | 2 | 3 | 4 |
| *38. 患者的诊断或治疗方案存在不确定性 | 0 | 1 | 2 | 3 | 4 |
| 39. 对患者缺乏尊重，如违背其意愿、 处境等进行治疗或护理操作 | 0 | 1 | 2 | 3 | 4 |
| 40. 面对身体或心理上遭受痛苦的患者 | 0 | 1 | 2 | 3 | 4 |
| 41. 不得不向患者或家属宣布不好的诊断， 或宣布诊断时必须在场 | 0 | 1 | 2 | 3 | 4 |
| 42. 医疗专业人员之间任务分配不合理，如奖金分配、 工作安排、学习进修等 | 0 | 1 | 2 | 3 | 4 |
| 43. 在不适合或设施简陋的房间接待患者家属， 如在装有录像的谈话间与患者家属沟通 | 0 | 1 | 2 | 3 | 4 |
| 44. 因参加临床活动、培训、研究或教学而累积的工作负担 | 0 | 1 | 2 | 3 | 4 |
| 45. 做出停止/减少治疗或照护的决定 | 0 | 1 | 2 | 3 | 4 |
| *46. 随时待命或值夜班 | 0 | 1 | 2 | 3 | 4 |

*注。本文发布的 26 个项目版本的量表所包含的项目：
因子 1、因子 2、因子 3、因子 4、因子 5、因子 6。
